# Challenges in Clinical Metaproteomics Highlighted by the Analysis of Acute Leukemia Patients with Gut Colonization by Multidrug-Resistant Enterobacteriaceae

**DOI:** 10.3390/proteomes7010002

**Published:** 2019-01-08

**Authors:** Julia Rechenberger, Patroklos Samaras, Anna Jarzab, Juergen Behr, Martin Frejno, Ana Djukovic, Jaime Sanz, Eva M. González-Barberá, Miguel Salavert, Jose Luis López-Hontangas, Karina B. Xavier, Laurent Debrauwer, Jean-Marc Rolain, Miguel Sanz, Marc Garcia-Garcera, Mathias Wilhelm, Carles Ubeda, Bernhard Kuster

**Affiliations:** 1 Chair of Proteomics and Bioanalytics, Technical University of Munich, 85354 Freising, Germany; julia.rechenberger@tum.de (J.R.); patroklos.samaras@tum.de (P.S.); anna.jarzab@tum.de (A.J.); martin.frejno@tum.de (M.F.); 2 Bavarian Center for Biomolecular Mass Spectrometry, Technical University of Munich, 85354 Freising, Germany; juergen.behr@tum.de; 3 Centro Superior de Investigación en Salud Pública-FISABIO, 46020 Valencia, Spain; adjukovic@gmail.com; 4 Hospital Universitari i Politècnic La Fe, 46026 Valencia, Spain; sanz_jai@gva.es (J.S.); evamariagonzalezbarbera@gmail.com (E.M.G.-B.); salavert_mig@gva.es (M.S.); lopez_jlu@gva.es (J.L.L.-H.); sanz_mig@gva.es (M.S.); 5 CIBERONC, Instituto Carlos III, 28029 Madrid, Spain; 6 Instituto Gulbenkian de Ciência, 2780 Oeiras, Portugal; kxavier@igc.gulbenkian.pt; 7 Toxalim, Université de Toulouse, INRA, INP-ENVT, INP-EI-Purpan, Université de Toulouse 3 Paul Sabatier, 31027 Toulouse, France; laurent.debrauwer@inra.fr; 8 Axiom Platform, UMR 1331 Toxalim, MetaToul-MetaboHUB, National Infrastructure of Metabolomics and Fluxomics, 31027 Toulouse, France; 9 Aix Marseille Univ, IRD, APHM, MEPHI, IHU-Méditerranée Infection, 13385 Marseille, France; jean-marc.rolain@univ-amu.fr; 10 Department of Fundamental Microbiology, University of Lausanne, 1015 Lausanne, Switzerland; marc.garcia.garcera@gmail.com; 11 Centers of Biomedical Research Network (CIBER) in Epidemiology and Public Health, 28029 Madrid, Spain

**Keywords:** human gut microbiome, metaproteome, data analysis, mass spectrometry, proteomics, clinical proteomics, multi-omics, multidrug-resistant Enterobacteriaceae

## Abstract

The microbiome has a strong impact on human health and disease and is, therefore, increasingly studied in a clinical context. Metaproteomics is also attracting considerable attention, and such data can be efficiently generated today owing to improvements in mass spectrometry-based proteomics. As we will discuss in this study, there are still major challenges notably in data analysis that need to be overcome. Here, we analyzed 212 fecal samples from 56 hospitalized acute leukemia patients with multidrug-resistant Enterobactericeae (MRE) gut colonization using metagenomics and metaproteomics. This is one of the largest clinical metaproteomic studies to date, and the first metaproteomic study addressing the gut microbiome in MRE colonized acute leukemia patients. Based on this substantial data set, we discuss major current limitations in clinical metaproteomic data analysis to provide guidance to researchers in the field. Notably, the results show that public metagenome databases are incomplete and that sample-specific metagenomes improve results. Furthermore, biological variation is tremendous which challenges clinical study designs and argues that longitudinal measurements of individual patients are a valuable future addition to the analysis of patient cohorts.

## 1. Introduction

Research over the past years has established the importance of the gut microbiota for human health and that a disturbed equilibrium is involved in the development of disease [1]. Therefore, scientists have begun characterizing the microbiome of the human gut in healthy and diseased states. Today, most microbiome studies rely on 16S rRNA or shotgun metagenome sequencing to provide a taxonomic description of the microbiome [2]. This does, however, not necessarily reflect proteomic and metabolic activity and, thus, may lack direct functional information. Other omic technologies, such as metaproteomics, metatranscriptomics or metabolomics, can supplement the genomic approaches by providing a molecular view on cellular processes at a more direct functional level [3]. The term metaproteomics was introduced by Wilmes and Bond [4] as well as Rodriguez-Valera [5] as “the large-scale characterization of the entire complement of environmental microbiota at a given point in time”. The main conceptual advantage of metaproteomics is that it can add functional annotations to the description of the microbiome. In addition, metaproteomic can detect proteins from both the host and microbiota simultaneous and, thus, aid in the characterization of host-microbiome interactions [6].

So far, metaproteomic studies have mainly reported variations in the microbiome of healthy people [7,8], changes as a result of antibiotic treatment [9] or the role in chronic gut inflammation, such as Crohn’s disease, inflammatory bowel disease or ulcerative colitis [10,11], as well as obesity [12,13] and diabetes [14]. Here, we present the first metaproteomic study of the gut microbiome in leukemia patients colonized with multidrug-resistant Enterobacteriaceae (MRE). Infections with multidrug-resistant pathogens during hospitalization are becoming critical. Leukemia patients especially are frequently affected since they have a compromised immune system and are regularly exposed to pathogens during extended periods in hospitals. In addition, leukemia patients are frequently treated with antibiotics altering the microbiome that confers resistance to intestinal colonization by exogenous bacteria [15]. Hence, leukemia patients frequently acquire secondary infections during hospitalization. Therefore, it is important to better understand how the gut microbiota may prevent colonization by pathogens and how such information may be utilized in the clinical management of patients.

Although sample preparation protocols in metaproteomics are becoming standardized for clinical studies [16] and the very high performance of liquid chromatography-mass spectrometry (LC-MS/MS) allows the efficient collection of metaproteomic data, the actual analysis of this data is still facing major challenges [17]. These include the lack of truly comprehensive bacterial sequence databases, the demand for considerable computational power, and a shortage of functional and taxonomic annotation [18]. Estimations for fecal samples suggest the potential presence of up to 1,000,000 possible unique proteins [19], leading to sequence databases that are enormous in terms of size. On top of requiring high computational power and large storage systems for data handling and processing, such excessive search spaces result in a significant loss of peptide identification sensitivity. While this issue can be partially addressed by using sample-specific databases generated by genomics or transcriptomics, the absence of annotations for these creates the need for large-scale sequence similarity (e.g., basic local alignment search tool (BLAST) [20]) searches to obtain taxonomic and functional information, which again requires great computational efforts. Furthermore, mapping peptides to proteins and taxa is not trivial due to the many (usually tryptic) peptides that are shared by homologous proteins [21]. In addition, metaproteomic analysis is further challenged by high levels of proteomic sample complexity, dynamic range of the species present and their protein expression levels and, importantly, by large inter- and intra-patient variability [22].

Despite these challenges, analysis of the metaproteome is important. Therefore, we embarked on the first gut metaproteome study of MRE gut colonized leukemia patients. We analyzed 212 fecal samples from 56 patients and provide, to our knowledge, one of the largest clinical metaproteomic datasets to date. In the present manuscript, we report on the analysis of this data, highlight the main challenges and draw some conclusions that may guide scientists and clinicians when designing and conducting metaproteomic projects.

## 2. Materials and Methods

### 2.1. Sampling Process

Fecal samples were collected from November 2013 until April 2015 from acute leukemia patients hospitalized at the Hospital La Fe (Valencia, Spain). All subjects gave their informed consent for inclusion before they participated in the study. The study was conducted in accordance with the Declaration of Helsinki, and the protocol was approved on the 1st of July 2013 by the Ethics Committee of CEIC Dirección General de Salud Pública y Centro Superior de Investigación en Salud Pública (20130515/08). A total of 802 fecal samples were collected from 133 patients. Samples were collected in weekly intervals during the hospitalization period and screened for the presence of MRE. A subset of 56 patients was included in the present study, where MRE colonization was detected in at least one sample. After the first MRE detection, one or more consecutive samples from that patient were included until MRE was not detectable or the patient was discharged. In the case of an MRE recolonization during the same or another hospitalization period samples from these new colonization process were also included following the same criteria. In total, 221 samples from 58 patients matched the inclusion criteria. Due to the limited sample amount, the metaproteome was analyzed for 212 samples from 56 patients. Fresh fecal samples were kept at 4 °C for less than 24 h. Subsequently, three aliquots of each sample were weighed and resuspended in 1 mL of autoclave-sterilized PBS 15% glycerol to preserve viability of bacteria upon freezing and kept at −80 °C until further processing. Three additional aliquots for proteomic and genomic analysis were weighed and directly frozen at −80 °C until further processing. Patient-related metadata was prospectively collected and recorded in a computerized database in Access^®^. This data included information about antibiotic treatments, pathogen presence, gender, age, and type of admission. MRE colonization was determined by culturing fecal samples with Brilliance ESBL agar plates (Oxoid), containing third-generation cephalosporin. Plates were incubated for 24 h at 37 °C. Taxonomic identification of the grown colonies was determined through matrix-assisted laser desorption/ionization - time of flight mass spectrometry (MALDI-TOF MS). If no growth was observed, the plate was left for an additional 24 h at 37 °C to confirm the negative result. In addition, the antibiotic resistant pattern was determined through the Vitek 2 system. Antibiotics tested included amikacin, amoxicillin-clavulanic acid, ampicillin, cefepime, cefotaxime, cefoxitin, ceftazidime, cefuroxime, ciprofloxacin, gentamicin, imipenem, ertapenem, piperacillin-tazobactam, tigecycline, and trimethoprim-sulfamethoxazole (co-trimoxazole). The susceptibility was determined according to the Clinical and Laboratory Standards Institute Guidelines (2016). In addition, resistance to meropenem was evaluated using ETEST antibiotic gradient strips (bioMérieux) in strains isolated from patients who had received meropenem. An isolate was considered multidrug resistant if it was non-susceptible to at least 1 agent in 3 or more antimicrobial categories defined by Magiorakos and co-workers [23].

### 2.2. Sample Preparation

For the proteomic analysis, feces were thawed on ice. For homogenization, 0.5 mL washing buffer (50 mM Na_2_HPO_4_/NAH_2_PO_4_, pH 8.0, 0.1% Tween20, 1× cOmplete Protease inhibitors (Sigma Aldrich, MO, USA)) and 2 glass beads (3 mm) were added to 50 mg feces, vortexed and sonicated in a water bath for 10 min. After homogenization, the sample was centrifuged for 15 min at 4 °C at 200× *g* and pellet and supernatant were kept. For further purification, this step was repeated three times. The supernatant of the first washing step and the pellet was used for proteomic analysis. To process the bacterial pellet fraction, glass beads were removed, and the pellet was resuspended in 20 mM Tris/HCl and then further diluted in lysis buffer (20 mM Tris/HCl, pH 7.5, 2% SDS, 1× cOmplete Protease inhibitors (Sigma Aldrich)). Samples were heated to 60 °C for 10 min and ultrasonicated for 3× 1 min (0.5 amplitude, Sonopuls Mini20, Bandelin) on ice. After lysis, samples were centrifuged for 1 h at 4 °C at 20,000× *g* and the supernatant was reduced with 10 mM dithiothreitol (DTT) at 50 °C, 700 rpm for 40 min and alkylated with 55 mM chloroacetic acid (CAA) at room temperature for 20 min in the dark. Samples were mixed with 1× lithium dodecyl sulfate (LDS) buffer and run into an sodium dodecyl sulfate (SDS) gel (5 min, 200 V). The gel was stained with Coomassie and one single stained sample band was cut for in-gel digestion following standard procedures [24]. For the supernatant fraction of the fecal washing, 50 µL of each sample supernatant was reduced, alkylated, denatured, and in-gel digested as described above. Input material for LC-MS/MS analysis was normalized based on feces weight.

### 2.3. LC-MS/MS Analysis

LC-MS/MS measurements were performed using a Dionex Ultimate 3000 UHPLC+ system coupled to a Q Exactive HF mass spectrometer (Thermo Fisher Scientific, MA, USA). After reconstitution in 0.1% formic acid (FA), one half of the peptides were loaded on a trap column (75 µm × 2 cm, packed in-house with 5 µm C18 resin; Reprosil PUR AQ, Dr. Maisch) and washed using 0.1% FA at a flow rate of 5 µL/min for 10 min. Subsequently peptides were transferred to an analytical column (75 µm × 45 cm, packed in-house with 3 µm C18 resin; Reprosil PUR AQ, Dr. Maisch) with a flow rate of 300 nL/min and separated using a 60 min gradient from 4 to 32% LC solvent B (0.1% FA, 5% DMSO in acetonitrile) in LC solvent A (0.1% FA, 5% DMSO) [25]. The instrument was operated in data-dependent acquisition (DDA) and positive ionization mode. MS1 full scans were acquired from 360 to 1300 *m*/*z* at a resolution of 60 K, an automatic gain control (AGC) target value of 3e6 charges and a maximum injection time (maxIT) of 10 ms. Precursor ions for higher energy collisional dissociation (HCD) fragmentation were selected with a Top 20 method, and MS2 spectra were recorded from 200 to 2000 *m*/*z* at 30 K resolution using an isolation window of 1.7 *m*/*z*, an AGC target value of 2e5 charges, a maxIT of 50 ms (for pellets) and 100 ms (for supernatants), 25% normalized collisional energy (NCE), a dynamic exclusion of 20 ms (for pellets) and 25 ms (for supernatant) and with a fixed first mass of 100 *m*/*z*. Samples were randomized for measurement, and *E. coli* standards were measured as quality control every 30 samples. As a quality control for lysis and digestion, *E. coli* samples were processed along with every 9 samples (Supplementary Figure S1).

### 2.4. Data Processing and Analysis

For proteomic data analysis, raw files were searched using Maxquant/Andromeda (v. 1.5.7.4) [26] against four different sequence databases: the Integrated Genome Reference Catalog (IGC): 9,878,647 entries [27], SWISS-PROT bacteria: 333,480 entries, downloaded 24 April 2018, SWISS-PROT human canonical and isoform: 42,123 entries, downloaded 2 January 2016, Sample specific databases based on metagenomic sequencing, see below). For the human database, all 424 raw files were searched together, whereas for the bacterial databases each sample (pellet and supernatant combined) was searched separately. MaxQuant results were post-processed for 1% peptide spectrum match (PSM) and peptide false discovery rate (FDR) using Percolator [28]. Taxonomic and functional annotation was obtained using the Unipept Metaproteome Analyzer tool (v. 4.0) [29,30]. The mass spectrometry proteomics data have been deposited to the ProteomeXchange Consortium (http://proteomecentral.proteomexchange.org) via the proteomics identifications (PRIDE) partner repository [31] with the dataset identifier PXD011515.

### 2.5. Metagenomic Sequencing and Data Processing

Part of the feces sample was used for the extraction of DNA to determine the microbiota composition. DNA was extracted using a QIAamp^®^ Fast DNA Stool Mini kit (QIAGEN) with a previous step of mechanical disruption to improve cell lysis. Briefly, cells were resuspended in 1.4 mL of Inhibitex buffer and 500 μL of 0.1 mm glass beads and tubes were vortexed at maximum speed for 5 min prior to the lysis at 95 °C for 7 min. Subsequent steps of the DNA extraction followed the QIAamp kit protocol. DNA concentration was determined with a QubitTM fluorometer using the manufacturer’s protocol. DNA was sequenced using the NextSeq platform from Illumina (high-output 300 cycle kit), following the manufacturer’s protocol. An average of 11 M reads per sample and an average 47× coverage was obtained. For processing, adapter sequences were removed from raw data using Cutadapt (v. 1.12) [32]. Quality filtering was performed using Trimmomatic (v. 0.38) [33]. Only reads with a size of 101 bp or higher were further processed to avoid possible misclassification of short reads. Cleaned genomic data (on average 3.1 Gb per sample) were assembled with SPAdes (v. 3.7.1) [34] using the ‘meta’ algorithm to improve the metagenomics reconstruction. To maximize the contig size and reduce the probability of chimeric contig reconstruction, the following k-mer lengths were selected: 21, 33, 55, 77, 121, 251. For each sample, contigs with greater than 2500 bp were selected. From those, open-reading frames (ORFs) were identified and annotated using Prodigal (v. 2.6.2) [35]. Contigs from all samples were combined and clustered at 99% identity and 90% coverage to remove redundancy in the dataset, using VSEARCH (v. 2.9.0) [36]. Trimmed reads were then mapped against the database of non-redundant contigs using Bowtie2 (v. 2.3.4) [37]. Contig frequencies were used to reconstruct metagenomic-assembled genomes (MAGs) using MetaBAT (v. 2.10) [38]. Only MAGs with a completeness of, at least, 70% and a contamination of less than 10% were kept. MAGs were classified phylogenetically using CheckM (v. 1.10.13) [39]. Phylogenetically coherent MAGs were merged together using CheckM if their completeness increased more than 10% and the merged contamination was lower than 10%. For protein database generation ORFs were translated into amino acids.

### 2.6. 16S rRNA Sequencing and Data Processing

Extracted DNA was further used to determine the taxonomic distribution of each fecal sample. Therefore, the V3-V4 region of the 16S rRNA gene was amplified and sequenced using the MiSeq platform from Illumina, as described in the manual for “16S Metagenomic Sequencing Library Preparation” of the MiSeq platform (Illumina). Briefly, for each sample, a 25 μL reaction was prepared containing 12.5 ng of DNA, 12.5 μL 2× KAPA HiFi Hot Start Mix, and 0.2 mM of primers. Water was added to complete the volume of the reaction. In case there was not enough amount of DNA, the maximum volume (11.5 μL of DNA) was added to the reaction, but the number of amplification cycles was increased from 25 to 30. Cycling conditions were 95 °C for 3 min, and 25 cycles of 95 °C for 30 s, 55 °C for 30 s, and 72 °C for 30 s, and a final elongation cycle at 72 °C for 5 min. The amplification was confirmed through electrophoresis by loading 4 μL of the PCR reaction on a 1.6% agarose gel. Subsequently, the PCR product was purified with the AMPure XP beads as described in the Illumina protocol. Next, a limited-cycle PCR reaction was performed to amplify the DNA and add index sequences on both ends of the DNA, thus, enabling dual-indexed sequencing of pooled libraries. Index PCR consisted of a 50 μL reaction containing 5 μL of the DNA obtained from the previous PCR, 25 μL of 2× KAPA HiFi Hot Start Mix, and 5 μL of forward and reverse indexed primers. Temperature conditions were the same as for the first reaction, but the number of cycles was reduced to 8. The obtained PCR product was purified with the AMPure XP beads following the manufacturer’s protocol. An equal amount of the purified DNA was taken from each sample for pooling. Each pool of samples (N = 96) was sequenced following Illumina recommendations. Sequences were processed using Mothur (v. 1.35) [40]. Initial trimming by quality was performed on paired ends of sequences before joining them into a single read. For this initial trimming the Prinseq Lute package was used. Parameters used for trimming included elimination of sequences shorter than 250 bp or that contained homopolymers longer than 8 bp or undetermined bases. Using the base quality scores, which range from 0 to 40 (0 being ambiguous base), sequences were trimmed using a sliding-window technique from the 3’ end, such that the minimum mean quality score over a window of 50 bases never dropped below 25. Sequences were aligned to the 16S rRNA gene using the SILVA reference alignment as a template. Potential chimeric sequences were removed using the Uchime algorithm. To minimize the effect of pyrosequencing errors in overestimating microbial diversity, rare abundance sequences that differ in up to four nucleotides from a high abundant sequence were merged to the high abundant sequence using the pre.cluster option in Mothur. Since different numbers of sequences per sample could lead to a different diversity (i.e., more Operational Taxonomic Units-OTUs could be obtained in those samples with higher coverage), we rarefied all samples to the number of sequences obtained in the sample with the lowest number of sequences (10,095). In other words, 10,095 sequences were randomly selected from each sample for subsequent analysis: taxonomic characterization and OTUs identification. Sequences with distance-based similarity of 97% or higher were grouped into the same OTU using the VSEARCH [36] abundance based greedy clustering method. OTU-based microbial diversity was estimated by calculating the Shannon diversity index [41]. Each sequence was classified using the Bayesian classifier algorithm with a 60% bootstrap cutoff [42]. In most cases, classification could be assigned to the genus level. When it was not possible to classify a sequence to a certain taxonomic level, it was assigned as “Unclassified” followed by the upper taxonomic level.

### 2.7. Determination of 16S rRNA Counts with qPCR

To determine the total bacterial load of each fecal sample, qPCR of the 16S rRNA gene was performed. For this purpose, the KAPA SYBR FAST qPCR Kit was used. Briefly, for each sample, 20 μL PCR duplicates were prepared with each containing 2 μL of the DNA (see previous section) used as template, 10 μL of mix provided by the manufacturer, and 0.4 μL of forward and reverse primers at the final concentration of 0.2 mM. To complete the volume of the reaction, 7.2 μL of water was added. A PCR product of the 16S rRNA gene from *Enterococcus faecium* C68 strain was used for obtaining a standard curve. ENDMEMO program was used to determine the number of 16S rDNA molecules in the PCR product of *E. faecium* C68 based on sequence of 16S rRNA gene and concentration of the PCR product. A standard curve was obtained by making 5-fold dilutions of the PCR product. Cycling conditions of the qPCR were 94 °C for 5 min, and 45 cycles of 94 °C for 30 s, 56 °C for 30 s, and 68 °C for 30 s, and a final elongation cycle at 68 °C for 5 min. By extrapolation of the obtained results with the standard curve, the number of 16S rRNA gene copies was determined for each sample.

## 3. Results and Discussion

### 3.1. The Challenge of Experimental Design in Clinical Metaproteomics

**Figure 1:**
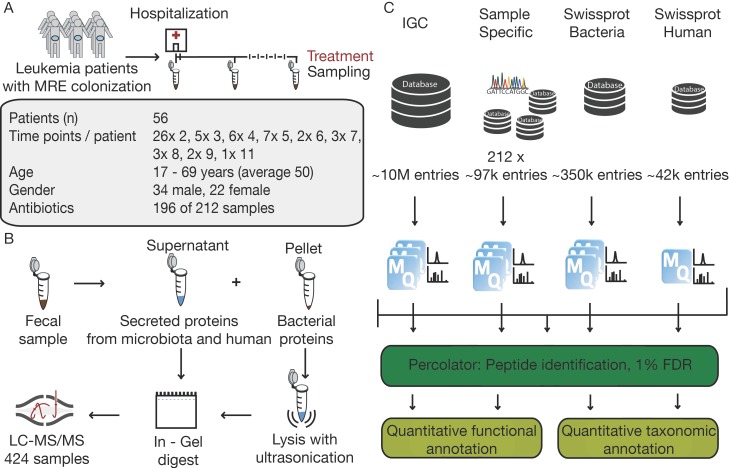
Study design, proteomic workflow and data processing pipeline. (**A**) Acute Leukemia patients were sampled in weekly interval during the time of hospitalization. In total 212 fecal samples of 56 patients with MRE gut colonization were analyzed, providing additional information about age, gender, and treatment conditions. (**B**) For the protein extraction, fecal samples were divided in supernatant and pellet fractions. Bacterial cells in the pellet fraction were lysed with ultrasonication and for both samples’ proteins were digested in gel. Thereafter, samples were measured with LC-MS/MS. (**C**) Raw files were searched with four different databases in separate and combined MaxQuant searches and post-processed with Percolator and with quantitative functional and taxonomic annotation analyzed.

In total, 212 fecal samples from 56 acute leukemia patients with MRE gut colonization were processed for metaproteomic analysis. Samples had been collected during hospitalization in approximately one-week intervals (Figure 1A). The cohort consisted of patients suffering from acute leukemia who were treated with chemotherapy or undergoing transplantation in addition to receiving antibiotics to treat their infections (Supplementary Table S1). As evident from Supplementary Table S1, the level of experimental control is much lower and the issue of confounding factors much higher in clinical studies than those conducted in animal or other model systems. Collecting multiple samples per patient and a large overall sample size are necessary to estimate inter- and intra-patient variability and to ensure statistical power.

The analytical workflow, however, from sample preparation to metaproteomic data is much easier to control. Here, samples were analyzed using a standardized proteomic workflow. Samples were divided into a supernatant and a pellet fraction to separate bacterial cells from secreted proteins of human and microbial origin. We opted for a sample preparation step that included gel electrophoresis and using in-gel trypsin digestion to generate peptides for LC-MS/MS analysis. In our experience, a short SDS gel electrophoresis step is a convenient and reproducible ‘sample equalizer’ because it is compatible with harsh upstream protein extraction procedures (see methods) and generates peptides that are essentially devoid of non-peptidic contaminations, detergents, and insoluble particles, etc. (Figure 1B, Supplementary Figure S1). The employed ‘single-shot’ LC-MS/MS measurement strategy is also highly reproducible ensuring that the vast majority of the observed variation in the project data arises from biological rather than technical sources. The raw MS data were processed using four sequence databases of different sizes and content covering human and bacterial proteins. In this way, we were able to compare peptide identification results of every single database to the combination of all (Figure 1C). For the bacterial databases, SWISS-PROT annotated entries for bacteria and the large integrated reference gut microbiome catalog (IGC), both publicly available resources, were used. In addition, we performed shotgun metagenome sequencing for every sample and generated sample specific protein sequence databases for each.

### 3.2. The Challenge of (the Lack of) a Comprehensive Sequence Search Space

**Figure 2:**
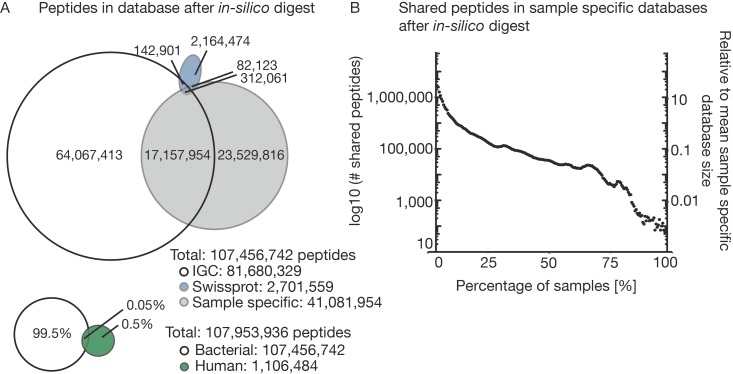
*In silico* comparison of four different databases. Four different databases (Integrated Genome Reference Catalog (IGC), SWISS-PROT bacteria, SWISS-PROT human and sample specific metagenome-based databases) were digested *in silico,* and the possible search space was compared. (**A**) Venn diagram of the resulting peptides after *in silico* digestion comparing the three bacterial databases and all bacterial databases combined versus the peptides from the *in silico* digested human database. (**B**) Number of shared peptides in the 212 sample specific databases against the percentage of samples. The right axis indicates to which the percentage of the average sample specific database the number of shared peptides corresponds.

To be able to convert LC-MS/MS data into identified peptide sequences, comprehensive, ideally complete, protein sequence collections must be available for the (many) species that are present in a metaproteomic sample. The selection of the sequence database is, therefore, a particularly important step for metaproteomic analyses [21,43]. To demonstrate this challenge, the following section investigates several theoretical aspects before we turn to the experimental data. To illustrate the size of the theoretical search space for the three bacterial and the human sequence databases used in this study, we digested each in silico to compare the number of resulting theoretical tryptic peptides. With almost 10 million protein entries, IGC is the largest database in our comparison. It was assembled as part of an international initiative that performed shotgun metagenome sequencing of 1070 individuals from around the world and is considered to be a comprehensive and high-quality gene catalog for the human gut microbiome. Somewhat surprisingly, it turns out that IGC only covers half of the theoretical peptides derived from the sample-specific sequencing databases (Figure 2A) generated in our project. This means that even IGC is not nearly as comprehensive as one might expect. Again, in proteomics, peptide and, therefore, protein identification relies on the matching of spectra to peptide sequences from the protein database. If a peptide sequence in the database does not fit the actual sequence of the acquired spectrum, it cannot be identified. Even single amino acid changes can lead to missing and false positive identifications. Therefore, it is important that the protein database used for peptide identification matches the bacterial composition of a given sample as closely as possible [44]. Bacterial populations are evolving very fast to adapt to changing environments [45]. Therefore, each individual will likely contain unique strain compositions in the gut although particular bacterial species can be shared [46]. It is, therefore, not clear if or to what extent public metagenome collections can ever be comprehensive.

In comparison, the catalog of bacterial proteins in SWISS-PROT is much smaller, but its entries are manually validated and annotated which is why SWISS-PROT is perhaps the highest quality sequence database available. However, the vast majority of the bacterial entries in SWISS-PROT are not specific to the gut environment and, thus, one might not expect a major overlap to the genomic databases. Indeed, SWISS-PROT shares only 0.5% of the total peptides with either IGC or the sample-specific protein databases and is, therefore, not a useful source of sequences for gut metaproteomics.

By direct comparison, the bacterial databases combined are 200 times larger than the theoretical search space of human sequences in SWISS-PROT. Fortunately, only 10% of all theoretical human peptides are shared with the bacterial database entries allowing the identification of bacterial and human peptides in a sample in parallel. Comparing the 212 individual sample specific databases to each other (Figure 2B) shows that the individual databases are very diverse. Only around 100,000 peptides are shared between at least 50% of the samples. This corresponds to just 12% of the median size for each sample specific database. This illustrates that even with the sensitivity and comprehensiveness of genome sequencing, a large proportion of the proteins in a sample are unique or contain amino acid variations, demonstrating very considerable diversity of the gut microbiota between and across individuals. This, in turn, severely limits the extent to which quantitative comparisons can be made on the level of individual proteins between patients, their colonization status, medication or other metadata.

### 3.3. The Challenge of Extensive Demand for Computational Power and Storage Capacity

In moving from theoretical consideration to the analysis of the experimental data, we performed MaxQuant searches for all samples and all four databases separately. As the false discovery rate (FDR) estimation procedure of MaxQuant shows strong limitations when very large sequence databases are used (Supplementary Figure S2), MaxQuant outputs were post-processed using Percolator to recover identifications. For the combination of database searches (‘Combined’), all MaxQuant results of the individual searches against IGC, SWISS-PROT bacteria and the particular Sample specific database were combined but only retaining the highest scoring peptide sequence per spectrum. The combined MaxQuant results were then post-processed in Percolator. Searching the IGC sequence database (~10 million entries) required considerable computational power and time. For one single sample, the search against IGC produced an output of 85 GB on average. In contrast, searching the much smaller sample-specific databases generated an output of 4.5 GB on average per sample. One theoretical advantage of searching IGC using MaxQuant is that all samples of a project can be combined into a single run, which improves the grouping of protein sequences into a minimal set. However, such a search was not practical given the 2 × 212 LC-MS/MS files produced in this study. Searching all samples against IGC (the pair of pellet and supernatant for each sample were searched together), produced an output of 17.8 TB of files and required ~1834 h of run time (10 cores, Intel^®^ Xeon^®^ Gold 6150 CPU @ 2.70GHz (4 processors)). In contrast, searching all 212 sample specific databases produced an output of ‘just’ 830 GB in ~310 h of run time (2 cores, Intel^®^ Xeon^®^ Gold 6150 CPU @ 2.70GHz (4 processors)). This illustrates that searching IGC is not practical in most instances, particularly given the often limited computational infrastructure available in hospitals. And even searching the sample-specific databases will pose challenges when analyzing large sample cohorts.

**Figure 3:**
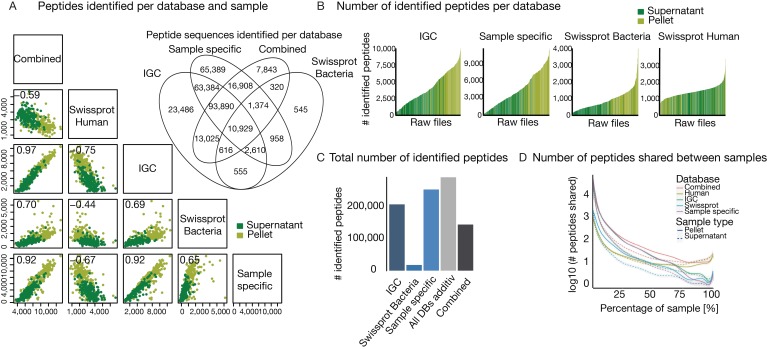
Comparing the influence of database selection on peptide identification. (**A**) Multi-scatter plot of identified peptides at 1% PSM and peptide FDR for the four different databases and all databases combined. Identification for pellet and supernatant fraction of each sample is shown separately. Pearson correlation is shown in top left of each box (highest p value is 8.2 × 10^−21^). The Venn diagram shows the overlap of identified peptides over all samples for the three bacterial and the combination of all four databases. (**B**) Histogram of the number of identified peptides of supernatant or pellet for each sample. Raw files are sorted according to the number of identified peptides. (**C**) Bar plot of the number of total identified peptides over all samples per database. ‘All DBs additive’ shows the theoretical identification by summing up all unique peptides of the three bacterial database types. (**D**) Polynomial curve fit for the number of shared peptides across all samples for the different databases. Separated for supernatant and pellet fraction of the samples.

The total number of identified peptides across all databases showed considerable variation ranging from just a few hundred to up to 10,000. To check for biases in identifications, we compared peptide identification between supernatant and pellet fraction of each sample, Shannon-diversity [41], 16S rRNA count and Bradford protein concentration with the number of identified peptides of each sample (Supplementary Figure S3, Supplementary Table S2). As expected, good correlation for peptide identification between pellet and supernatant was observed, whereas the other parameters showed low correlation. The Venn diagram of shared identified peptide sequences for all bacterial databases shows that most peptides are covered by the sample-specific databases (Figure 3A, top right inset). High positive correlations can be observed when comparing the number of identified peptides between the bacterial databases. Only for SWISS-PROT, the correlations are slightly lower, which probably arises from the overall lower number of identifications. Negative correlations were obtained for comparison to the human database (Figure 3A), i.e., the more bacterial peptides were identified, the fewer human peptides could be found. This is readily explained by the fact that the mass spectrometer cannot exhaustively measure all peptides in a sample and the detection of (largely constant) human background proteome is increasingly suppressed the more bacteria are in a sample. As one would expect, more peptides were identified in pellet samples than in the corresponding supernatants (Figure 3B). In addition to expectation, peptides of human origin were more frequently identified in the supernatant. The numerical range of identified peptides from the human database search was smaller compared to the bacterial peptides, again indicating that there is a largely constant background of human proteins in the samples.

Comparing the total number of peptide identifications showed that sample specific databases yielded the highest number of identification, followed by the IGC (Figure 3C). This underscores that sample specific databases are more representative of metaproteomic content than IGC as already expected from the theoretical considerations discussed above. In addition, we observed that combining all bacterial databases (‘Combined’, including IGC, Sample specifics, and SWISS-PROT Bacteria) for post-processing into one leads to a substantial decrease in peptide identifications. As noted above, this is a direct consequence of the limitations of current database search engines in controlling peptide identification FDR when using large sequence collections [47]. Unfortunately, no fully satisfactory practical solution has been found to this issue yet. When combining all unique peptide sequence identifications of the three bacterial databases (‘all DBs additive’) identifications would be increased by ~20%. However, this is not a very practical approach because of the demands on computational power also discussed above and limited FDR estimation accuracy.

Any comparative analysis requires that the same peptides/proteins are found between samples. Figure 3B highlights the large range of peptide identifications per sample. When we calculated the number of identified peptides that are shared across samples, an extremely small overlap was observed (Figure 3D). The extent of this non-overlap is stunning as there were only very few peptides that were identified in all samples and most of these were consistently identified from the human host. This has profound implications that set metaproteomics apart from any single species proteomic investigation in that some type of heuristics must be applied to aggregate results into a level which allows comparison. This may be as high as the species level which would preclude any functional interpretation of the molecular level beyond what, for example, gene ontology (GO) analysis may have to offer.

From the above analysis, we conclude that sample-specific sequence databases are the preferred option for metaproteomic projects today. Therefore, all the analysis presented below is based on results from sample specific database searches.

### 3.4. The Challenge of Functional and Taxonomic Annotation

**Figure 4:**
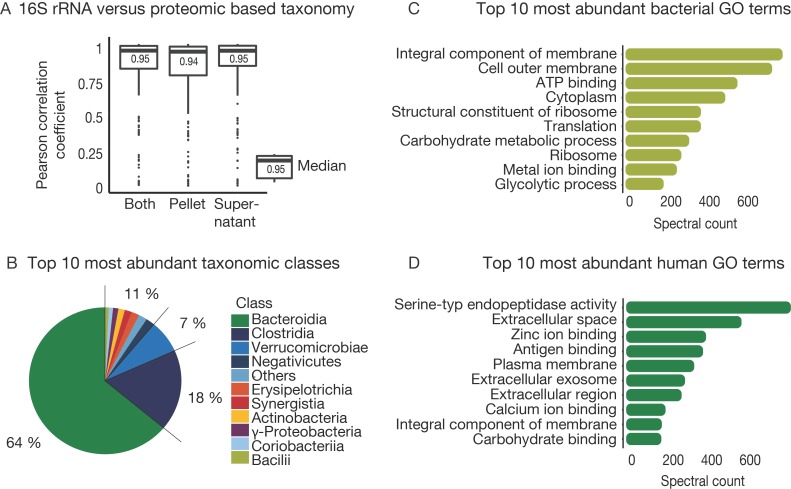
Description of the taxonomic and functional composition. (**A**) Box plot of Pearson correlation of taxonomic composition detected at the class level with 16S rRNA sequencing and proteomic analysis for each sample. Both: supernatant and pellet for each sample combined, Supernatant: only the supernatant fraction of each sample, Pellet: only the pellet fraction of each sample. (**B**) Pie chart shows the most abundant identified taxonomic classes over all samples. (**C**) Bar plot of average spectral counts for the 10 most abundant bacterial gene ontology (GO) term over all samples. (**D**) Bar plot of average spectral counts for the 10 most abundant human GO term over all samples.

Although metagenome-derived sequence databases are currently the best available representation of the possible species and protein content of a sample, subsequent annotation by mapping peptide or proteins to taxonomic and functional information is required. For this purpose, we used the Unipept metaproteome analyzer tool for the annotation of tryptic peptides. In addition to metagenome and metaproteome data, we also generated 16S rRNA gene sequencing data for all samples (Supplementary Table S3). To compare class-level 16S rRNA information to the proteomic data, peptides were mapped to their lowest common ancestor. For proteomics, it was possible to map 60% of the identified peptides to a taxonomy level. The remaining peptides could not be found in the Unipept database (NCBI) or were not unique for taxonomic identification. In that way, we identified 72 classes, 383 genera, and 595 species in the patient samples (Supplementary Table S4). In comparison, 16 S rRNA data identified 32 classes and 334 genera across all samples. Gratifyingly, the overall taxonomic distribution per sample derived from the proteomic and 16S rRNA data showed a mean Pearson correlation of 0.95 at the class level (0.94 for order, 0.78 for family, and 0.87 for genus), meaning that it was possible to derive taxonomic distributions from metaproteomic analysis, and there was no evidence that poor correlations generally resulted from poor proteome coverage (Figure 4A, Supplementary Figure S4). In line with prior results [48,49], the most abundant classes over all samples were Bacteroidia, Clostridia, and Verrucomicrobiae (Figure 4B).

For further functional annotation, 80% of the peptides mapped to at least one GO term, and 42% could be associated to an E.C. number. This indicates the detection of proteins related to 1,205 molecular functions, 738 biological processes, and 145 cellular components for bacterial peptides and 298 molecular functions, 337 biological processes, and 139 cellular components for human peptides. The ten most abundant GO in terms of the microbiota over all samples mainly represented cell growth and metabolic homeostasis (Figure 4C). In contrast, abundant human peptides were mainly associated with immune responses and metabolic/catabolic activity and belong to proteins that are located in extracellular regions (Figure 4D), which largely recapitulates observations reported by earlier studies of host-microbiome interactions [6,50]. One important learning from this section is that metaproteomics can represent the taxonomic diversity in a sample as well as 16S rRNA gene sequencing but simultaneously providing more details on functional molecular information.

### 3.5. The Challenge of High Microbiome Variability in Clinical Samples

**Figure 5:**
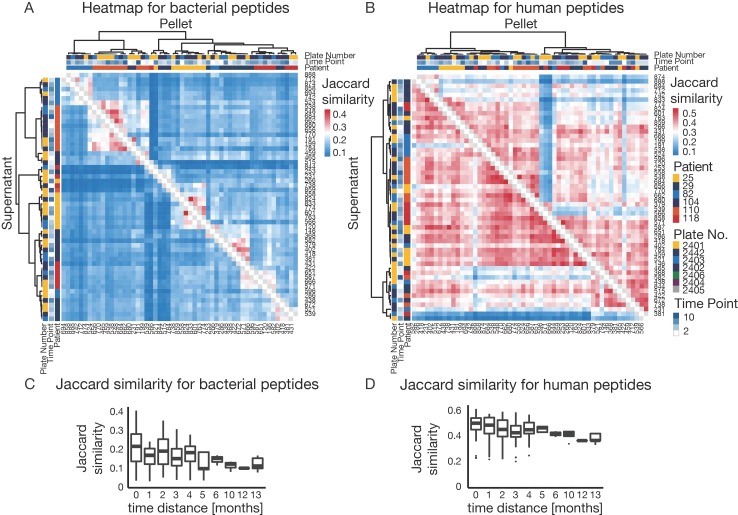
Sample variability (**A**) Heatmap of Jaccard similarities based on the presence/absence of bacterial peptides for the top six patients with the most sampling time points. Dendrogram clustering is based on Pearson correlation of Jaccard distances. Bottom triangle for the supernatant fraction of the sample. Top triangle for pellet fraction of the sample. (**B**) Heatmap of Jaccard similarities based on the presence/absence of human peptides for the top six patients with the most sampling time points. Dendrogram clustering is based on Pearson correlation of Jaccard distances. Bottom triangle for the supernatant fraction of the sample. Top triangle for pellet fraction of the sample. (**C**) Boxplot of Jaccard similarities for bacterial peptides of paired samples with different time distances between sampling points. (**D**) Boxplot of Jaccard similarities for human peptides of paired samples with different time distances between sampling points.

The data shown in Figure 3 already indicated that there is a very high degree of variability in the metaproteomes of clinical samples, and the quality control standards within the proteomic workflow show that this is not the result of technical variations (Supplementary Figure S1). Therefore, simple comparisons between samples based on peptide and protein intensities, as are common for single species studies, were not possible. Instead, we resorted to using Jaccard similarity (Figure 5) to compare protein expression profiles of samples. Here, similarity is described by the presence and absence of peptides between pairs of samples. Clustering of dendrograms was based on Pearson correlation of Jaccard distances (see methods for details). Clustering of all samples (Supplementary Figure S5) showed a clear separation of supernatant and pellet samples. In addition, it was apparent that supernatant samples showed higher overall similarities than pellet samples because human proteins were more consistently identified in supernatants. We next selected six patients for whom the most multiple samples were available. The heat map shown in Figure 5A shows that the microbiomes within patients were more similar than between patients (Figure 5A). This indicates that peptide variation among samples is mainly driven by individual microbiome differences rather than by technical variation of the proteomic analysis. For peptides derived from human proteins, the overall similarity was expectedly much higher than for the microbiomes (Figure 5B). Patients did not, however, cluster based on the human peptide expression, indicating that the human expression profiles were much less variable.

**Figure 6:**
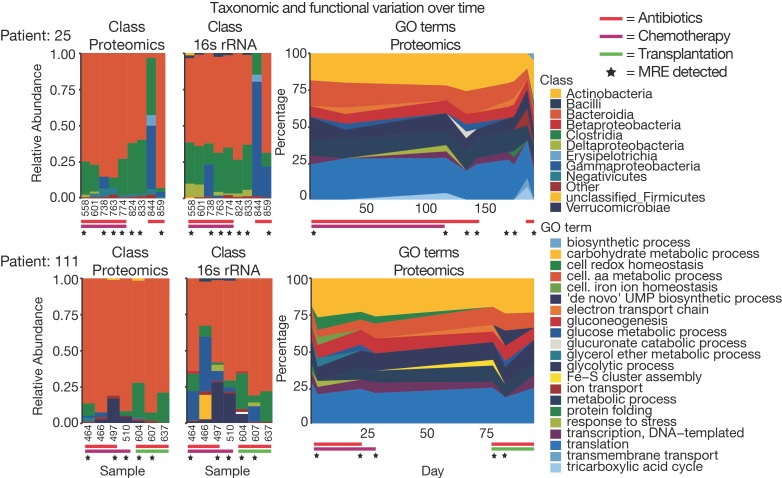
Comparing taxonomic and functional data for longitudinal samples. Taxonomic class abundances retrieved from proteomic and 16S rRNA data as well as GO term abundances were compared for samples for two patients over time. In addition, antibiotic treatment at sampling time point and type of hospital admission (i.e., chemotherapy or transplantation) for the sampling time is indicated.

The longitudinal data collected for several patients also allowed us to assess how their metaproteome changed over time. Figure 5C,D depict the grouped Jaccard pair-wise similarities for bacterial and human peptides based on the time between sampling. It is apparent that with increasing time, microbiome similarity decreased from 0.35 to below 0.2, whereas the human profile showed a more stable similarity of 0.5 to 0.4. This observation has important consequences for the future design of clinical metaproteomics projects as it seems important to collect a significant number of longitudinal samples, which we expect to improve the interpretability of individual metaproteomes. Nevertheless, it should be mentioned that patients in this study received a variety of antibiotics altering microbiome composition and, therefore, higher variability compared to a healthy population is expected [51]. To overcome the problem of missing values, analyses are often performed using functional or taxonomic annotation levels, where several peptides/proteins are combined and, therefore, variability is decreased (Supplementary Figure S6) [52]. In this way, the taxonomic and functional composition of patient samples can be compared quantitatively (Figure 6). Taxonomic and GO term distribution of samples for every patient can be found in the Supplementary Material 1.

## 4. Conclusions

Metaproteomics is a young and developing field of research. PubMed currently (20th October 2018) lists a total of ~500 publications, whereas ~7500 scientific reports are published per year in proteomics as a whole (Supplementary Figure S7). Although metaproteomic analysis has seen substantial progress, major challenges remain to be overcome. When designing future clinical metaproteomic studies, our data suggest that it is advisable to include longitudinal sampling systematically for each patient and to keep sampling intervals short and consistent within the cohort. Clinical studies often suffer from small sample sizes and, therefore, poor statistical power [53]. We emphasize this point for future clinical study designs as generating the actual metaproteomic data is no longer a bottleneck. As in all clinical studies, it is important to record as much (and correct) meta information as possible about the patients and their treatments to be able to account for potential confounding factors and to distinguish interesting effects from uncontrolled factors.

For the time being, the generation of sample-specific databases is highly recommended to support comprehensive peptide and protein identification. While this requires metagenome sequencing of each sample, it mitigates the inevitable loss in confident peptide identifications when using community-based resources, such as IGC. In addition, we propose that transcriptomics data from RNA-Seq could be another way to generate databases and would likely assess even better the contribution of individual species and protein to the overall protein expression. These approaches do, however, come at the price of having to generate metagenomes for each sample and to process each of these into a list of protein sequences. That may not always be feasible in terms of cost and time, in which case, IGC is the next best alternative. However, large database sizes come with several issues. First, the ability to distinguish correct from incorrect matches is strongly impaired. The concept of FDR estimation as defined by Elias and Gygi [54] comes with the assumption that the database is a comprehensive representation of the real search space. This assumption is, most of the time, not justified in metaproteomic samples. One option to overcome such artificial loss of identifications is to include semi-supervised machine learning algorithms like Percolator [28] or Nokoi [55] for the PSM scoring. Another approach to circumvent the issue of large search spaces is the clustering of peptides [56,57] or the use of 2-step searches like proposed by Jagtap et al. [58]. However, some controversy exists in the field of metaproteomics as to what degree the latter method leads to an under-estimation of the true FDR. To solve this problem for metaproteomics, a major rethinking of peptide match scoring is necessary. We anticipate that substantial progress will be made when using synthetic peptides as a ground truth for training predictors of tandem mass spectra. Large collections of synthetic peptides are becoming available by initiatives, such as the ProteomeTools project [59]. The use of sample-specific sequence databases for peptide identification also controls, at least to some degree, demand for large computational power and storage capacity.

Another obvious challenge of metaproteomics is sample variability and the high proportion of missing values which impairs the use of many statistical methods that require complete data matrices. Due to their high species complexity, metaproteomic samples show generally high variability. Sample variability could be enhanced in the present patient cohort due to the administration of chemotherapy, increasing mutational load in bacteria [60], and the administration of antibiotics, altering the intestinal microbiome composition [51]. This variability may be attenuated by increasing the dynamic range of the analytical workflow, e. g., using deep fractionation of peptides prior to LC-MS/MS analysis or depletion of non-bacterial contaminants. This would, however, imply increased time requirements and cost, and the production of an even higher ‘data mountain’. In addition, feasibility may not always be ensured especially for large clinical studies and low sample availability [61]. In contrast to mainstream proteomics, which makes strong use of intensity-based abundance estimation, metaproteomics is still largely confined to spectral counting methods because only few peptides are detected in many samples, which limits the accuracy with which changes can be measured [62]. In addition, most quantification methods require robust normalization of the data. In microbiology, samples are regularly normalized on the sample input weight (here feces). This may or may not be a fair representation of actual bacterial/protein amount/variation in a sample. It has become standard procedure in proteomics to normalize input material for LC-MS/MS measurements on the basis of total peptide or protein content to ensure equal depth of analysis and reproducible identification [63]. This may not be possible in metaproteomics: Comparability and normalization may be compromised because the feces may contain proteineous material other than from bacteria and host contributions. Many researcher are turning attention to data-independent-acquisition (DIA) strategies because it promises to improve reproducibility and quantification and could decrease the level of missing values. Yet, spectrum annotation and availability of suitable spectral libraries for DIA is still challenging for single proteomes and, in our view, the concepts and tools need to be much improved before DIA is applicable for complex metaproteomic samples.

An entirely different option to circumvent missing values on peptide/protein level is to compare abundances of GO terms and taxonomic distributions. Although, this shows promising results, clear taxonomic and functional annotation is not always feasible in metaproteomics. Because peptides can be shared between different proteins of the same organism or between multiple organisms, the protein inference problem in metaproteomic is even more pronounced than in single organism proteomics [21]. Unequivocal annotation to one species is, therefore, often not possible. To circumvent this problem, peptides and proteins are often mapped to the lowest common ancestor (LCA) as first described by Huson DH et al. [64] However, this clearly results in loss of information and potentially ambiguous annotations, limiting its applicability to higher phylogenetic levels, such as classes or phyla. Still, it is a very practical approach that does provide functional annotation and, thus, helps in the interpretation of metaproteomic data. This was strongly facilitated by the recent extension of the frequently used Unipept metaproteome analyzer to not only map the LCA on peptide level but also annotates peptides with GO terms and E.C. numbers. This functionality offers an alternative to other commonly used protein-based tools, such as Megan (Metagenome Analyzer) [65], eggNOG (evolutionary genealogy of genes: Non-supervised Orthologous Groups) [66] and KEGG (Kyoto Encyclopedia of Genes and Genomes) [67]. Of note, since Unipept works at the peptide level, it simplifies data analysis by side-stepping the need of BLAST searches, especially for sample-specific genomic databases but it needs to be mentioned that, to our knowledge, no validation study comparing peptide vs. protein level based annotation has been published. Despite this, both are frequently used in metaproteomics, and there is no consensus opinion on this point in the field of metaproteomics.

This report describes the taxonomic composition and functional process of patients during the MRE gut colonization progress. Further improvements in data analysis strategies and study designs are needed to explore the processes and interactions in the microbiome and the host in more detail. Elucidating the mechanism of microbiome provided colonization resistance against multidrug-resistant pathogens (e.g., bacteriocins), the microbiome influence in disease development following transplantation (e.g., graft versus host disease) or chemotherapy efficacy. We are confident that with new technology and software, most of the challenges will eventually be solved, enabling future studies to move from merely describing taxonomic and functional composition changes to revealing significant protein-centric molecular and functional processes.

## Supplementary Materials

The following are available online at http://www.mdpi.com/2227-7382/7/1/2/s1, Figure S1: Quality control with *E. coli* standard, Figure S2: Database results with MaxQuant versus MaxQuant + Percolator, Figure S3: Comparing peptide identification for sample fractions, database size, protein concentration, 16S rRNA count and diversity, Figure S4: Correlation of taxonomic distribution between 16S rRNA and proteomic data, Figure S5: Expression profile for all samples, Figure S6: Decreasing level of annotation coverage during data analysis process, Figure S7: Publications in the metaproteomic and proteomic field, Table S1: Metadata for patient samples, Table S2: 16S rRNA count and protein concentrations, Table S3: 16S rRNA taxonomic composition, Table S4: Taxonomic and functional annotation of peptides, Material 1: Taxonomic and GO term abundances for patients over time.
