# Supplementary material for: Challenges in Clinical Metaproteomics Highlighted by the Analysis of Acute Leukemia Patients with Gut Colonization by Multidrug-Resistant Enterobacteriaceae
Source: Proteomes. 2019 Jan 8;7(1):2. doi: 10.3390/proteomes7010002 (PMC6473847; doi:10.3390/proteomes7010002)
Supplement: Supplementary file 1 [file proteomes-07-00002-s001.zip › supplement/Supplementary_Figures.pdf]

Supplementary Figures

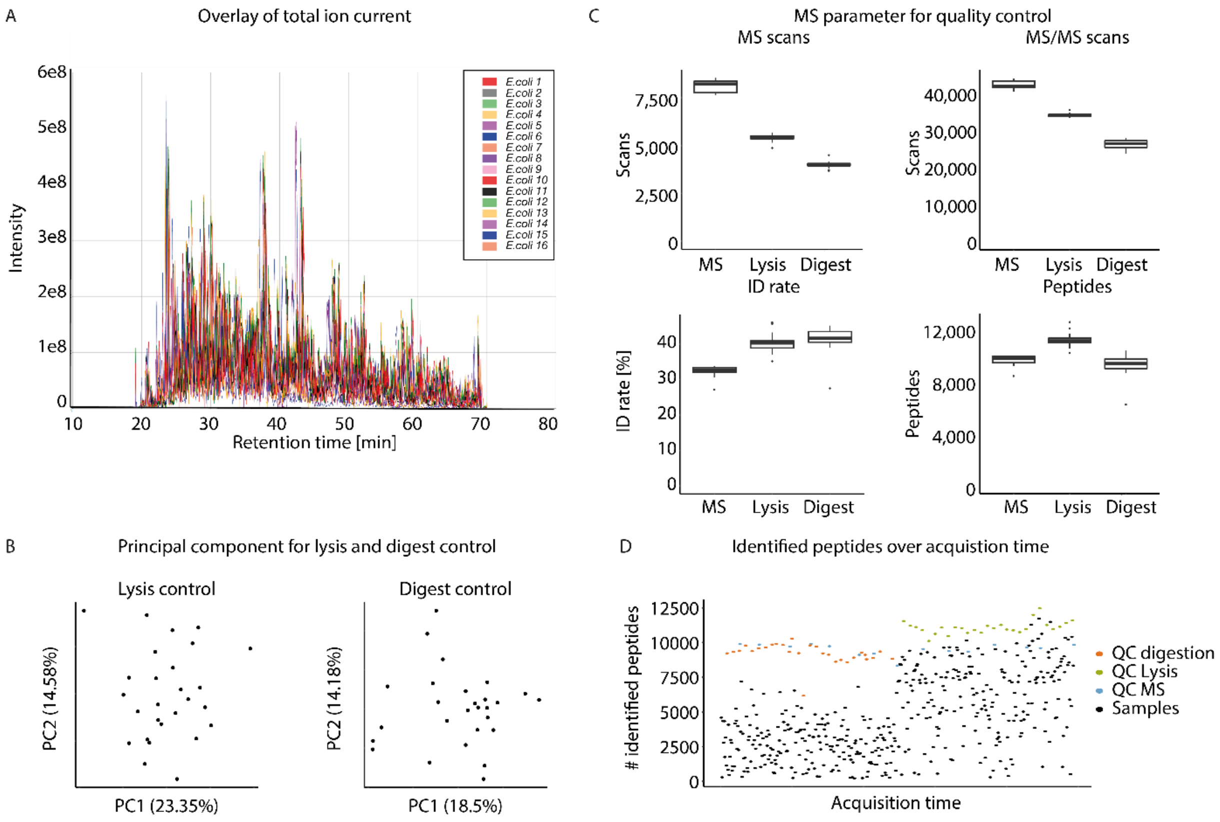

Supplementary Figure S1: Quality control with *E. coli* standard. (a) Overlay of the TIC intensity for the measured *E.coli* standards during measurement of all samples. (b) PCA plot for the protein intensities in the *E. coli* quality standards for control of lysis and in-gel digest sample preparation. (c) Box plots show the number of acquired MS1 scans, MS2 scans, spectrum identification rate and number of identified peptides for the MS, lysis and in-gel digest control *E.coli* standards. (d) Number of unique peptides identified in each sample and each quality control *E.coli* standard over acquisition time.

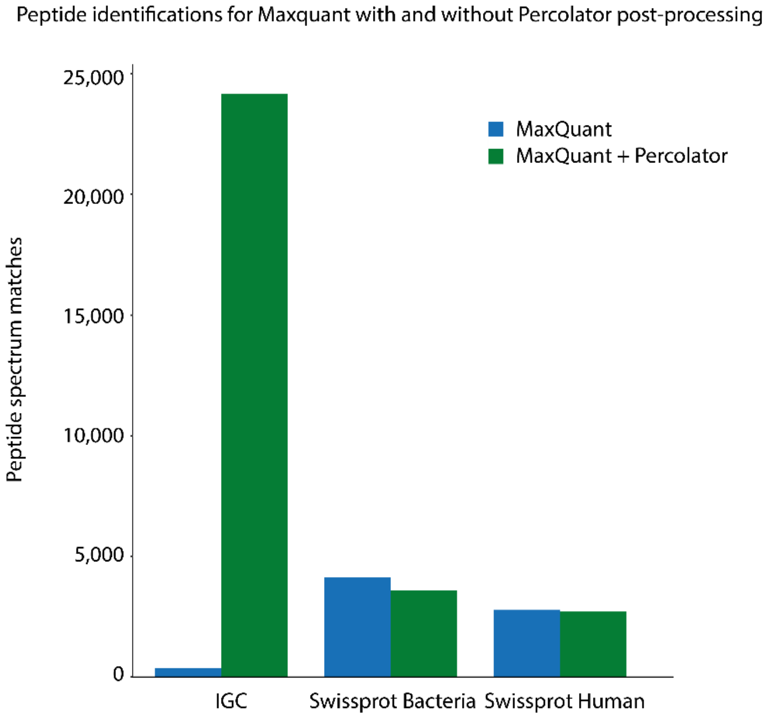

Supplementary Figure S2: Database results with MaxQuant versus MaxQuant + Percolator. Bar plot showing the number of identified peptide spectrum matches for eight raw files of the study (covering high and low complex samples) for MaxQuant search engine Andromeda with and without combination of Percolator comparing three different database sizes.

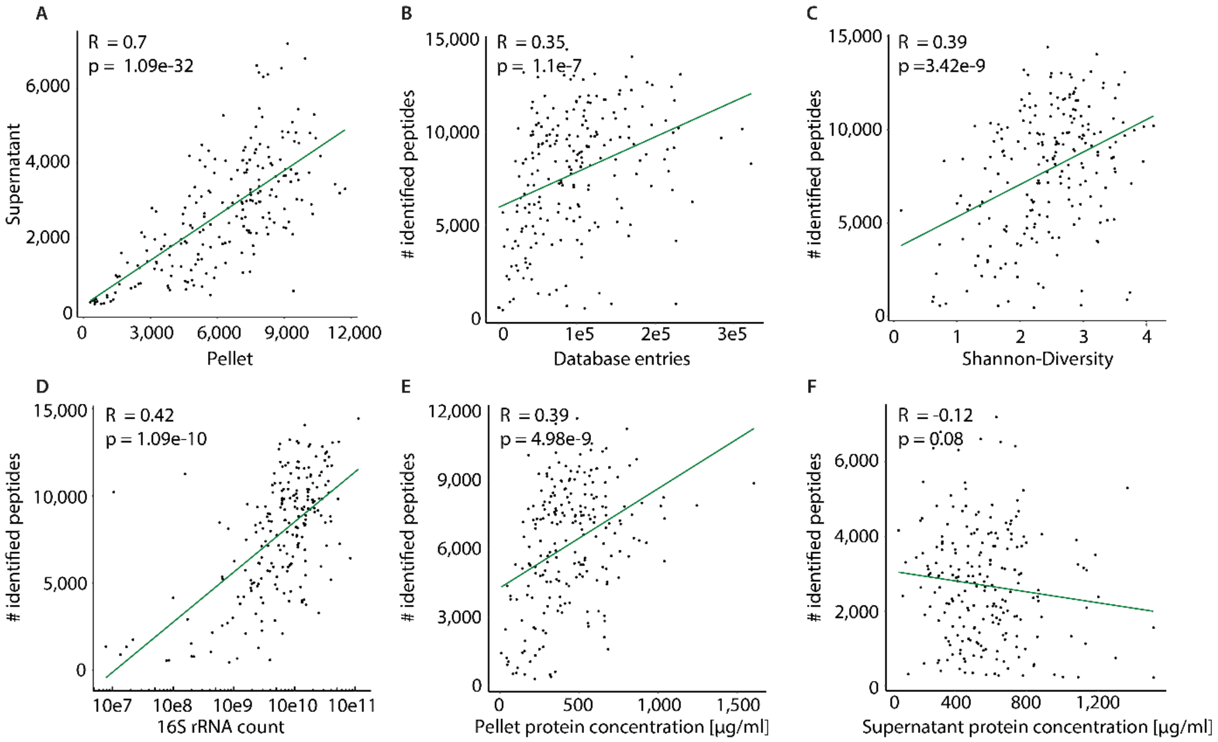

Supplementary Figure S3: Comparing peptide identification for sample fractions, database size, protein concentration, total viable count and diversity. (a) Scatter plot showing the number of identified peptides with sample specific databases for supernatant and pellet fraction. (b) – (f) Scatter plot showing the number of identified peptides with sample specific databases for pellet and supernatant fraction combined per sample (top left) or for both fractions combined against sample specific database size, 16s rRNA based microbial Shannon diversity [Shannon & Weaver, 1949], 16S rRNA count and Bradford protein concentrations during sample preparation.

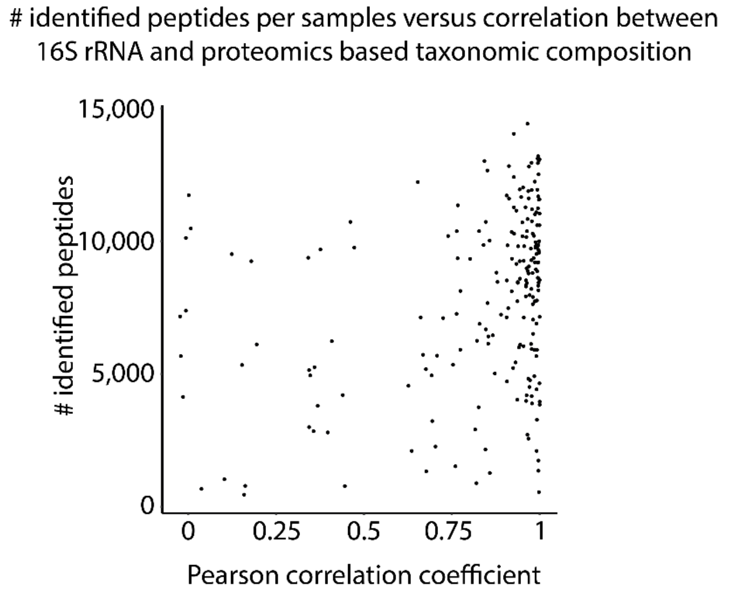

Supplementary Figure S4: Correlation of taxonomic distribution between 16S rRNA and proteomic data. Pearson correlation for taxonomic distribution based on 16S rRNA gene sequencing and metaproteomic analysis is plotted against the number of identified peptides per sample.

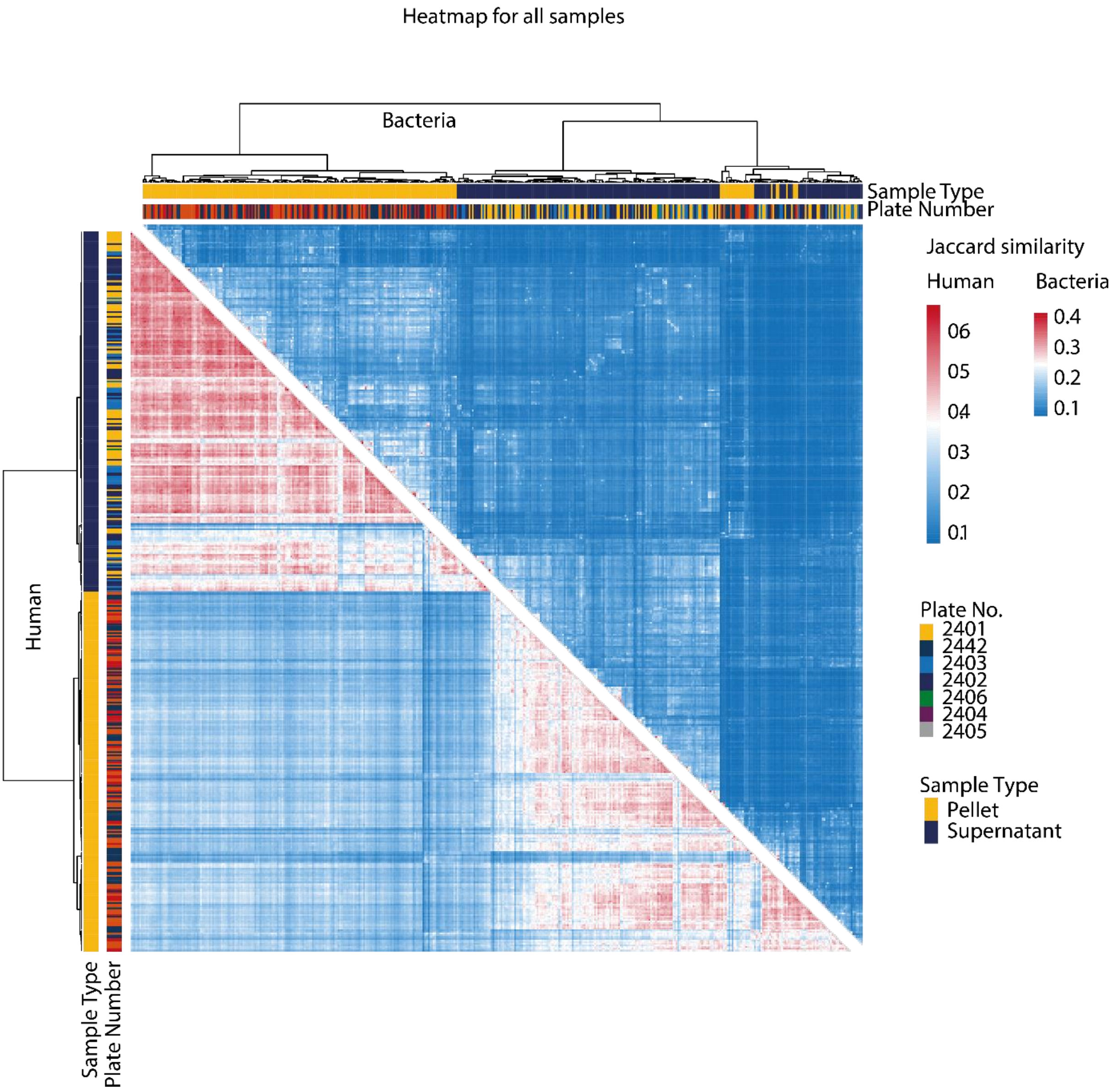

Supplementary Figure S5: Expression profile for all samples. Heatmap shows pair-wise comparison of all samples (separate for pellet and supernatant fraction) based on Jaccard similarities and clustering is based on Pearson correlation of Jaccard distances. Samples are annotated by the 'Sample Type' (pellet or supernatant fraction) and 'Plate No.' (Identifier for the 96 well plate during MS measurement time, represents acquisition order).

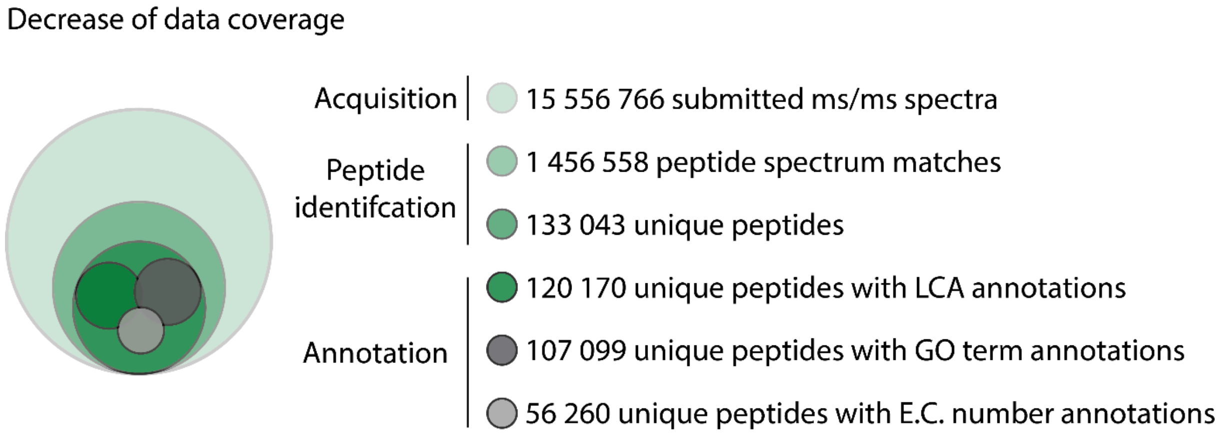

Supplementary Figure S6: Decreasing level of annotation coverage during data analysis process. Schematic overview of the decreasing numbers from acquired MS/MS spectra to the level of functionally and taxonomic annotated peptide sequences.

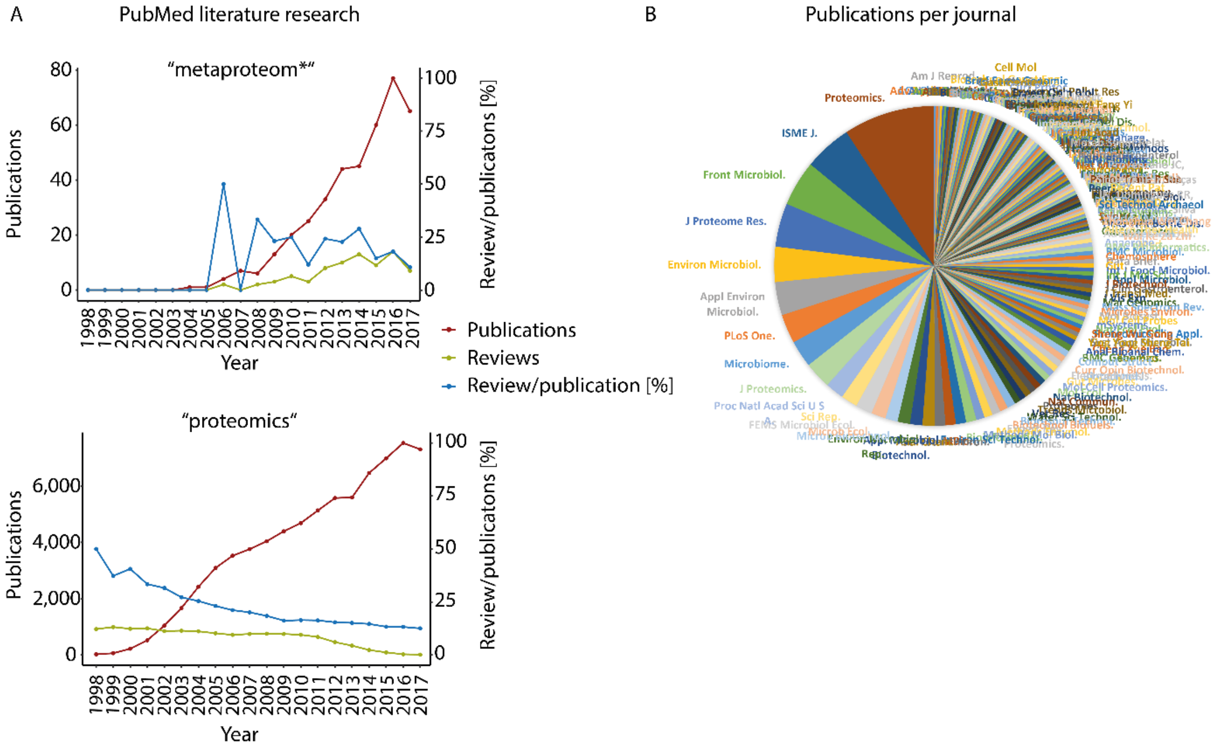

Supplementary Figure S7: Publications in the metaproteomic and proteomic field. PubMed search statistics for *'metaproteom*'* and *'proteomics'*. (a) Development of publications in the field of metaproteomic in comparison to proteomics. Total number of publications in relation to the number of reviews in the last 20 years is shown. (b) Pie chart shows the number of publications for *'metaproteom*'* in Pubmed per Journal.
