# Supplementary material for: Challenges in Clinical Metaproteomics Highlighted by the Analysis of Acute Leukemia Patients with Gut Colonization by Multidrug-Resistant Enterobacteriaceae
Source: Proteomes. 2019 Jan 8;7(1):2. doi: 10.3390/proteomes7010002 (PMC6473847; doi:10.3390/proteomes7010002)
Supplement: Supplementary file 1 [file proteomes-07-00002-s001.zip › supplement/Supplementary_Material1.pdf]

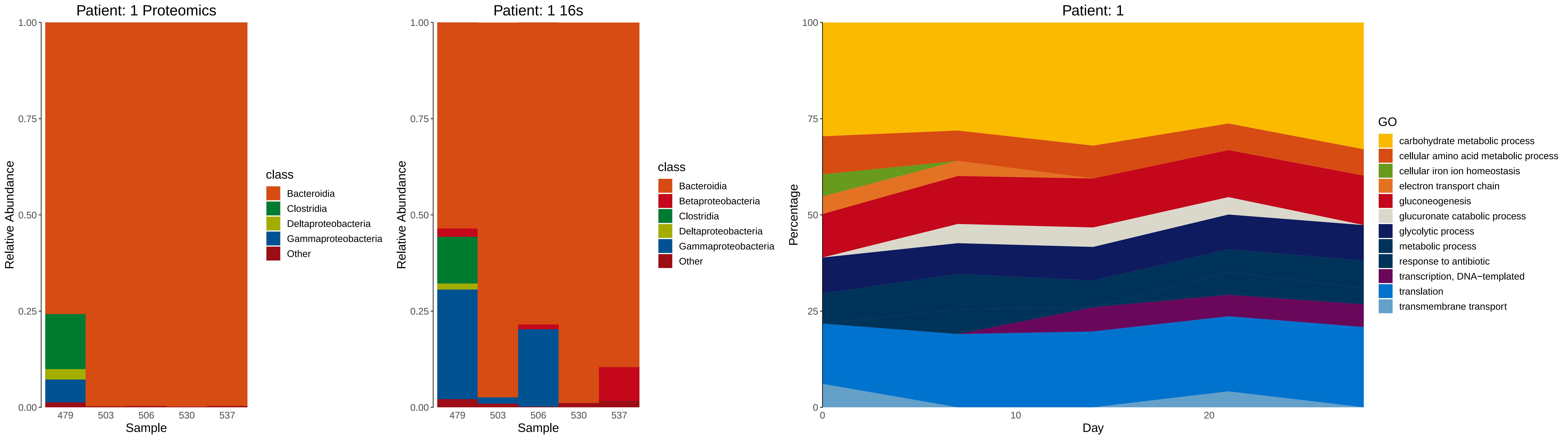
Patient: 1 Proteomics
Relative Abundance
1.00
0.75
0.50
0.25
0.00
479
503
506
530
537
Sample
class
Bacteroidia
Clostridia
Deltaproteobacteria
Gammaproteobacteria
Other
Patient: 1 16s
Relative Abundance
1.00
0.75
0.50
0.25
0.00
479
503
506
530
537
Sample
class
Bacteroidia
Betaproteobacteria
Clostridia
Deltaproteobacteria
Gammaproteobacteria
Other
Patient: 1
Percentage
100
75
50
25
0
0
10
20
Day
GO
carbohydrate metabolic process
cellular amino acid metabolic process
cellular iron ion homeostasis
electron transport chain
gluconeogenesis
glucuronate catabolic process
glycolytic process
metabolic process
response to antibiotic
transcription, DNA−templated
translation
transmembrane transport

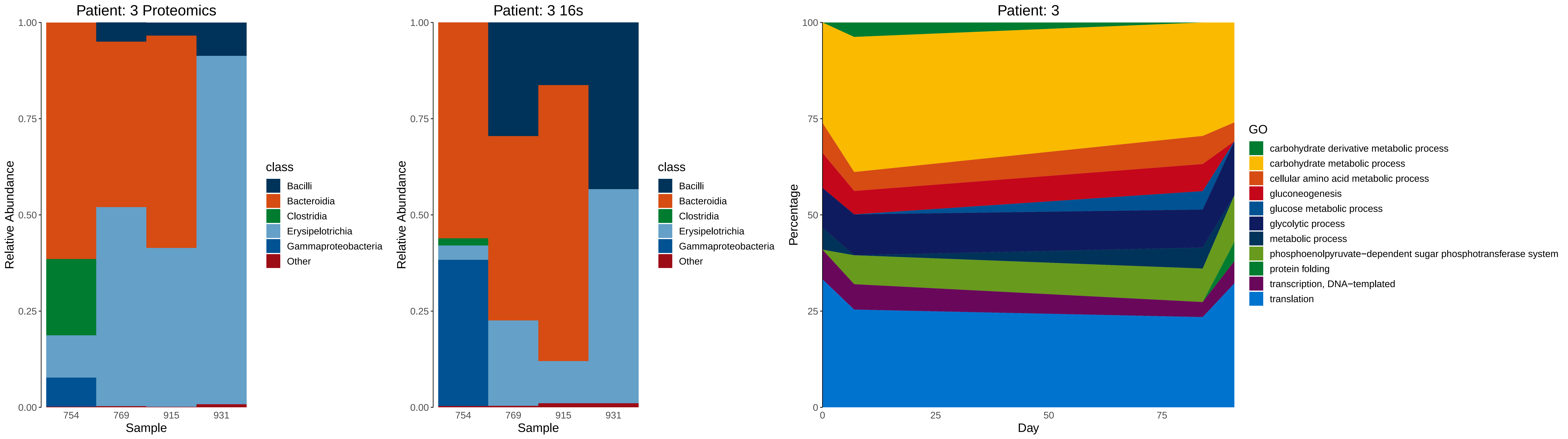

Patient: 3 Proteomics
Relative Abundance
1.00
0.75
0.50
0.25
0.00
754
769
915
931
Sample
class
Bacilli
Bacteroidia
Clostridia
Erysipelotrichia
Gammaproteobacteria
Other
Patient: 3 16s
Relative Abundance
1.00
0.75
0.50
0.25
0.00
754
769
915
931
Sample
class
Bacilli
Bacteroidia
Clostridia
Erysipelotrichia
Gammaproteobacteria
Other
Patient: 3
Percentage
100
75
50
25
0
0
25
50
75
Day
GO
carbohydrate derivative metabolic process
carbohydrate metabolic process
cellular amino acid metabolic process
gluconeogenesis
glucose metabolic process
glycolytic process
metabolic process
phosphoenolpyruvate−dependent sugar phosphotransferase system
protein folding
transcription, DNA−templated
translation

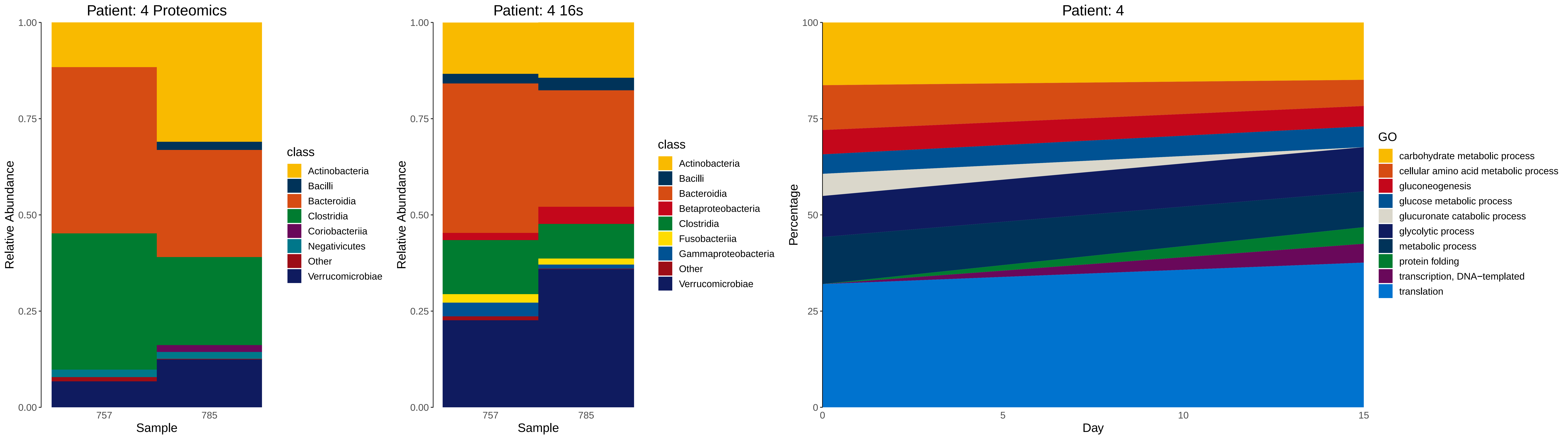
Patient: 4 Proteomics
Relative Abundance
1.00
0.75
0.50
0.25
0.00
757
785
Sample
class
Actinobacteria
Bacilli
Bacteroidia
Clostridia
Coriobacteriia
Negativicutes
Other
Verrucomicrobiae
Patient: 4 16s
Relative Abundance
1.00
0.75
0.50
0.25
0.00
757
785
Sample
class
Actinobacteria
Bacilli
Bacteroidia
Betaproteobacteria
Clostridia
Fusobacteriia
Gammaproteobacteria
Other
Verrucomicrobiae
Patient: 4
Percentage
100
75
50
25
0
0
5
10
15
Day
GO
carbohydrate metabolic process
cellular amino acid metabolic process
gluconeogenesis
glucose metabolic process
glucuronate catabolic process
glycolytic process
metabolic process
protein folding
transcription, DNA−templated
translation

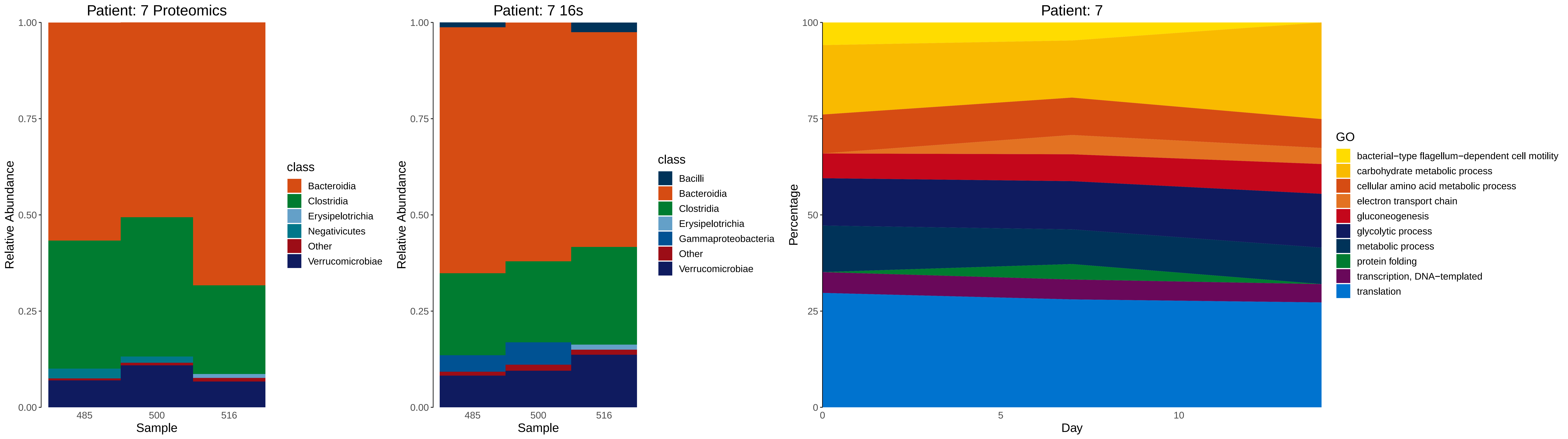

Patient: 7 Proteomics
Relative Abundance
Sample
485
500
516
class
Bacteroidia
Clostridia
Erysipelotrichia
Negativicutes
Other
Verrucomicrobiae
Patient: 7 16s
Relative Abundance
Sample
class
Bacilli
Bacteroidia
Clostridia
Erysipelotrichia
Gammaproteobacteria
Other
Verrucomicrobiae
Patient: 7
Percentage
Day
GO
bacterial−type flagellum−dependent cell motility
carbohydrate metabolic process
cellular amino acid metabolic process
electron transport chain
gluconeogenesis
glycolytic process
metabolic process
protein folding
transcription, DNA−templated
translation

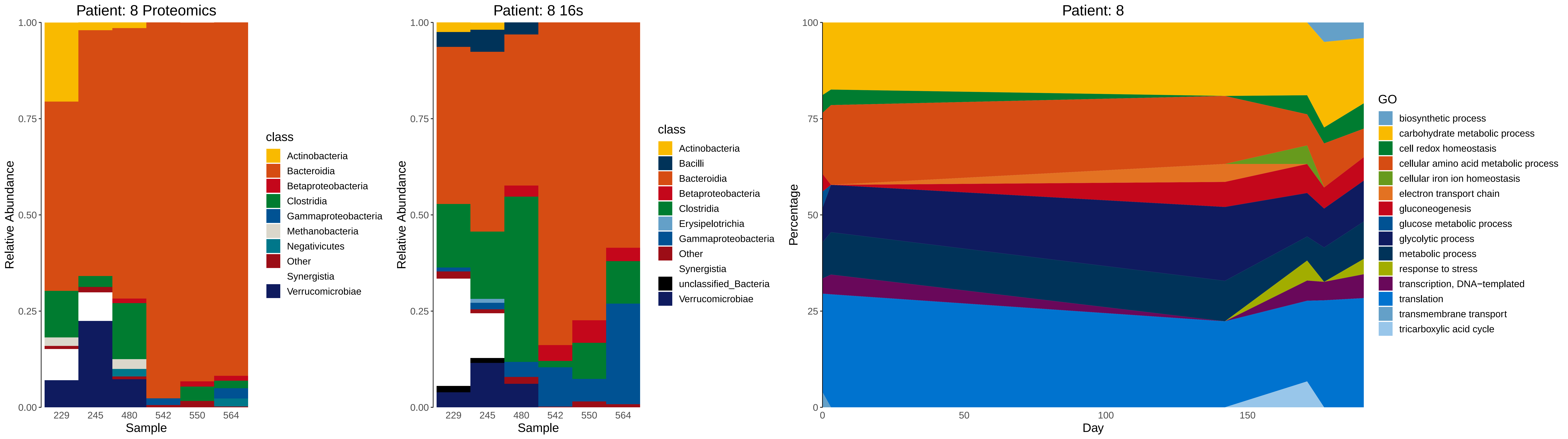

Patient: 8 Proteomics
Relative Abundance
Sample
229
245
480
542
550
564
class
Actinobacteria
Bacteroidia
Betaproteobacteria
Clostridia
Gammaproteobacteria
Methanobacteria
Negativicutes
Other
Synergistia
Verrucomicrobiae
Patient: 8 16s
Bacilli
Erysipelotrichia
unclassified_Bacteria
Patient: 8
Percentage
Day
GO
biosynthetic process
carbohydrate metabolic process
cell redox homeostasis
cellular amino acid metabolic process
cellular iron ion homeostasis
electron transport chain
gluconeogenesis
glucose metabolic process
glycolytic process
metabolic process
response to stress
transcription, DNA−templated
translation
transmembrane transport
tricarboxylic acid cycle

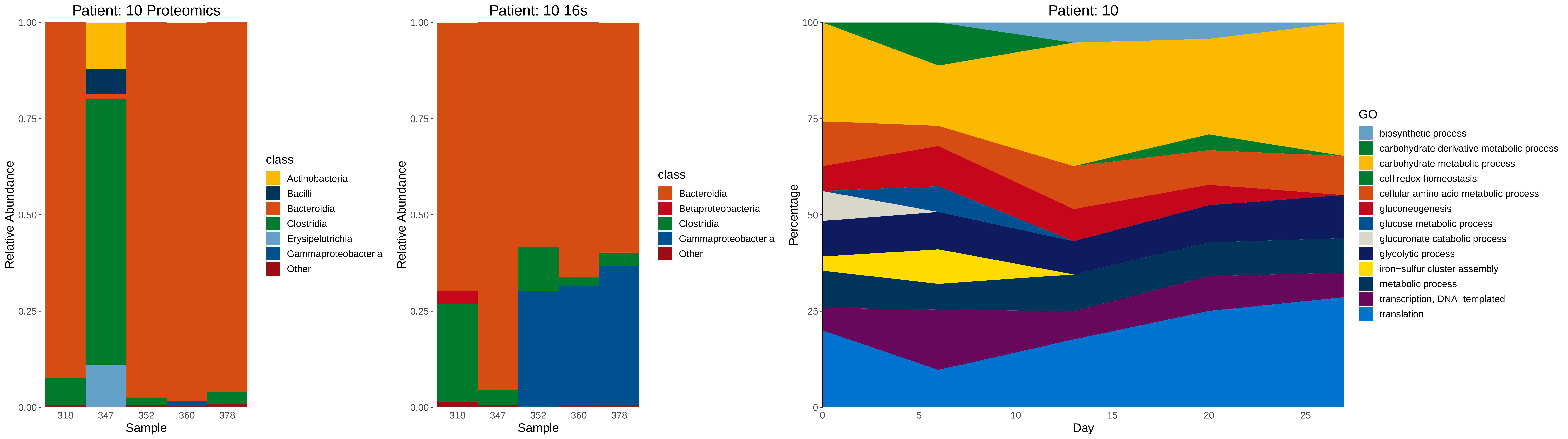

Patient: 10 Proteomics
Relative Abundance
1.00
0.75
0.50
0.25
0.00
318
347
352
360
378
Sample
class
Actinobacteria
Bacilli
Bacteroidia
Clostridia
Erysipelotrichia
Gammaproteobacteria
Other
Patient: 10 16s
Relative Abundance
Sample
class
Bacteroidia
Betaproteobacteria
Clostridia
Gammaproteobacteria
Other
Patient: 10
Percentage
100
75
50
25
0
0
5
10
15
20
25
Day
GO
biosynthetic process
carbohydrate derivative metabolic process
carbohydrate metabolic process
cell redox homeostasis
cellular amino acid metabolic process
gluconeogenesis
glucose metabolic process
glucuronate catabolic process
glycolytic process
iron−sulfur cluster assembly
metabolic process
transcription, DNA−templated
translation

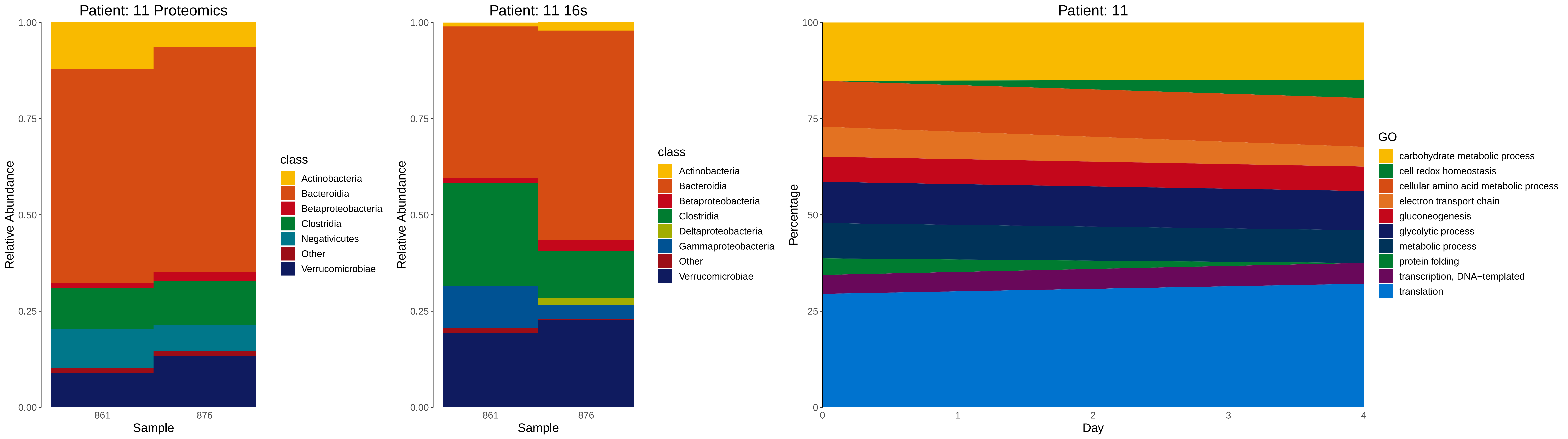
Patient: 11 Proteomics
Relative Abundance
1.00
0.75
0.50
0.25
0.00
861
876
Sample
class
Actinobacteria
Bacteroidia
Betaproteobacteria
Clostridia
Negativicutes
Other
Verrucomicrobiae
Patient: 11 16s
Relative Abundance
1.00
0.75
0.50
0.25
0.00
861
876
Sample
class
Actinobacteria
Bacteroidia
Betaproteobacteria
Clostridia
Deltaproteobacteria
Gammaproteobacteria
Other
Verrucomicrobiae
Patient: 11
Percentage
100
75
50
25
0
0
1
2
3
4
Day
GO
carbohydrate metabolic process
cell redox homeostasis
cellular amino acid metabolic process
electron transport chain
gluconeogenesis
glycolytic process
metabolic process
protein folding
transcription, DNA−templated
translation

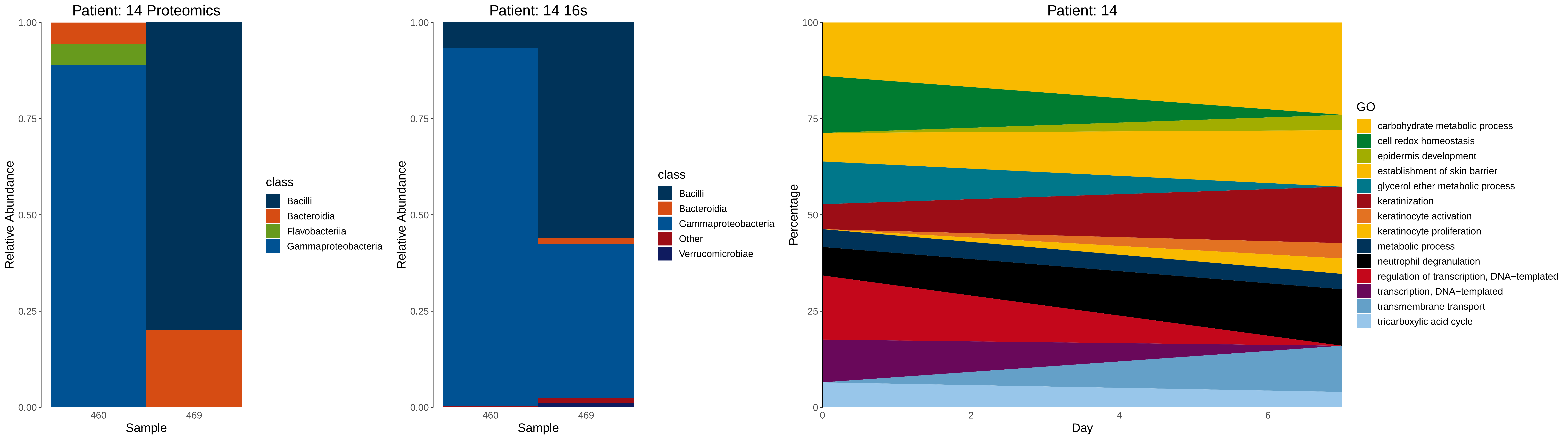

Patient: 14 Proteomics
Relative Abundance
1.00
0.75
0.50
0.25
0.00
460
469
Sample
class
Bacilli
Bacteroidia
Flavobacteriia
Gammaproteobacteria
Patient: 14 16s
Relative Abundance
1.00
0.75
0.50
0.25
0.00
460
469
Sample
class
Bacilli
Bacteroidia
Gammaproteobacteria
Other
Verrucomicrobiae
Patient: 14
Percentage
100
75
50
25
0
0
2
4
6
Day
GO
carbohydrate metabolic process
cell redox homeostasis
epidermis development
establishment of skin barrier
glycerol ether metabolic process
keratinization
keratinocyte activation
keratinocyte proliferation
metabolic process
neutrophil degranulation
regulation of transcription, DNA−templated
transcription, DNA−templated
transmembrane transport
tricarboxylic acid cycle

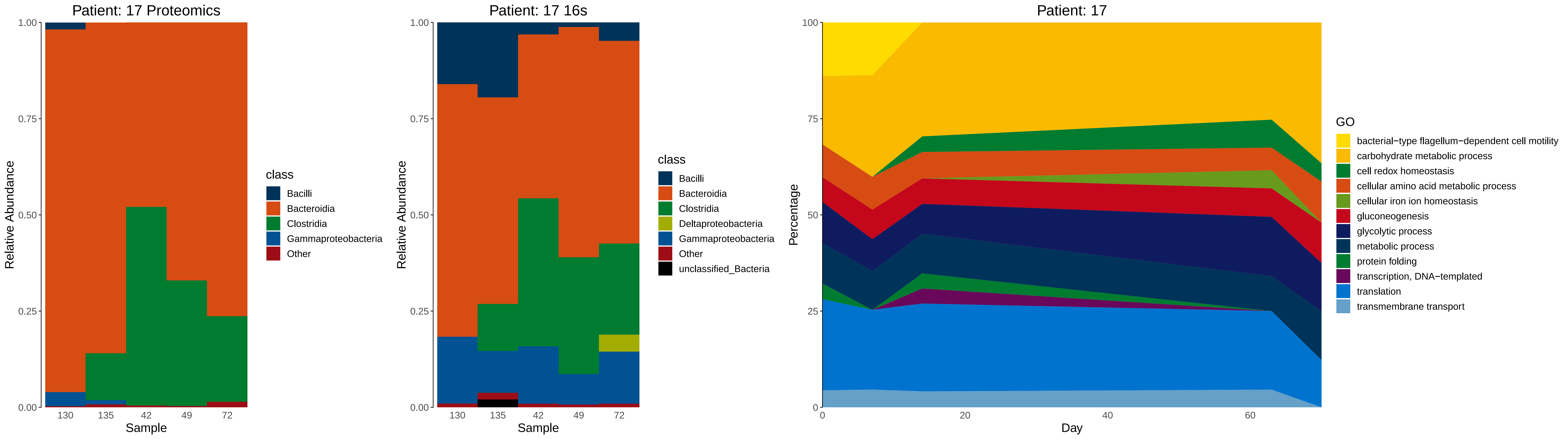

Patient: 17 Proteomics
Relative Abundance
1.00
0.75
0.50
0.25
0.00
130
135
42
49
72
Sample
class
Bacilli
Bacteroidia
Clostridia
Gammaproteobacteria
Other
Patient: 17 16s
Relative Abundance
Sample
class
Bacilli
Bacteroidia
Clostridia
Deltaproteobacteria
Gammaproteobacteria
Other
unclassified_Bacteria
Patient: 17
Percentage
100
75
50
25
0
20
40
60
Day
GO
bacterial−type flagellum−dependent cell motility
carbohydrate metabolic process
cell redox homeostasis
cellular amino acid metabolic process
cellular iron ion homeostasis
gluconeogenesis
glycolytic process
metabolic process
protein folding
transcription, DNA−templated
translation
transmembrane transport

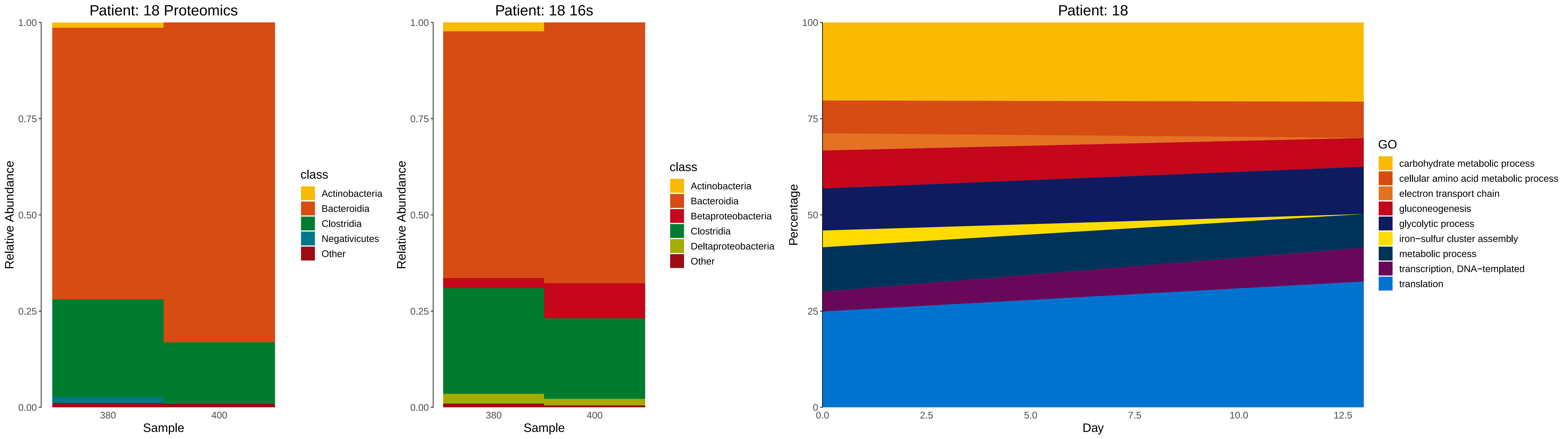
Patient: 18 Proteomics
Relative Abundance
1.00
0.75
0.50
0.25
0.00
380
400
Sample
class
Actinobacteria
Bacteroidia
Clostridia
Negativicutes
Other
Patient: 18 16s
Relative Abundance
1.00
0.75
0.50
0.25
0.00
380
400
Sample
class
Actinobacteria
Bacteroidia
Betaproteobacteria
Clostridia
Deltaproteobacteria
Other
Patient: 18
Percentage
100
75
50
25
0
0.0
2.5
5.0
7.5
10.0
12.5
Day
GO
carbohydrate metabolic process
cellular amino acid metabolic process
electron transport chain
gluconeogenesis
glycolytic process
iron−sulfur cluster assembly
metabolic process
transcription, DNA−templated
translation

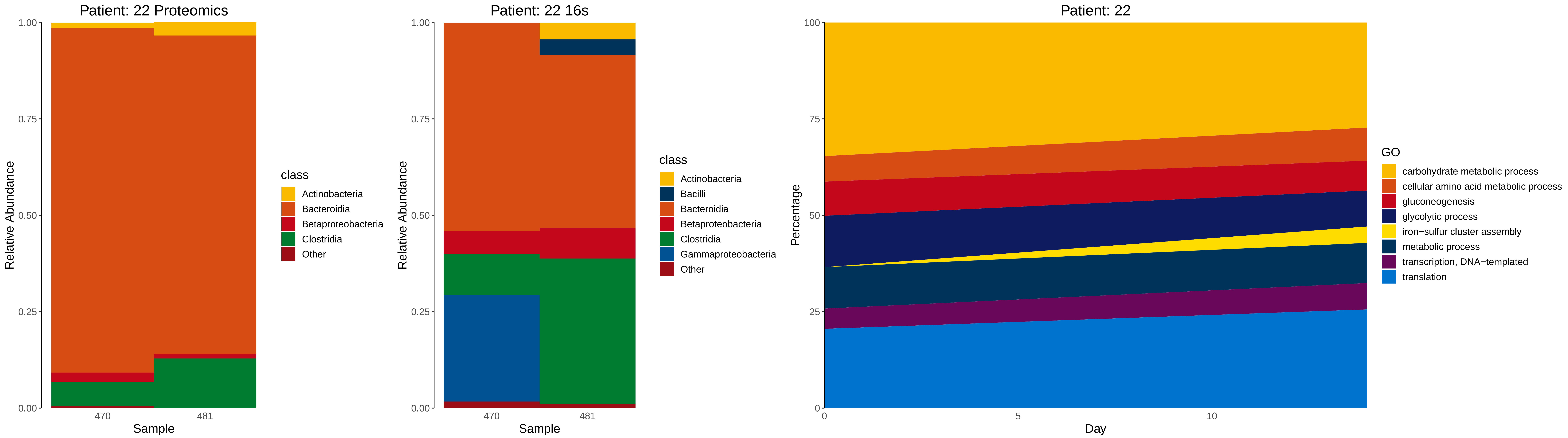
Patient: 22 Proteomics
Relative Abundance
1.00
0.75
0.50
0.25
0.00
470
481
Sample
class
Actinobacteria
Bacteroidia
Betaproteobacteria
Clostridia
Other
Patient: 22 16s
Relative Abundance
1.00
0.75
0.50
0.25
0.00
470
481
Sample
class
Actinobacteria
Bacilli
Bacteroidia
Betaproteobacteria
Clostridia
Gammaproteobacteria
Other
Patient: 22
Percentage
100
75
50
25
0
0
5
10
Day
GO
carbohydrate metabolic process
cellular amino acid metabolic process
gluconeogenesis
glycolytic process
iron−sulfur cluster assembly
metabolic process
transcription, DNA−templated
translation

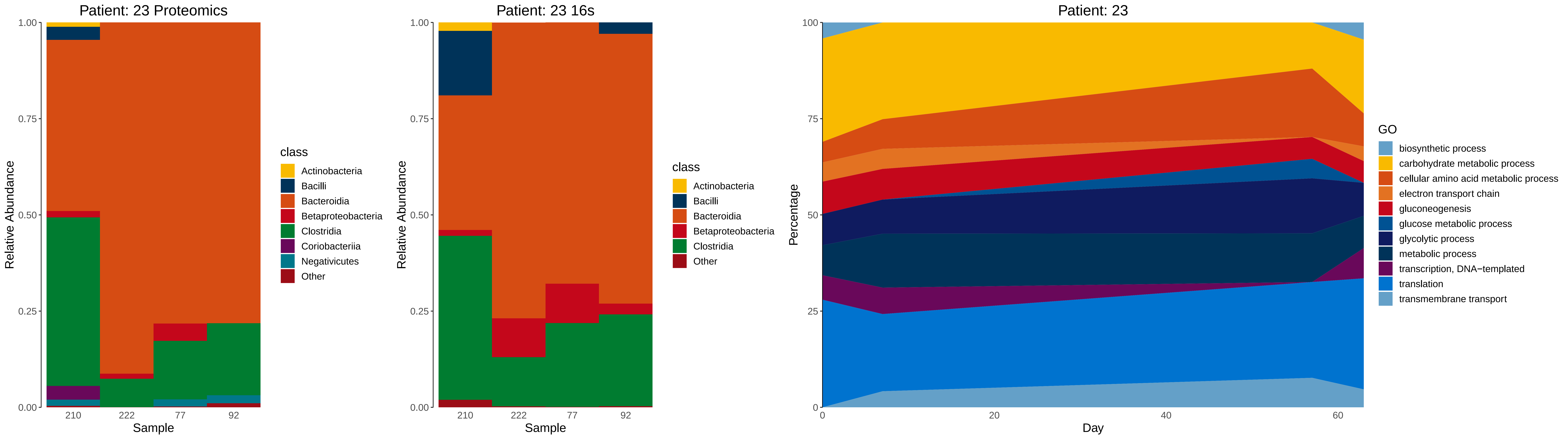
Patient: 23 Proteomics
Relative Abundance
1.00
0.75
0.50
0.25
0.00
210
222
77
92
Sample
class
Actinobacteria
Bacilli
Bacteroidia
Betaproteobacteria
Clostridia
Coriobacteriia
Negativicutes
Other
Patient: 23 16s
Relative Abundance
1.00
0.75
0.50
0.25
0.00
210
222
77
92
Sample
class
Actinobacteria
Bacilli
Bacteroidia
Betaproteobacteria
Clostridia
Other
Patient: 23
Percentage
100
75
50
25
0
0
20
40
60
Day
GO
biosynthetic process
carbohydrate metabolic process
cellular amino acid metabolic process
electron transport chain
gluconeogenesis
glucose metabolic process
glycolytic process
metabolic process
transcription, DNA−templated
translation
transmembrane transport

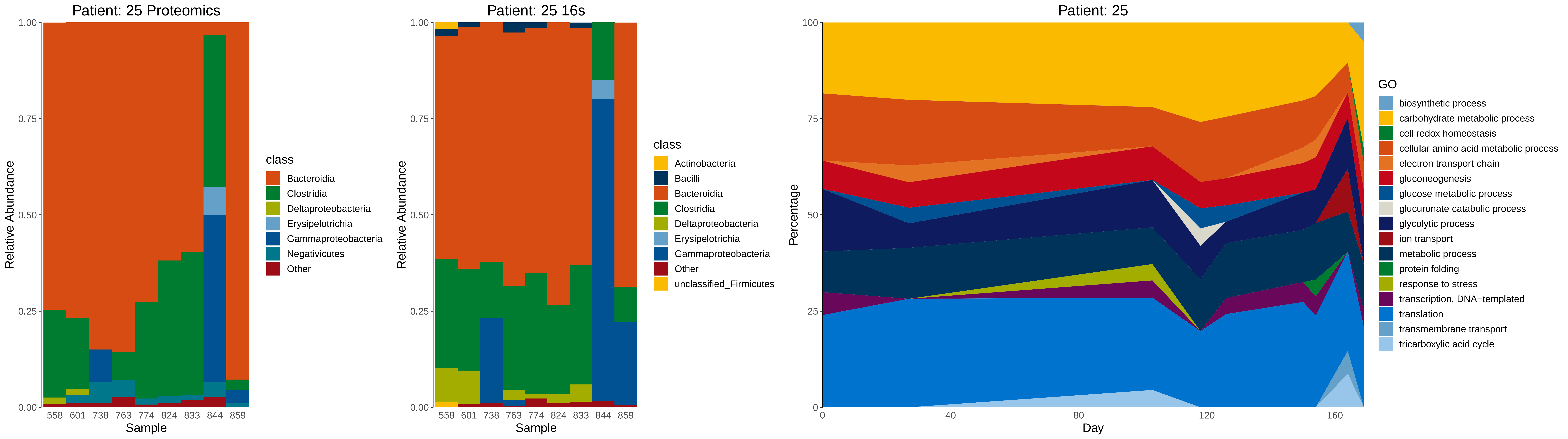
Patient: 25 Proteomics
Relative Abundance
1.00
0.75
0.50
0.25
0.00
558 601 738 763 774 824 833 844 859
Sample
class
Bacteroidia
Clostridia
Deltaproteobacteria
Erysipelotrichia
Gammaproteobacteria
Negativicutes
Other
Patient: 25 16s
Relative Abundance
1.00
0.75
0.50
0.25
0.00
558 601 738 763 774 824 833 844 859
Sample
class
Actinobacteria
Bacilli
Bacteroidia
Clostridia
Deltaproteobacteria
Erysipelotrichia
Gammaproteobacteria
Other
unclassified_Firmicutes
Patient: 25
Percentage
100
75
50
25
0
0 40 80 120 160
Day
GO
biosynthetic process
carbohydrate metabolic process
cell redox homeostasis
cellular amino acid metabolic process
electron transport chain
gluconeogenesis
glucose metabolic process
glucuronate catabolic process
glycolytic process
ion transport
metabolic process
protein folding
response to stress
transcription, DNA−templated
translation
transmembrane transport
tricarboxylic acid cycle

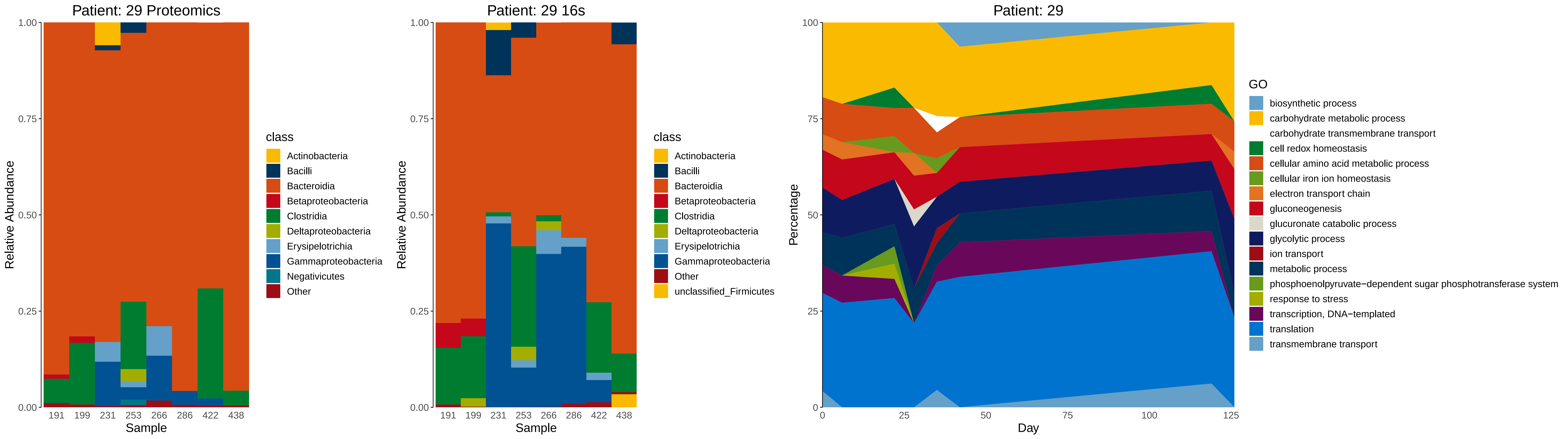
Patient: 29 Proteomics
Relative Abundance
Sample
191
199
231
253
266
286
422
438
1.00
0.75
0.50
0.25
0.00
class
Actinobacteria
Bacilli
Bacteroidia
Betaproteobacteria
Clostridia
Deltaproteobacteria
Erysipelotrichia
Gammaproteobacteria
Negativicutes
Other
Patient: 29 16s
Relative Abundance
Sample
class
Actinobacteria
Bacilli
Bacteroidia
Betaproteobacteria
Clostridia
Deltaproteobacteria
Erysipelotrichia
Gammaproteobacteria
Other
unclassified_Firmicutes
Patient: 29
Percentage
Day
0
25
50
75
100
125
GO
biosynthetic process
carbohydrate metabolic process
carbohydrate transmembrane transport
cell redox homeostasis
cellular amino acid metabolic process
cellular iron ion homeostasis
electron transport chain
gluconeogenesis
glucuronate catabolic process
glycolytic process
ion transport
metabolic process
phosphoenolpyruvate−dependent sugar phosphotransferase system
response to stress
transcription, DNA−templated
translation
transmembrane transport

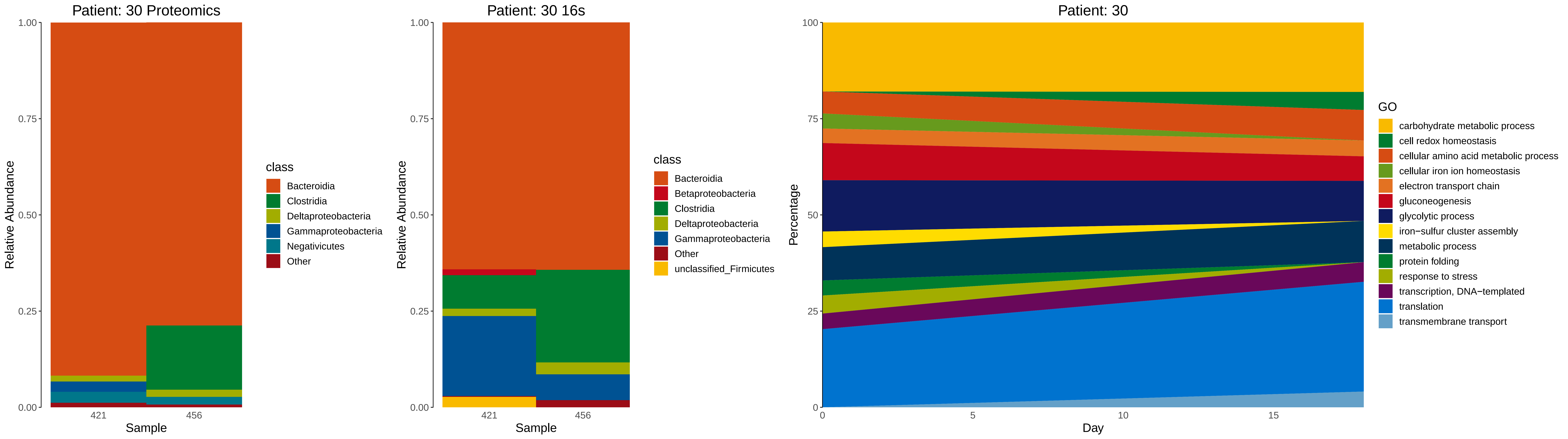

Patient: 30 Proteomics
Relative Abundance
1.00
0.75
0.50
0.25
0.00
421
456
Sample
class
Bacteroidia
Clostridia
Deltaproteobacteria
Gammaproteobacteria
Negativicutes
Other
Patient: 30 16s
Relative Abundance
1.00
0.75
0.50
0.25
0.00
421
456
Sample
class
Bacteroidia
Betaproteobacteria
Clostridia
Deltaproteobacteria
Gammaproteobacteria
Other
unclassified_Firmicutes
Patient: 30
Percentage
100
75
50
25
0
0
5
10
15
Day
GO
carbohydrate metabolic process
cell redox homeostasis
cellular amino acid metabolic process
cellular iron ion homeostasis
electron transport chain
gluconeogenesis
glycolytic process
iron−sulfur cluster assembly
metabolic process
protein folding
response to stress
transcription, DNA−templated
translation
transmembrane transport

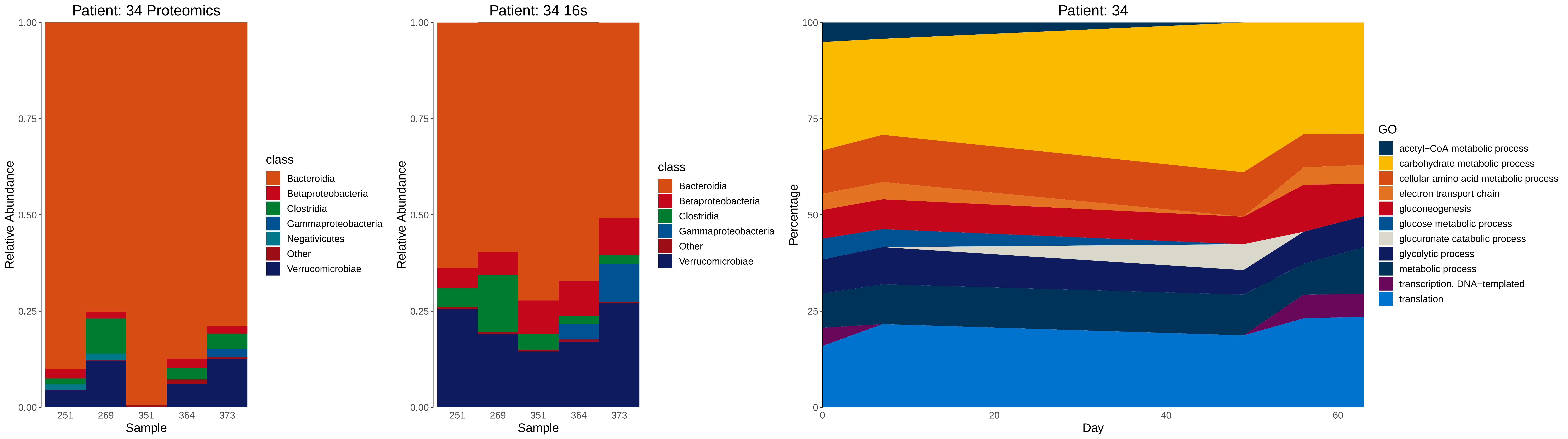

Patient: 34 Proteomics
Relative Abundance
Sample
251
269
351
364
373
class
Bacteroidia
Betaproteobacteria
Clostridia
Gammaproteobacteria
Negativicutes
Other
Verrucomicrobiae
Patient: 34 16s
Relative Abundance
Sample
class
Bacteroidia
Betaproteobacteria
Clostridia
Gammaproteobacteria
Other
Verrucomicrobiae
Patient: 34
Percentage
Day
GO
acetyl−CoA metabolic process
carbohydrate metabolic process
cellular amino acid metabolic process
electron transport chain
gluconeogenesis
glucose metabolic process
glucuronate catabolic process
glycolytic process
metabolic process
transcription, DNA−templated
translation

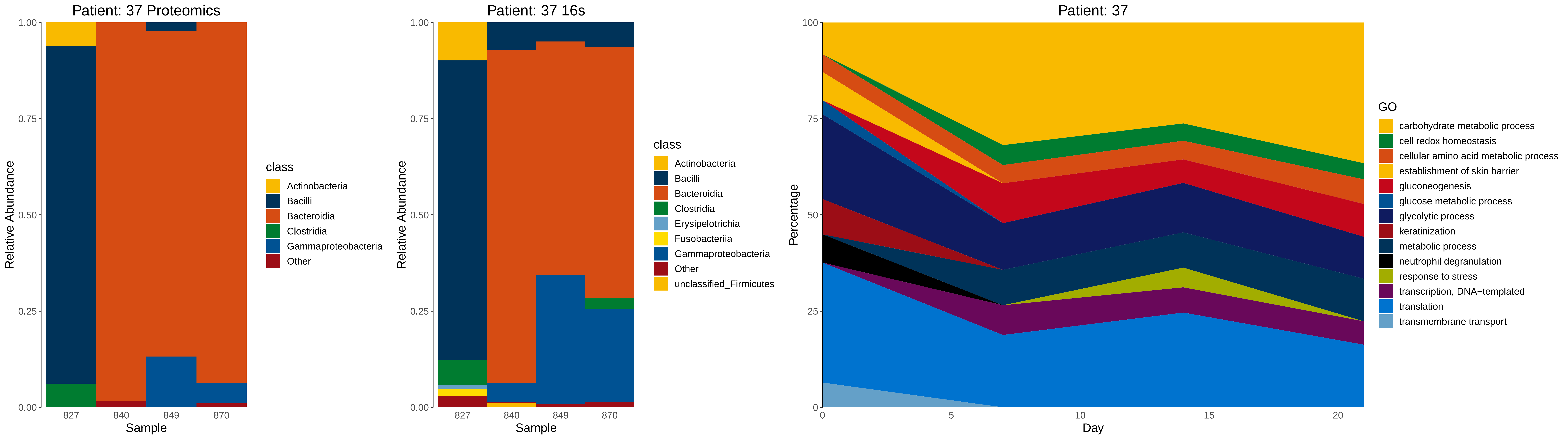

Patient: 37 Proteomics
Relative Abundance
1.00
0.75
0.50
0.25
0.00
827
840
849
870
Sample
class
Actinobacteria
Bacilli
Bacteroidia
Clostridia
Gammaproteobacteria
Other
Patient: 37 16s
Relative Abundance
1.00
0.75
0.50
0.25
0.00
827
840
849
870
Sample
class
Actinobacteria
Bacilli
Bacteroidia
Clostridia
Erysipelotrichia
Fusobacteriia
Gammaproteobacteria
Other
unclassified_Firmicutes
Patient: 37
Percentage
100
75
50
25
0
0
5
10
15
20
Day
GO
carbohydrate metabolic process
cell redox homeostasis
cellular amino acid metabolic process
establishment of skin barrier
gluconeogenesis
glucose metabolic process
glycolytic process
keratinization
metabolic process
neutrophil degranulation
response to stress
transcription, DNA−templated
translation
transmembrane transport

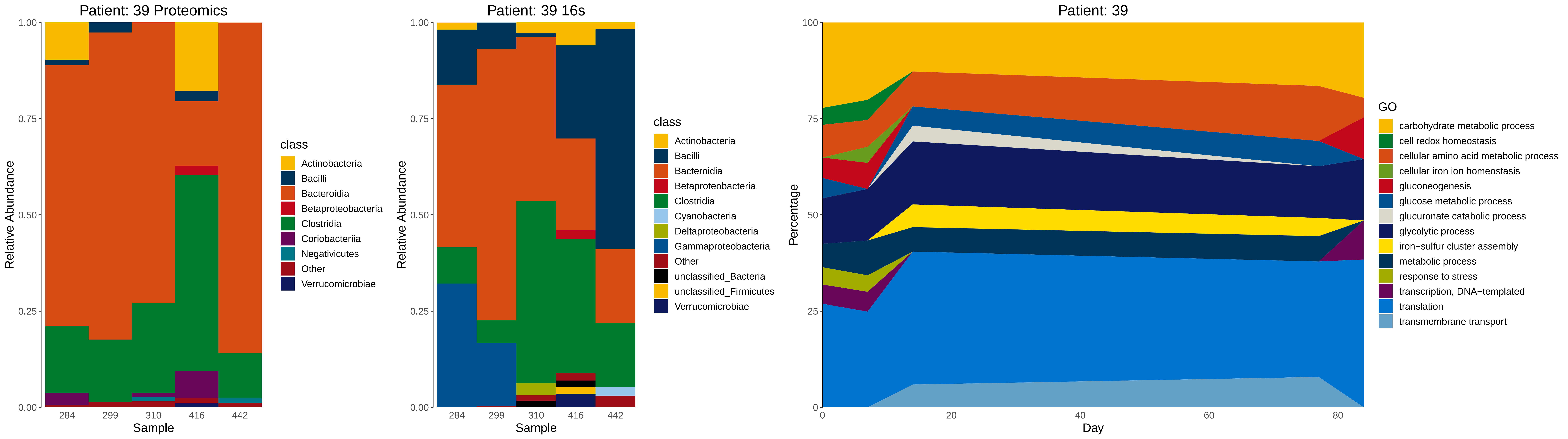

Patient: 39 Proteomics
Relative Abundance
Sample
284
299
310
416
442
0.00
0.25
0.50
0.75
1.00
class
Actinobacteria
Bacilli
Bacteroidia
Betaproteobacteria
Clostridia
Coriobacteriia
Negativicutes
Other
Verrucomicrobiae
Patient: 39 16s
class
Actinobacteria
Bacilli
Bacteroidia
Betaproteobacteria
Clostridia
Cyanobacteria
Deltaproteobacteria
Gammaproteobacteria
Other
unclassified_Bacteria
unclassified_Firmicutes
Verrucomicrobiae
Patient: 39
Percentage
Day
0
20
40
60
80
25
50
75
100
GO
carbohydrate metabolic process
cell redox homeostasis
cellular amino acid metabolic process
cellular iron ion homeostasis
gluconeogenesis
glucose metabolic process
glucuronate catabolic process
glycolytic process
iron−sulfur cluster assembly
metabolic process
response to stress
transcription, DNA−templated
translation
transmembrane transport

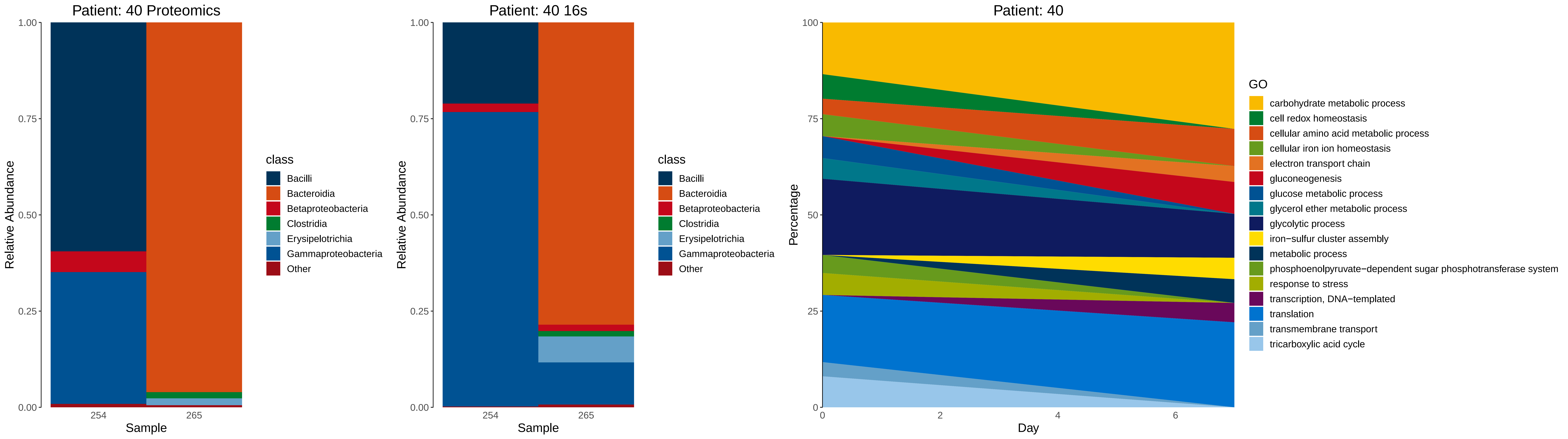
Patient: 40 Proteomics
Patient: 40 16s
Patient: 40
Relative Abundance
Percentage
Sample
Day
class
Bacilli
Bacteroidia
Betaproteobacteria
Clostridia
Erysipelotrichia
Gammaproteobacteria
Other
GO
carbohydrate metabolic process
cell redox homeostasis
cellular amino acid metabolic process
cellular iron ion homeostasis
electron transport chain
gluconeogenesis
glucose metabolic process
glycerol ether metabolic process
glycolytic process
iron−sulfur cluster assembly
metabolic process
phosphoenolpyruvate−dependent sugar phosphotransferase system
response to stress
transcription, DNA−templated
translation
transmembrane transport
tricarboxylic acid cycle
254
265
0.00
0.25
0.50
0.75
1.00
0
25
50
75
100
0
2
4
6

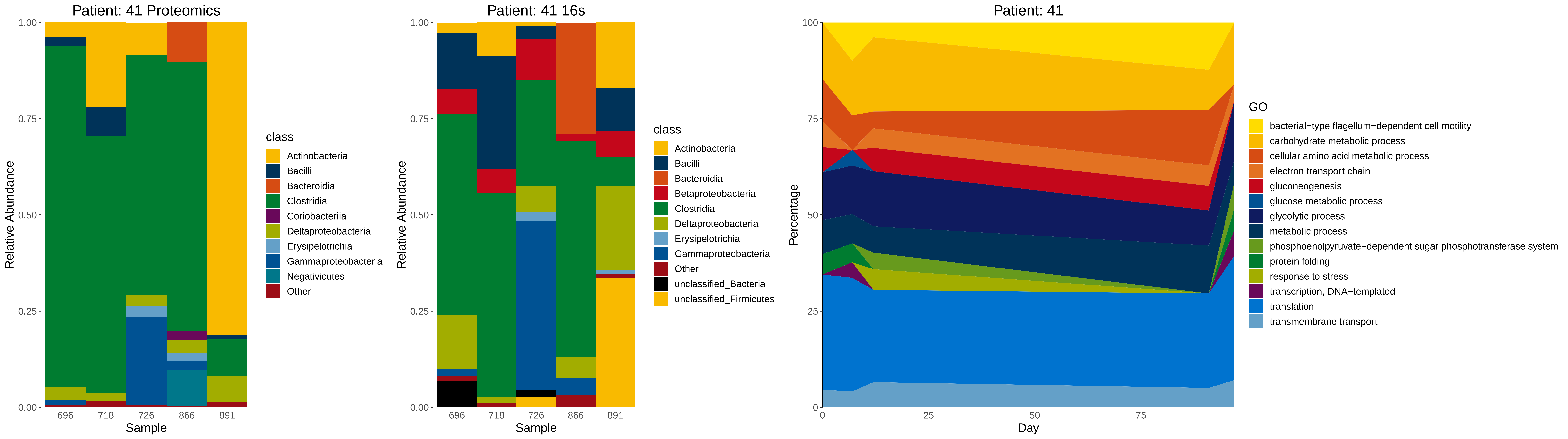

Patient: 41 Proteomics
Relative Abundance
0.00
0.25
0.50
0.75
1.00
696
718
726
866
891
Sample
class
Actinobacteria
Bacilli
Bacteroidia
Clostridia
Coriobacteriia
Deltaproteobacteria
Erysipelotrichia
Gammaproteobacteria
Negativicutes
Other
Patient: 41 16s
Relative Abundance
0.00
0.25
0.50
0.75
1.00
696
718
726
866
891
Sample
class
Actinobacteria
Bacilli
Bacteroidia
Betaproteobacteria
Clostridia
Deltaproteobacteria
Erysipelotrichia
Gammaproteobacteria
Other
unclassified_Bacteria
unclassified_Firmicutes
Patient: 41
Percentage
0
25
50
75
100
0
25
50
75
Day
GO
bacterial−type flagellum−dependent cell motility
carbohydrate metabolic process
cellular amino acid metabolic process
electron transport chain
gluconeogenesis
glucose metabolic process
glycolytic process
metabolic process
phosphoenolpyruvate−dependent sugar phosphotransferase system
protein folding
response to stress
transcription, DNA−templated
translation
transmembrane transport

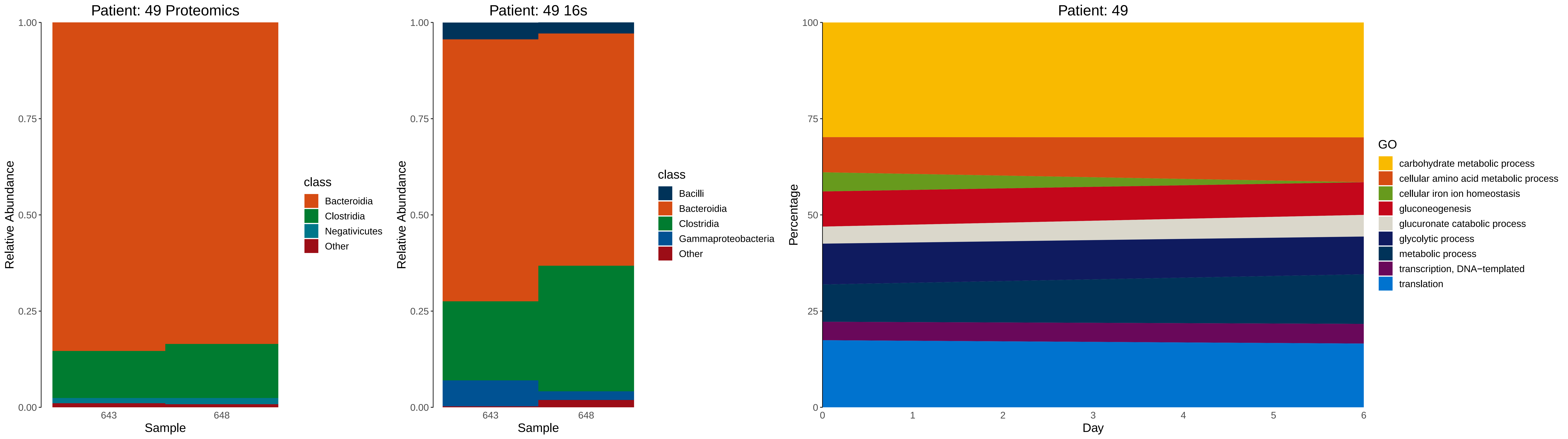

Patient: 49 Proteomics
Relative Abundance
1.00
0.75
0.50
0.25
0.00
643
648
Sample
class
Bacteroidia
Clostridia
Negativicutes
Other
Patient: 49 16s
Relative Abundance
Sample
class
Bacilli
Bacteroidia
Clostridia
Gammaproteobacteria
Other
Patient: 49
Percentage
100
75
50
25
0
0
1
2
3
4
5
6
Day
GO
carbohydrate metabolic process
cellular amino acid metabolic process
cellular iron ion homeostasis
gluconeogenesis
glucuronate catabolic process
glycolytic process
metabolic process
transcription, DNA−templated
translation

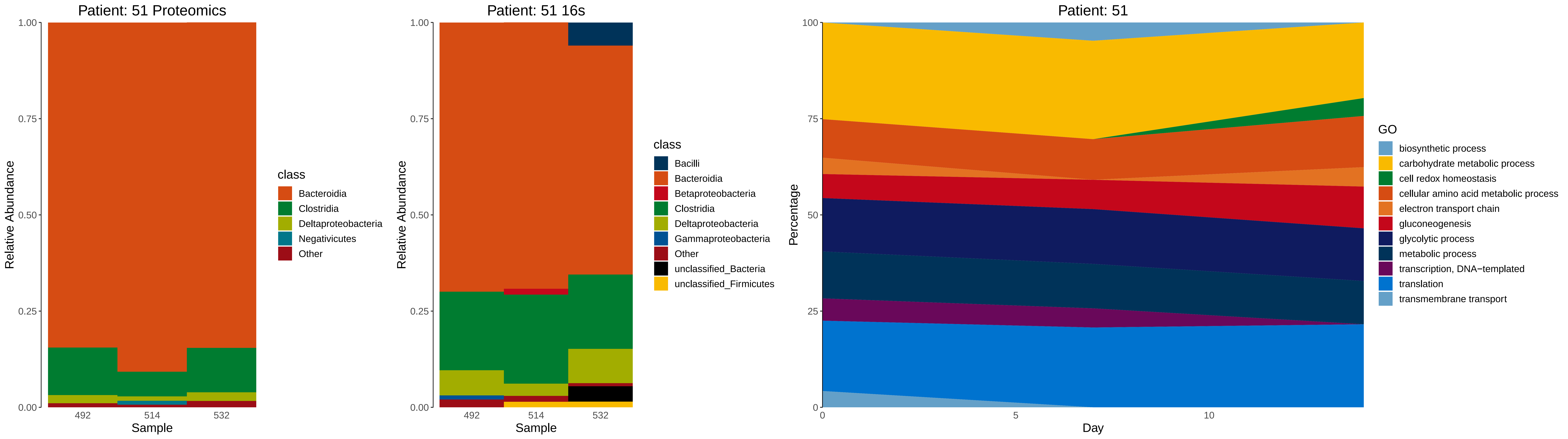
Patient: 51 Proteomics
Relative Abundance
Sample
492
514
532
class
Bacteroidia
Clostridia
Deltaproteobacteria
Negativicutes
Other
Patient: 51 16s
Relative Abundance
Sample
class
Bacilli
Bacteroidia
Betaproteobacteria
Clostridia
Deltaproteobacteria
Gammaproteobacteria
Other
unclassified_Bacteria
unclassified_Firmicutes
Patient: 51
Percentage
Day
GO
biosynthetic process
carbohydrate metabolic process
cell redox homeostasis
cellular amino acid metabolic process
electron transport chain
gluconeogenesis
glycolytic process
metabolic process
transcription, DNA−templated
translation
transmembrane transport

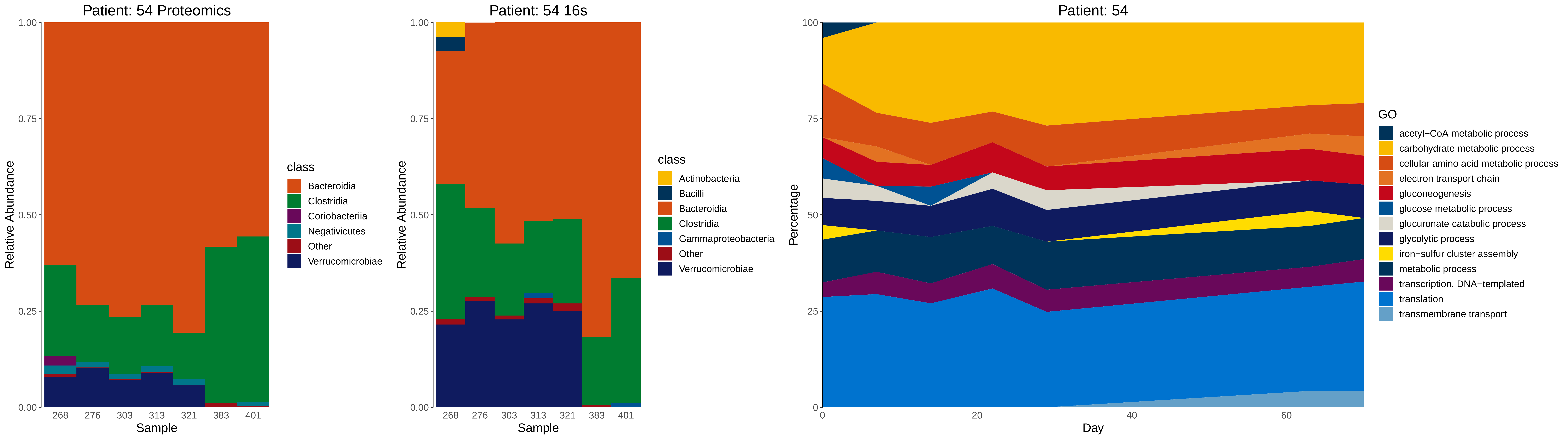
Patient: 54 Proteomics
Relative Abundance
Sample
268
276
303
313
321
383
401
1.00
0.75
0.50
0.25
0.00
class
Bacteroidia
Clostridia
Coriobacteriia
Negativicutes
Other
Verrucomicrobiae
Patient: 54 16s
Actinobacteria
Bacilli
Bacteroidia
Clostridia
Gammaproteobacteria
Other
Verrucomicrobiae
Patient: 54
Percentage
Day
0
20
40
60
100
75
50
25
GO
acetyl−CoA metabolic process
carbohydrate metabolic process
cellular amino acid metabolic process
electron transport chain
gluconeogenesis
glucose metabolic process
glucuronate catabolic process
glycolytic process
iron−sulfur cluster assembly
metabolic process
transcription, DNA−templated
translation
transmembrane transport

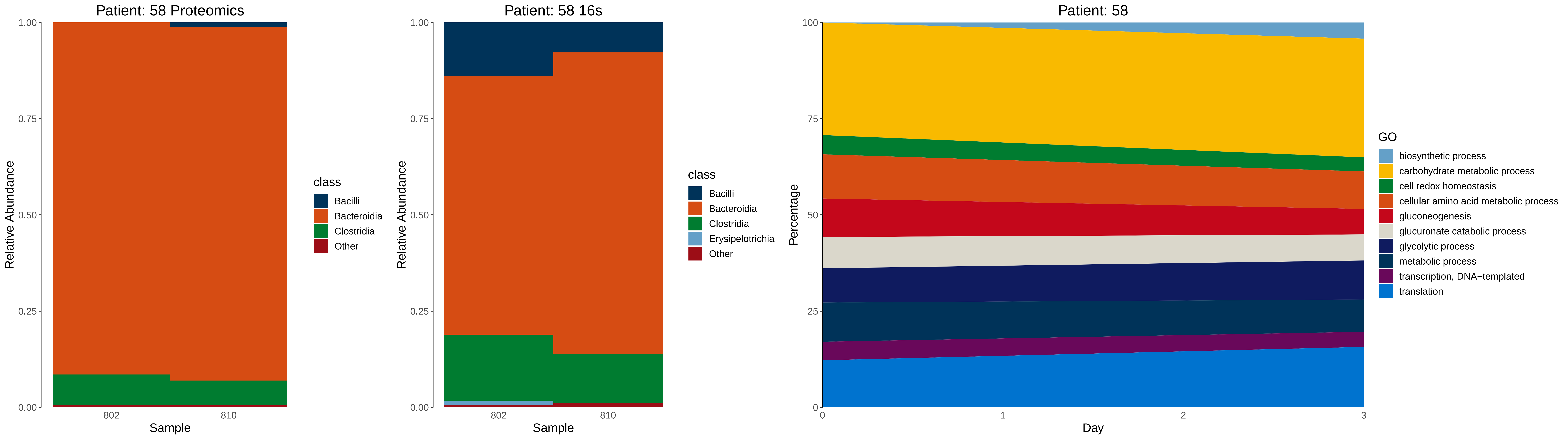

Patient: 58 Proteomics
Relative Abundance
Sample
802
810
1.00
0.75
0.50
0.25
0.00
class
Bacilli
Bacteroidia
Clostridia
Other
Patient: 58 16s
Relative Abundance
Sample
802
810
class
Bacilli
Bacteroidia
Clostridia
Erysipelotrichia
Other
Patient: 58
Percentage
Day
0
1
2
3
100
75
50
25
0
GO
biosynthetic process
carbohydrate metabolic process
cell redox homeostasis
cellular amino acid metabolic process
gluconeogenesis
glucuronate catabolic process
glycolytic process
metabolic process
transcription, DNA−templated
translation

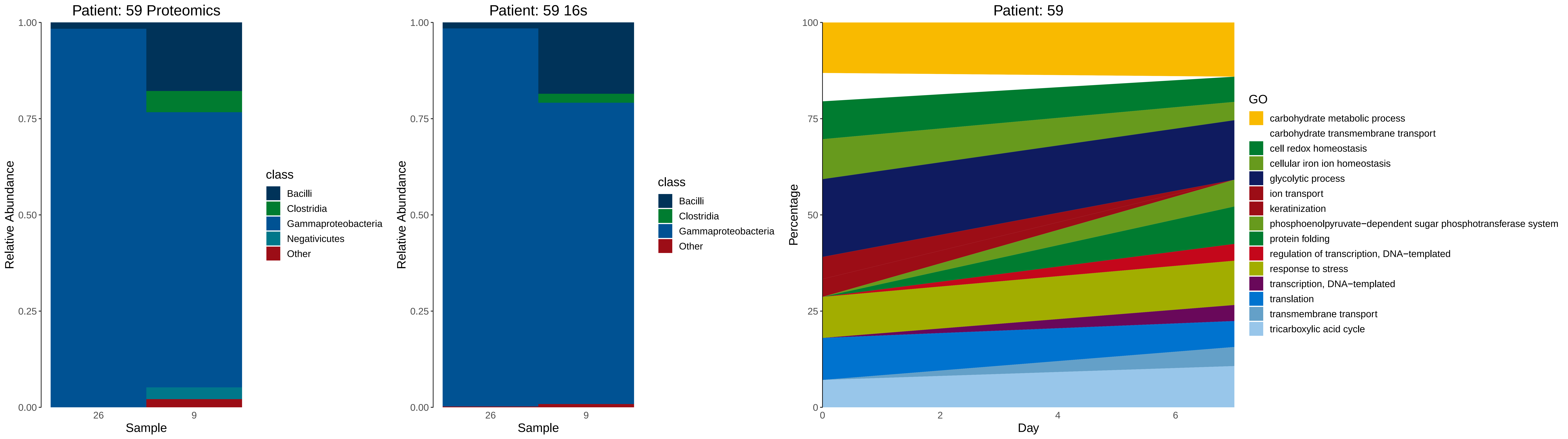

Patient: 59 Proteomics
Relative Abundance
1.00
0.75
0.50
0.25
0.00
26
9
Sample
class
Bacilli
Clostridia
Gammaproteobacteria
Negativicutes
Other
Patient: 59 16s
Relative Abundance
1.00
0.75
0.50
0.25
0.00
26
9
Sample
class
Bacilli
Clostridia
Gammaproteobacteria
Other
Patient: 59
Percentage
100
75
50
25
0
0
2
4
6
Day
GO
carbohydrate metabolic process
carbohydrate transmembrane transport
cell redox homeostasis
cellular iron ion homeostasis
glycolytic process
ion transport
keratinization
phosphoenolpyruvate−dependent sugar phosphotransferase system
protein folding
regulation of transcription, DNA−templated
response to stress
transcription, DNA−templated
translation
transmembrane transport
tricarboxylic acid cycle

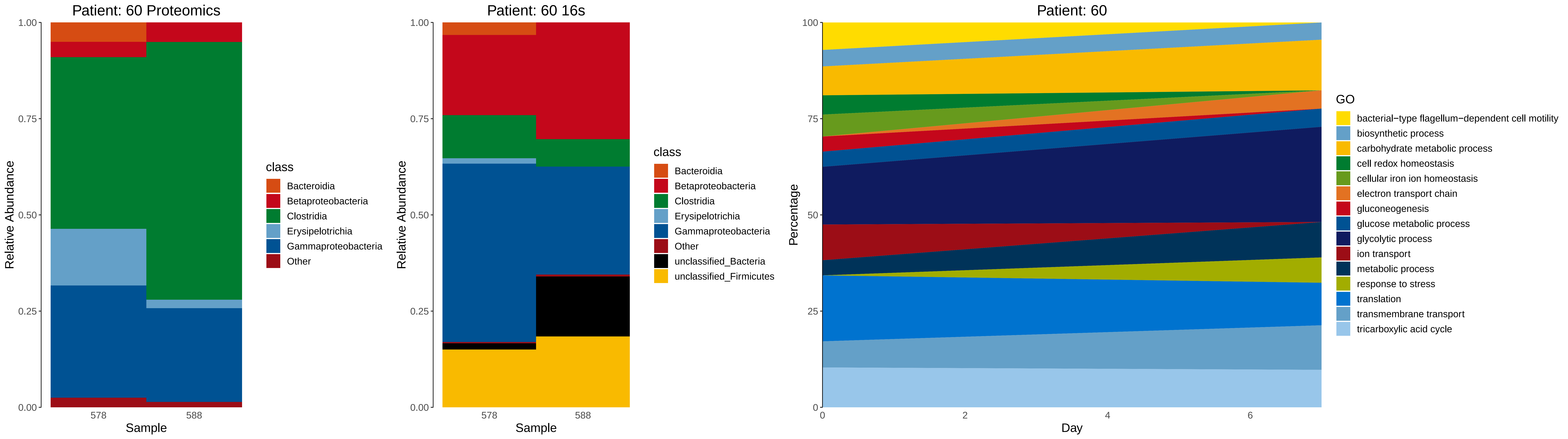

Patient: 60 Proteomics
Relative Abundance
1.00
0.75
0.50
0.25
0.00
578
588
Sample
class
Bacteroidia
Betaproteobacteria
Clostridia
Erysipelotrichia
Gammaproteobacteria
Other
Patient: 60 16s
Relative Abundance
1.00
0.75
0.50
0.25
0.00
578
588
Sample
class
Bacteroidia
Betaproteobacteria
Clostridia
Erysipelotrichia
Gammaproteobacteria
Other
unclassified_Bacteria
unclassified_Firmicutes
Patient: 60
Percentage
100
75
50
25
0
0
2
4
6
Day
GO
bacterial−type flagellum−dependent cell motility
biosynthetic process
carbohydrate metabolic process
cell redox homeostasis
cellular iron ion homeostasis
electron transport chain
gluconeogenesis
glucose metabolic process
glycolytic process
ion transport
metabolic process
response to stress
translation
transmembrane transport
tricarboxylic acid cycle

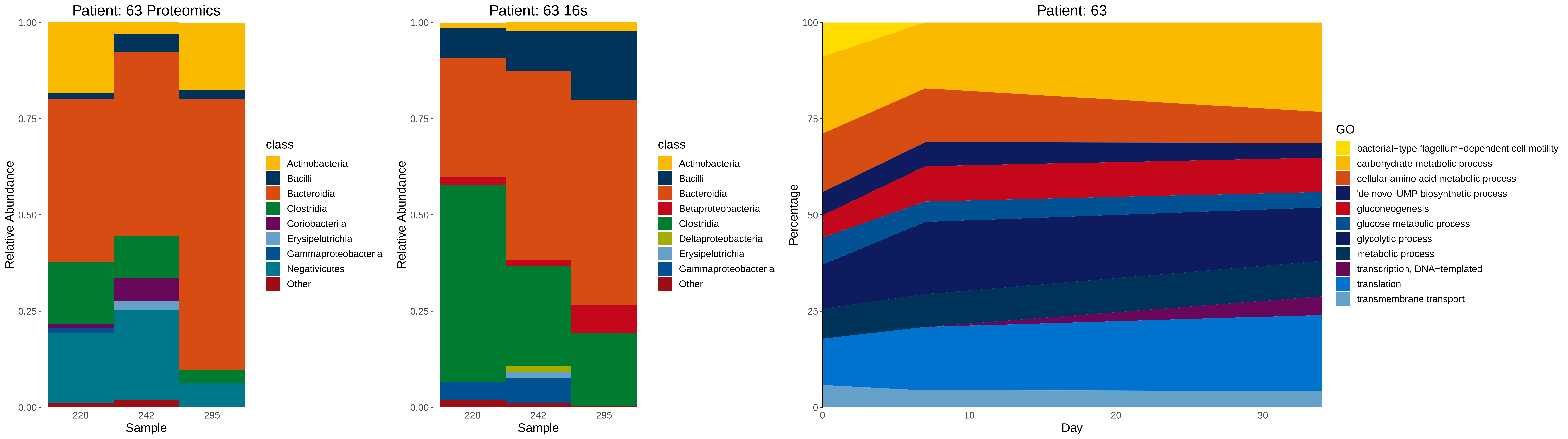
Patient: 63 Proteomics
Relative Abundance
Sample
228
242
295
class
Actinobacteria
Bacilli
Bacteroidia
Clostridia
Coriobacteriia
Erysipelotrichia
Gammaproteobacteria
Negativicutes
Other
Patient: 63 16s
Betaproteobacteria
Deltaproteobacteria
Patient: 63
Percentage
Day
GO
bacterial−type flagellum−dependent cell motility
carbohydrate metabolic process
cellular amino acid metabolic process
'de novo' UMP biosynthetic process
gluconeogenesis
glucose metabolic process
glycolytic process
metabolic process
transcription, DNA−templated
translation
transmembrane transport

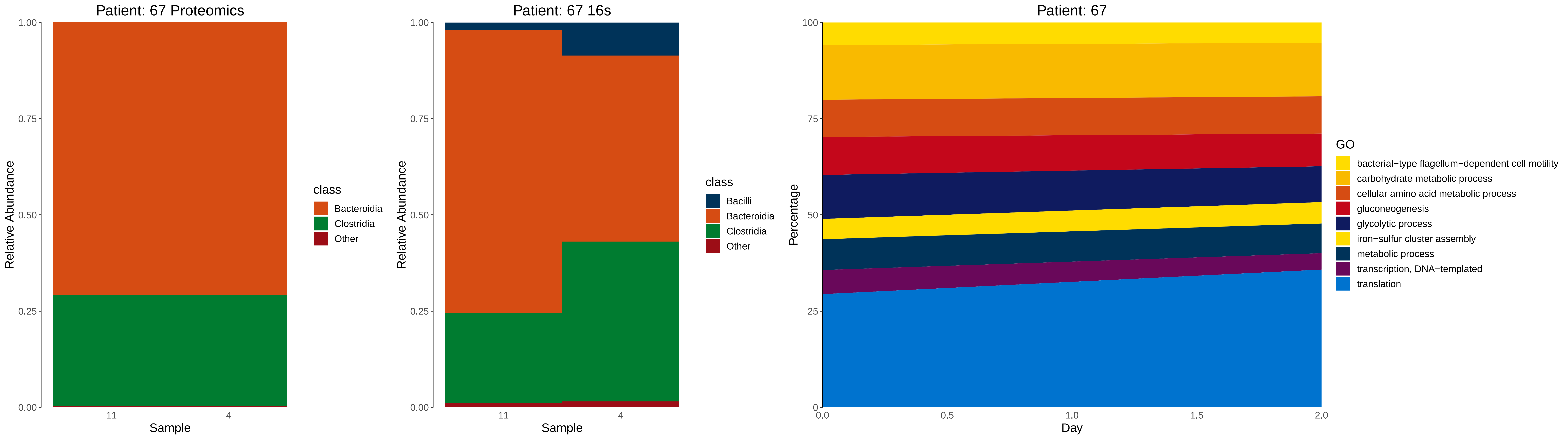
Patient: 67 Proteomics
Relative Abundance
1.00
0.75
0.50
0.25
0.00
11
4
Sample
class
Bacteroidia
Clostridia
Other
Patient: 67 16s
Relative Abundance
1.00
0.75
0.50
0.25
0.00
11
4
Sample
class
Bacilli
Bacteroidia
Clostridia
Other
Patient: 67
Percentage
100
75
50
25
0
0.0
0.5
1.0
1.5
2.0
Day
GO
bacterial−type flagellum−dependent cell motility
carbohydrate metabolic process
cellular amino acid metabolic process
gluconeogenesis
glycolytic process
iron−sulfur cluster assembly
metabolic process
transcription, DNA−templated
translation

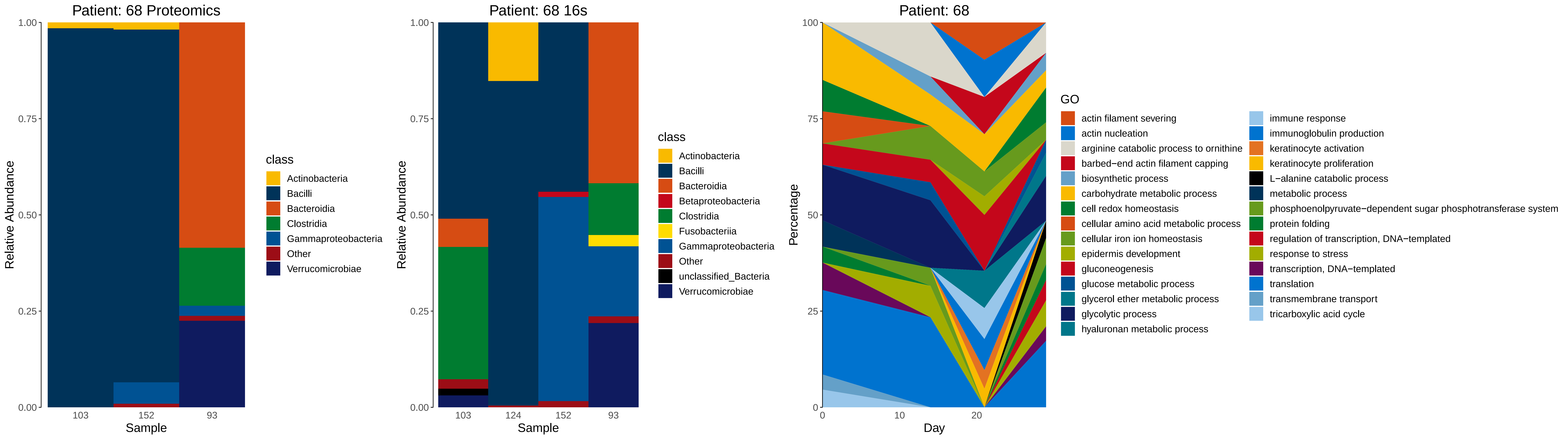
Patient: 68 Proteomics
Relative Abundance
1.00
0.75
0.50
0.25
0.00
103
152
93
Sample
class
Actinobacteria
Bacilli
Bacteroidia
Clostridia
Gammaproteobacteria
Other
Verrucomicrobiae
Patient: 68 16s
Relative Abundance
1.00
0.75
0.50
0.25
0.00
103
124
152
93
Sample
class
Actinobacteria
Bacilli
Bacteroidia
Betaproteobacteria
Clostridia
Fusobacteriia
Gammaproteobacteria
Other
unclassified_Bacteria
Verrucomicrobiae
Patient: 68
Percentage
100
75
50
25
0
0
10
20
Day
GO
actin filament severing
actin nucleation
arginine catabolic process to ornithine
barbed−end actin filament capping
biosynthetic process
carbohydrate metabolic process
cell redox homeostasis
cellular amino acid metabolic process
cellular iron ion homeostasis
epidermis development
gluconeogenesis
glucose metabolic process
glycerol ether metabolic process
glycolytic process
hyaluronan metabolic process
immune response
immunoglobulin production
keratinocyte activation
keratinocyte proliferation
L−alanine catabolic process
metabolic process
phosphoenolpyruvate−dependent sugar phosphotransferase system
protein folding
regulation of transcription, DNA−templated
response to stress
transcription, DNA−templated
translation
transmembrane transport
tricarboxylic acid cycle

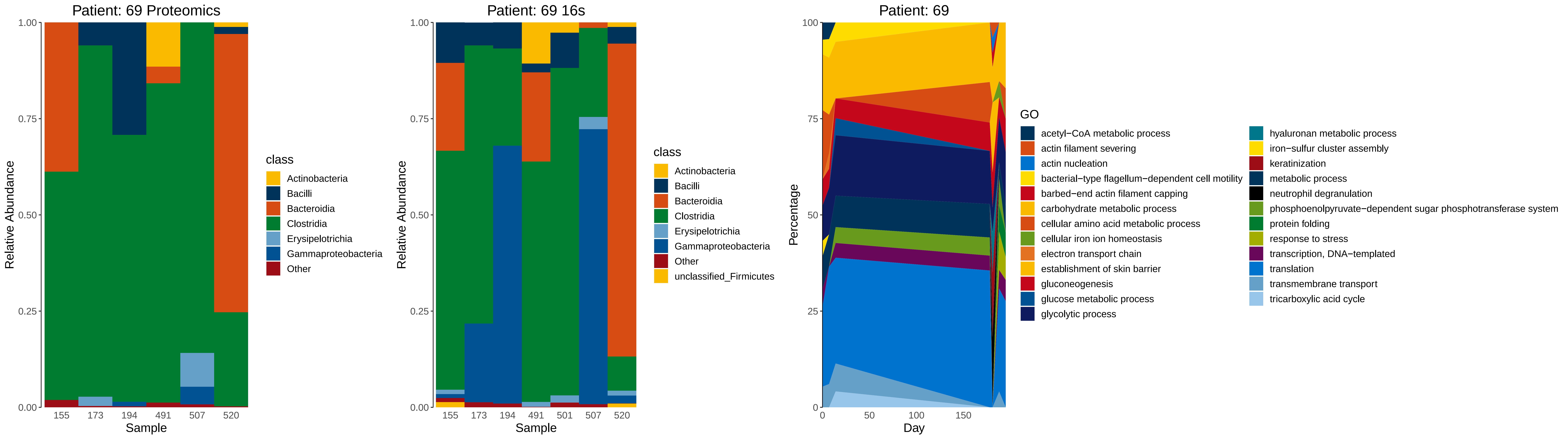
Patient: 69 Proteomics
Relative Abundance
1.00
0.75
0.50
0.25
0.00
155
173
194
491
507
520
Sample
class
Actinobacteria
Bacilli
Bacteroidia
Clostridia
Erysipelotrichia
Gammaproteobacteria
Other
Patient: 69 16s
Relative Abundance
1.00
0.75
0.50
0.25
0.00
155
173
194
491
501
507
520
Sample
class
Actinobacteria
Bacilli
Bacteroidia
Clostridia
Erysipelotrichia
Gammaproteobacteria
Other
unclassified_Firmicutes
Patient: 69
Percentage
100
75
50
25
0
0
50
100
150
Day
GO
acetyl−CoA metabolic process
actin filament severing
actin nucleation
bacterial−type flagellum−dependent cell motility
barbed−end actin filament capping
carbohydrate metabolic process
cellular amino acid metabolic process
cellular iron ion homeostasis
electron transport chain
establishment of skin barrier
gluconeogenesis
glucose metabolic process
glycolytic process
hyaluronan metabolic process
iron−sulfur cluster assembly
keratinization
metabolic process
neutrophil degranulation
phosphoenolpyruvate−dependent sugar phosphotransferase system
protein folding
response to stress
transcription, DNA−templated
translation
transmembrane transport
tricarboxylic acid cycle

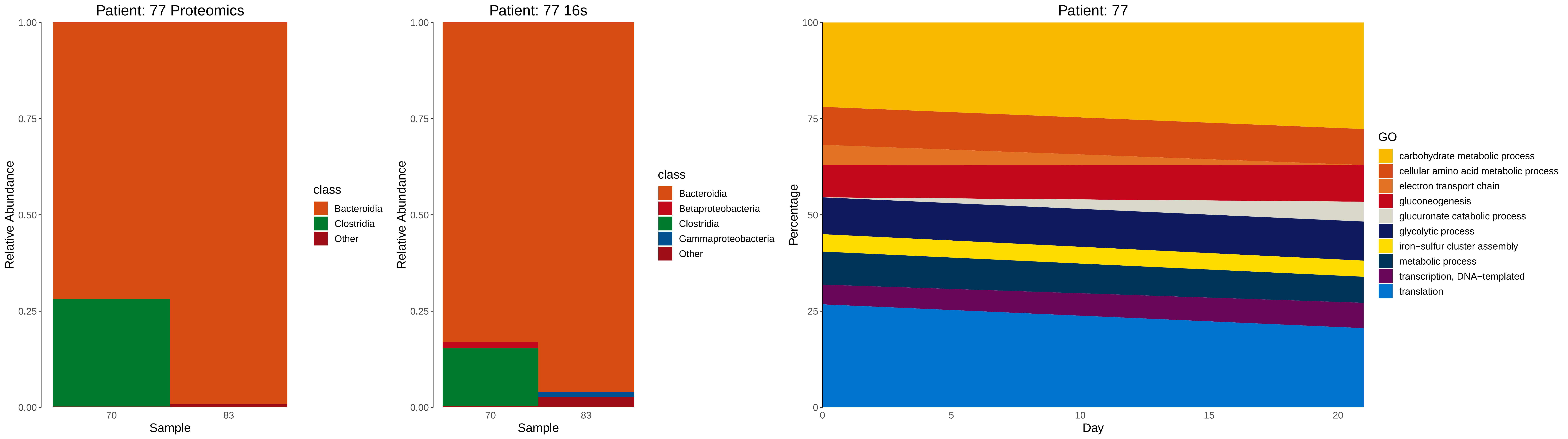

Patient: 77 Proteomics
Relative Abundance
1.00
0.75
0.50
0.25
0.00
70
83
Sample
class
Bacteroidia
Clostridia
Other
Patient: 77 16s
Relative Abundance
1.00
0.75
0.50
0.25
0.00
70
83
Sample
class
Bacteroidia
Betaproteobacteria
Clostridia
Gammaproteobacteria
Other
Patient: 77
Percentage
100
75
50
25
0
0
5
10
15
20
Day
GO
carbohydrate metabolic process
cellular amino acid metabolic process
electron transport chain
gluconeogenesis
glucuronate catabolic process
glycolytic process
iron−sulfur cluster assembly
metabolic process
transcription, DNA−templated
translation

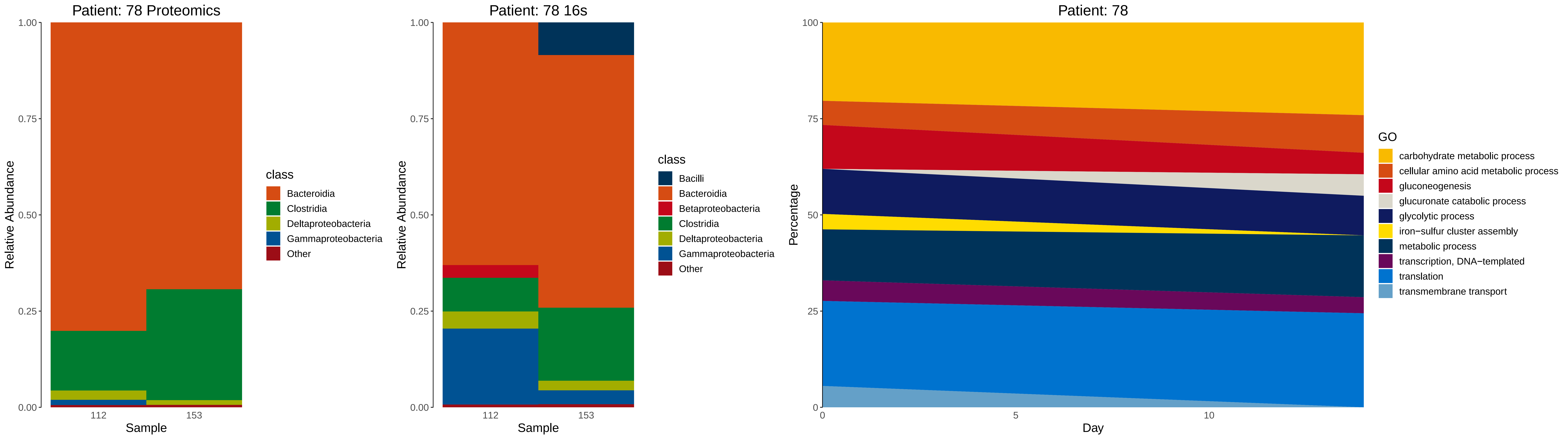

Patient: 78 Proteomics
Relative Abundance
1.00
0.75
0.50
0.25
0.00
112
153
Sample
class
Bacteroidia
Clostridia
Deltaproteobacteria
Gammaproteobacteria
Other
Patient: 78 16s
Relative Abundance
1.00
0.75
0.50
0.25
0.00
112
153
Sample
class
Bacilli
Bacteroidia
Betaproteobacteria
Clostridia
Deltaproteobacteria
Gammaproteobacteria
Other
Patient: 78
Percentage
100
75
50
25
0
0
5
10
Day
GO
carbohydrate metabolic process
cellular amino acid metabolic process
gluconeogenesis
glucuronate catabolic process
glycolytic process
iron−sulfur cluster assembly
metabolic process
transcription, DNA−templated
translation
transmembrane transport

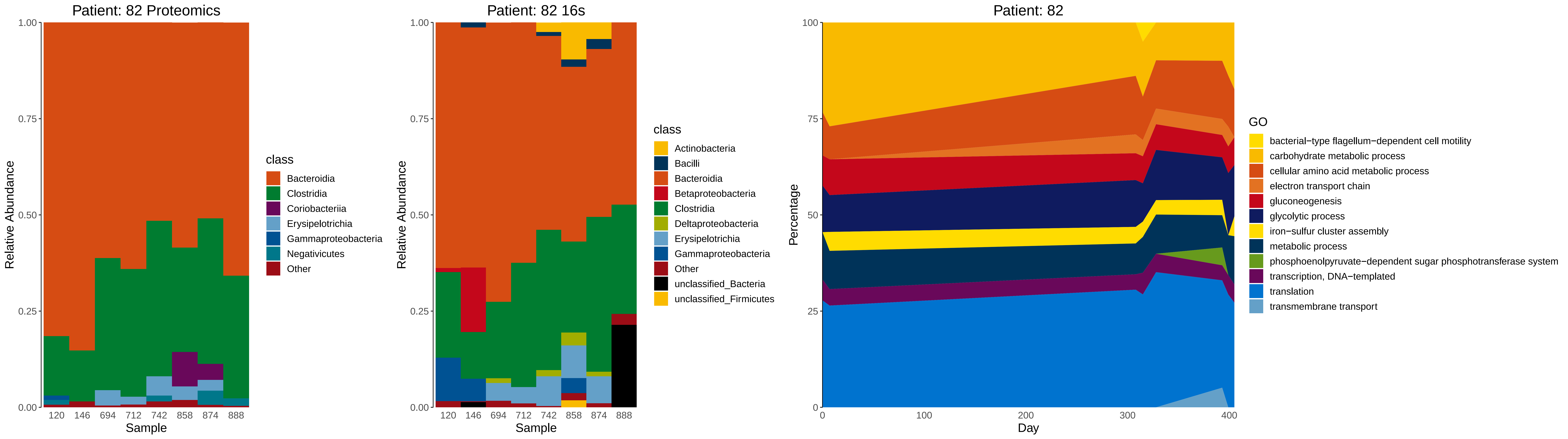

Patient: 82 Proteomics
Relative Abundance
Sample
120
146
694
712
742
858
874
888
class
Bacteroidia
Clostridia
Coriobacteriia
Erysipelotrichia
Gammaproteobacteria
Negativicutes
Other
Patient: 82 16s
class
Actinobacteria
Bacilli
Bacteroidia
Betaproteobacteria
Clostridia
Deltaproteobacteria
Erysipelotrichia
Gammaproteobacteria
Other
unclassified_Bacteria
unclassified_Firmicutes
Patient: 82
Percentage
Day
GO
bacterial−type flagellum−dependent cell motility
carbohydrate metabolic process
cellular amino acid metabolic process
electron transport chain
gluconeogenesis
glycolytic process
iron−sulfur cluster assembly
metabolic process
phosphoenolpyruvate−dependent sugar phosphotransferase system
transcription, DNA−templated
translation
transmembrane transport

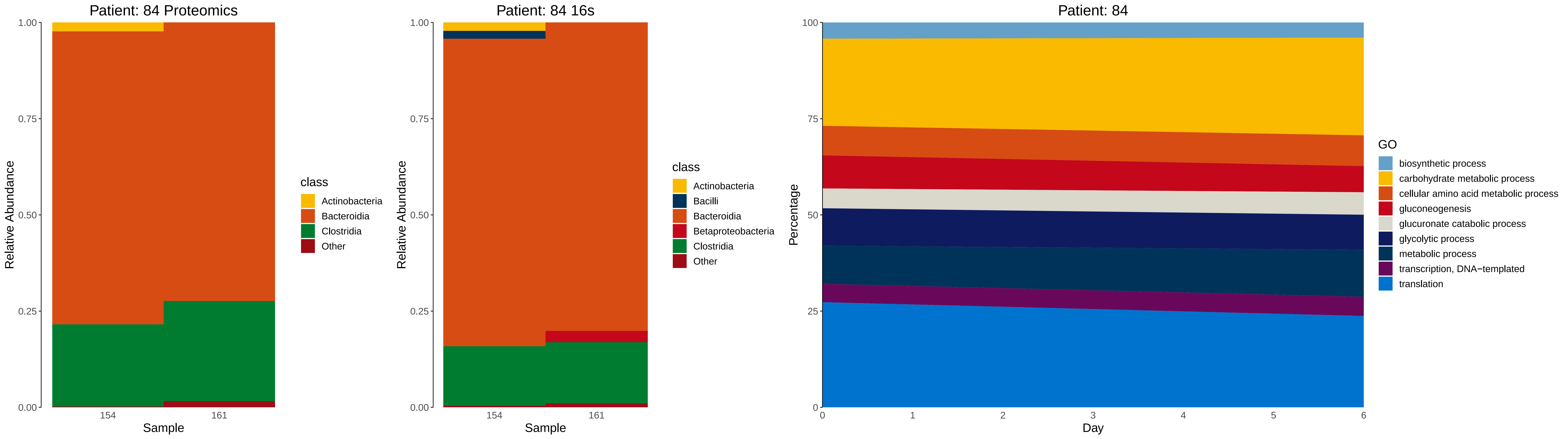

Patient: 84 Proteomics
Relative Abundance
1.00
0.75
0.50
0.25
0.00
154
161
Sample
class
Actinobacteria
Bacteroidia
Clostridia
Other
Patient: 84 16s
Relative Abundance
154
161
Sample
class
Actinobacteria
Bacilli
Bacteroidia
Betaproteobacteria
Clostridia
Other
Patient: 84
Percentage
100
75
50
25
0
0
1
2
3
4
5
6
Day
GO
biosynthetic process
carbohydrate metabolic process
cellular amino acid metabolic process
gluconeogenesis
glucuronate catabolic process
glycolytic process
metabolic process
transcription, DNA−templated
translation

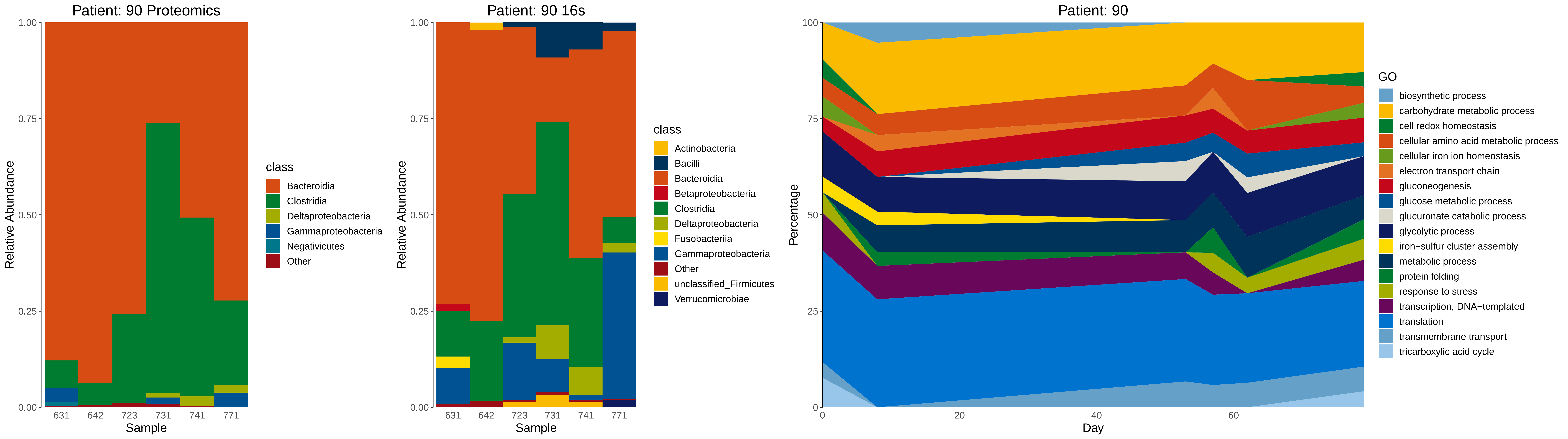
Patient: 90 Proteomics
Relative Abundance
1.00
0.75
0.50
0.25
0.00
631
642
723
731
741
771
Sample
class
Bacteroidia
Clostridia
Deltaproteobacteria
Gammaproteobacteria
Negativicutes
Other
Patient: 90 16s
Relative Abundance
Sample
class
Actinobacteria
Bacilli
Bacteroidia
Betaproteobacteria
Clostridia
Deltaproteobacteria
Fusobacteriia
Gammaproteobacteria
Other
unclassified_Firmicutes
Verrucomicrobiae
Patient: 90
Percentage
100
75
50
25
0
0
20
40
60
Day
GO
biosynthetic process
carbohydrate metabolic process
cell redox homeostasis
cellular amino acid metabolic process
cellular iron ion homeostasis
electron transport chain
gluconeogenesis
glucose metabolic process
glucuronate catabolic process
glycolytic process
iron−sulfur cluster assembly
metabolic process
protein folding
response to stress
transcription, DNA−templated
translation
transmembrane transport
tricarboxylic acid cycle

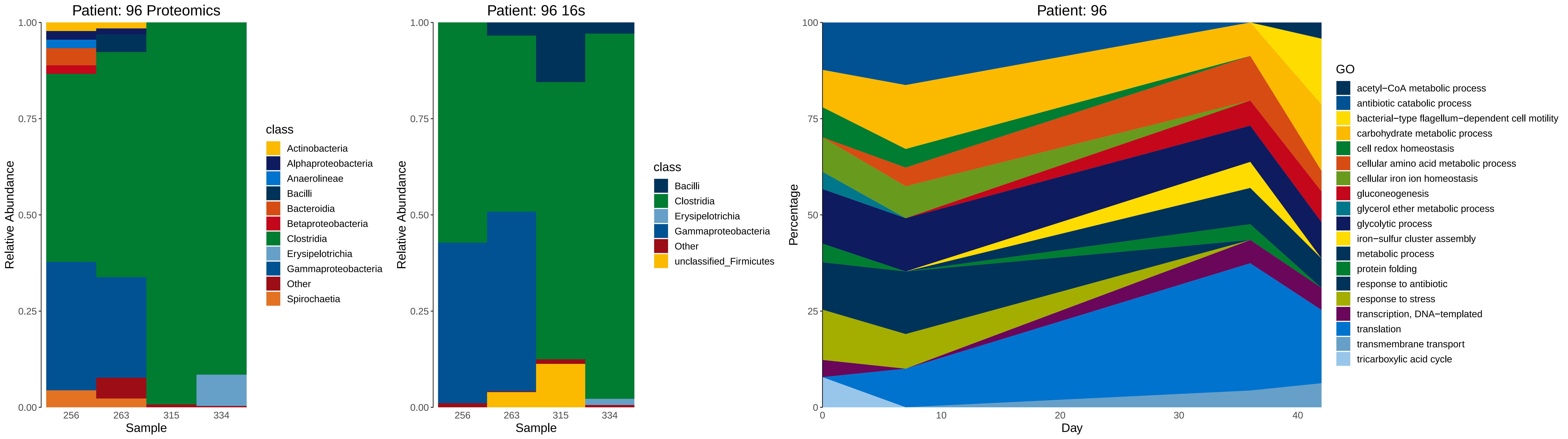

Patient: 96 Proteomics
Relative Abundance
1.00
0.75
0.50
0.25
0.00
256
263
315
334
Sample
class
Actinobacteria
Alphaproteobacteria
Anaerolineae
Bacilli
Bacteroidia
Betaproteobacteria
Clostridia
Erysipelotrichia
Gammaproteobacteria
Other
Spirochaetia
Patient: 96 16s
Relative Abundance
1.00
0.75
0.50
0.25
0.00
256
263
315
334
Sample
class
Bacilli
Clostridia
Erysipelotrichia
Gammaproteobacteria
Other
unclassified_Firmicutes
Patient: 96
Percentage
100
75
50
25
0
0
10
20
30
40
Day
GO
acetyl−CoA metabolic process
antibiotic catabolic process
bacterial−type flagellum−dependent cell motility
carbohydrate metabolic process
cell redox homeostasis
cellular amino acid metabolic process
cellular iron ion homeostasis
gluconeogenesis
glycerol ether metabolic process
glycolytic process
iron−sulfur cluster assembly
metabolic process
protein folding
response to antibiotic
response to stress
transcription, DNA−templated
translation
transmembrane transport
tricarboxylic acid cycle

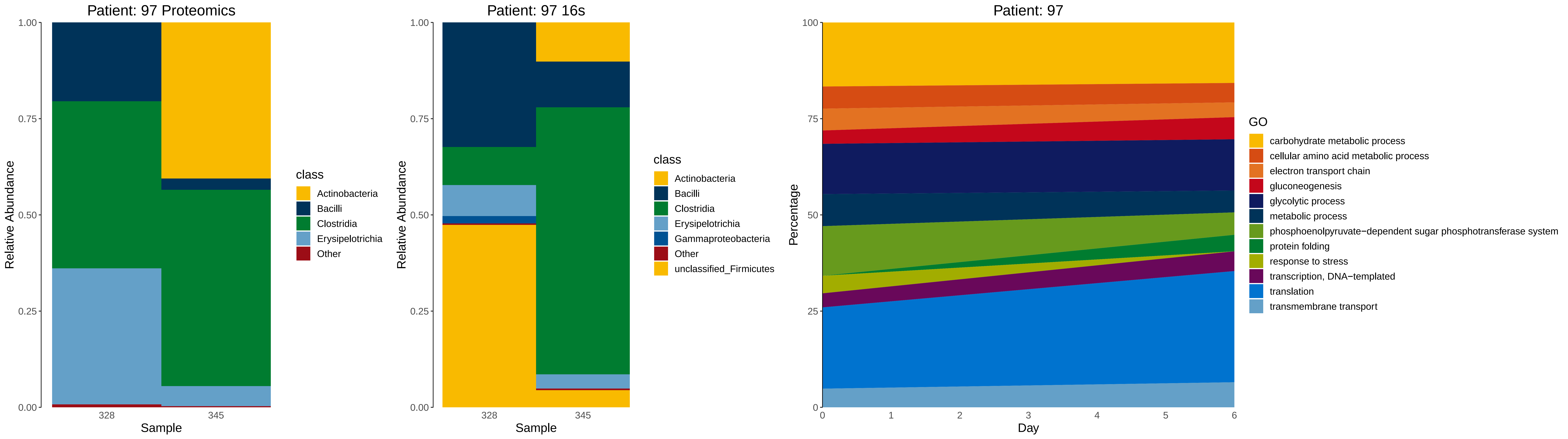

Patient: 97 Proteomics
Relative Abundance
1.00
0.75
0.50
0.25
0.00
328
345
Sample
class
Actinobacteria
Bacilli
Clostridia
Erysipelotrichia
Other
Patient: 97 16s
Relative Abundance
1.00
0.75
0.50
0.25
0.00
328
345
Sample
class
Actinobacteria
Bacilli
Clostridia
Erysipelotrichia
Gammaproteobacteria
Other
unclassified_Firmicutes
Patient: 97
Percentage
100
75
50
25
0
0
1
2
3
4
5
6
Day
GO
carbohydrate metabolic process
cellular amino acid metabolic process
electron transport chain
gluconeogenesis
glycolytic process
metabolic process
phosphoenolpyruvate−dependent sugar phosphotransferase system
protein folding
response to stress
transcription, DNA−templated
translation
transmembrane transport

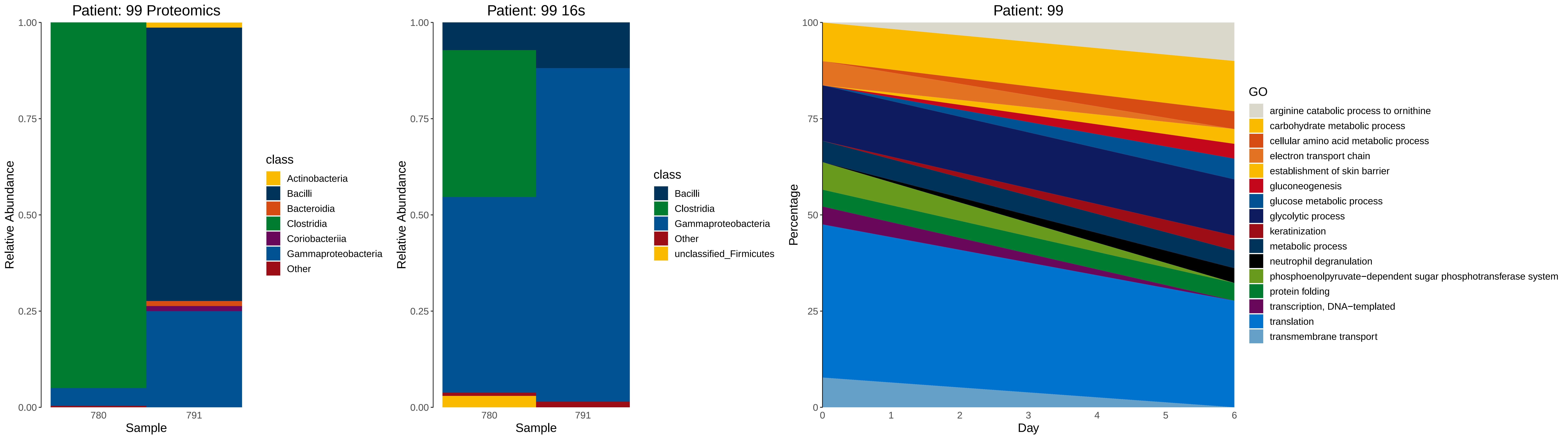

Patient: 99 Proteomics
Relative Abundance
1.00
0.75
0.50
0.25
0.00
780
791
Sample
class
Actinobacteria
Bacilli
Bacteroidia
Clostridia
Coriobacteriia
Gammaproteobacteria
Other
Patient: 99 16s
Relative Abundance
780
791
Sample
class
Bacilli
Clostridia
Gammaproteobacteria
Other
unclassified_Firmicutes
Patient: 99
Percentage
100
75
50
25
0
0 1 2 3 4 5 6
Day
GO
arginine catabolic process to ornithine
carbohydrate metabolic process
cellular amino acid metabolic process
electron transport chain
establishment of skin barrier
gluconeogenesis
glucose metabolic process
glycolytic process
keratinization
metabolic process
neutrophil degranulation
phosphoenolpyruvate−dependent sugar phosphotransferase system
protein folding
transcription, DNA−templated
translation
transmembrane transport

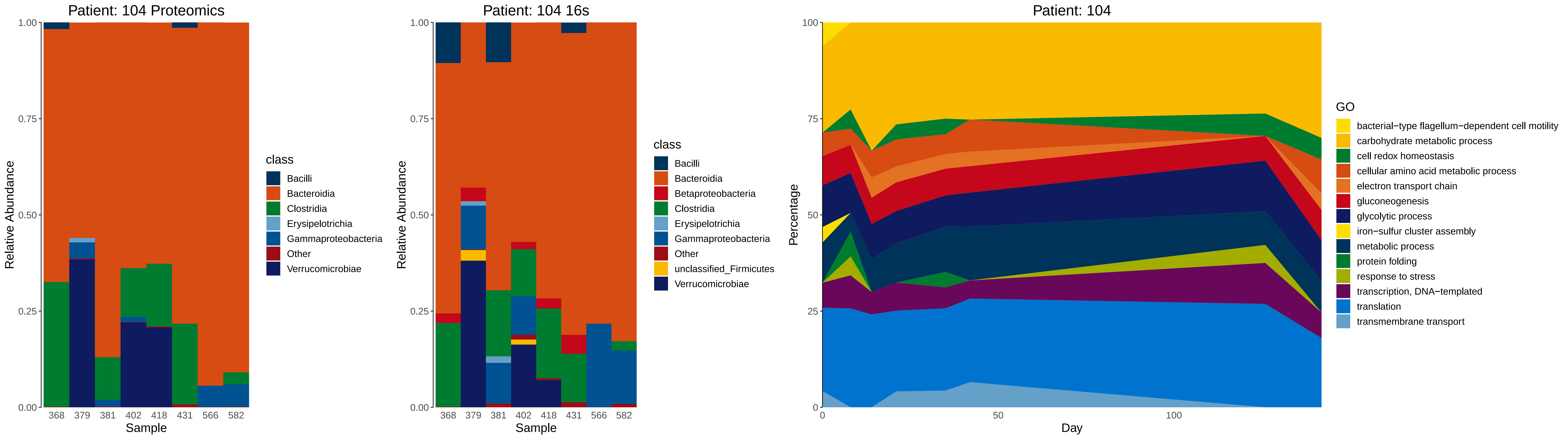
Patient: 104 Proteomics
Relative Abundance
Sample
368
379
381
402
418
431
566
582
class
Bacilli
Bacteroidia
Clostridia
Erysipelotrichia
Gammaproteobacteria
Other
Verrucomicrobiae
Patient: 104 16s
class
Bacilli
Bacteroidia
Betaproteobacteria
Clostridia
Erysipelotrichia
Gammaproteobacteria
Other
unclassified_Firmicutes
Verrucomicrobiae
Patient: 104
Percentage
Day
GO
bacterial−type flagellum−dependent cell motility
carbohydrate metabolic process
cell redox homeostasis
cellular amino acid metabolic process
electron transport chain
gluconeogenesis
glycolytic process
iron−sulfur cluster assembly
metabolic process
protein folding
response to stress
transcription, DNA−templated
translation
transmembrane transport

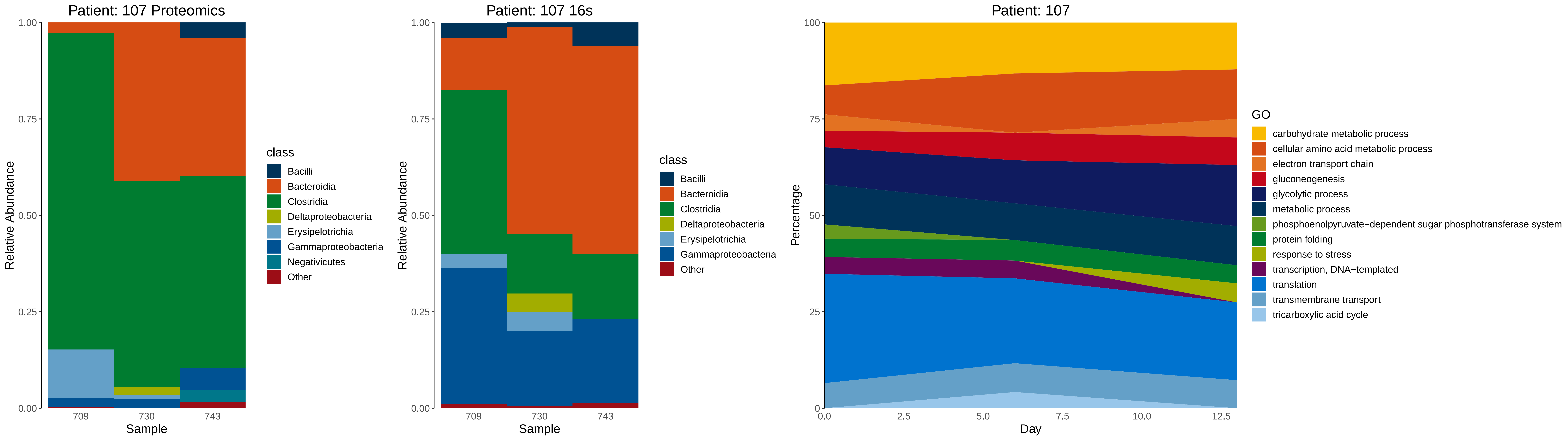

Patient: 107 Proteomics
Relative Abundance
1.00
0.75
0.50
0.25
0.00
709
730
743
Sample
class
Bacilli
Bacteroidia
Clostridia
Deltaproteobacteria
Erysipelotrichia
Gammaproteobacteria
Negativicutes
Other
Patient: 107 16s
Relative Abundance
Sample
class
Bacilli
Bacteroidia
Clostridia
Deltaproteobacteria
Erysipelotrichia
Gammaproteobacteria
Other
Patient: 107
Percentage
100
75
50
25
0
0.0
2.5
5.0
7.5
10.0
12.5
Day
GO
carbohydrate metabolic process
cellular amino acid metabolic process
electron transport chain
gluconeogenesis
glycolytic process
metabolic process
phosphoenolpyruvate−dependent sugar phosphotransferase system
protein folding
response to stress
transcription, DNA−templated
translation
transmembrane transport
tricarboxylic acid cycle

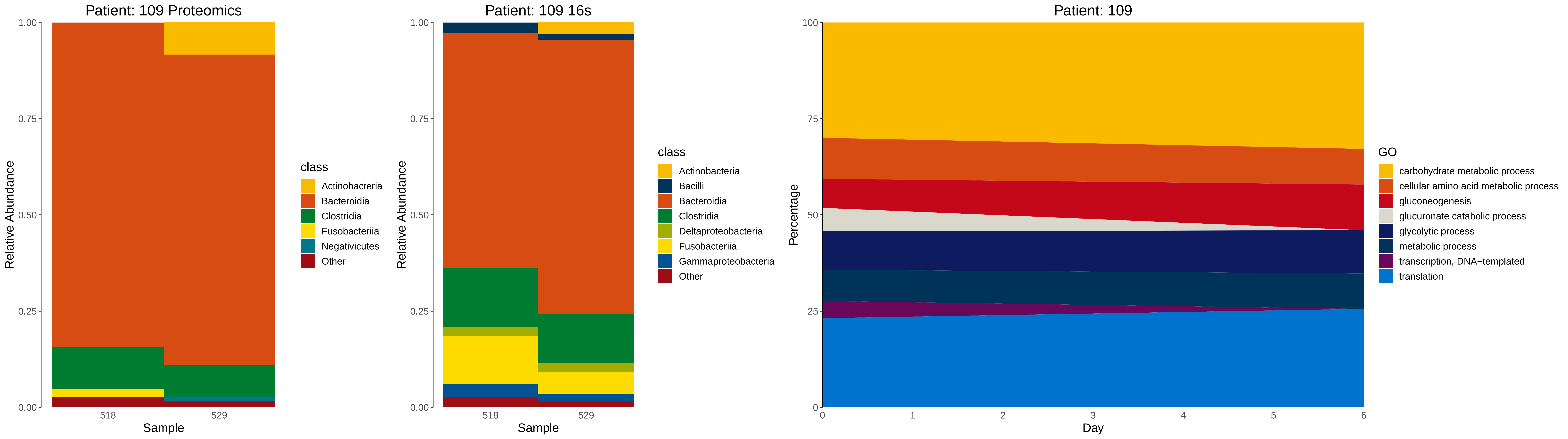
Patient: 109 Proteomics
Relative Abundance
1.00
0.75
0.50
0.25
0.00
518
529
Sample
class
Actinobacteria
Bacteroidia
Clostridia
Fusobacteriia
Negativicutes
Other
Patient: 109 16s
Relative Abundance
Sample
class
Actinobacteria
Bacilli
Bacteroidia
Clostridia
Deltaproteobacteria
Fusobacteriia
Gammaproteobacteria
Other
Patient: 109
Percentage
100
75
50
25
0
0
1
2
3
4
5
6
Day
GO
carbohydrate metabolic process
cellular amino acid metabolic process
gluconeogenesis
glucuronate catabolic process
glycolytic process
metabolic process
transcription, DNA−templated
translation

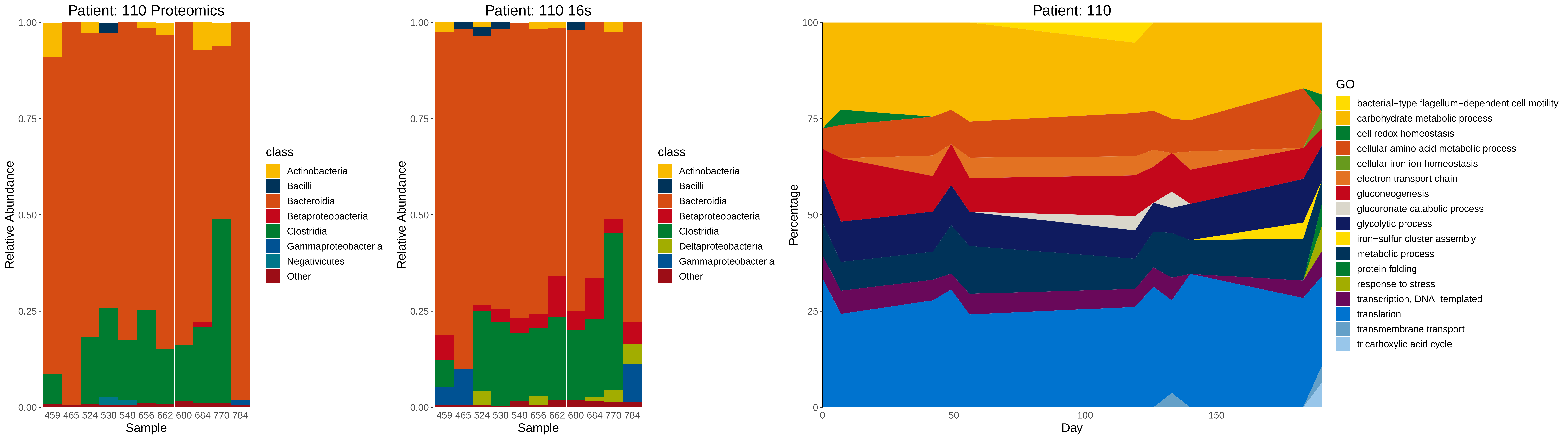
Patient: 110 Proteomics
Relative Abundance
Sample
459 465 524 538 548 656 662 680 684 770 784
class
Actinobacteria
Bacilli
Bacteroidia
Betaproteobacteria
Clostridia
Gammaproteobacteria
Negativicutes
Other
Patient: 110 16s
Deltaproteobacteria
Patient: 110
Percentage
Day
GO
bacterial−type flagellum−dependent cell motility
carbohydrate metabolic process
cell redox homeostasis
cellular amino acid metabolic process
cellular iron ion homeostasis
electron transport chain
gluconeogenesis
glucuronate catabolic process
glycolytic process
iron−sulfur cluster assembly
metabolic process
protein folding
response to stress
transcription, DNA−templated
translation
transmembrane transport
tricarboxylic acid cycle

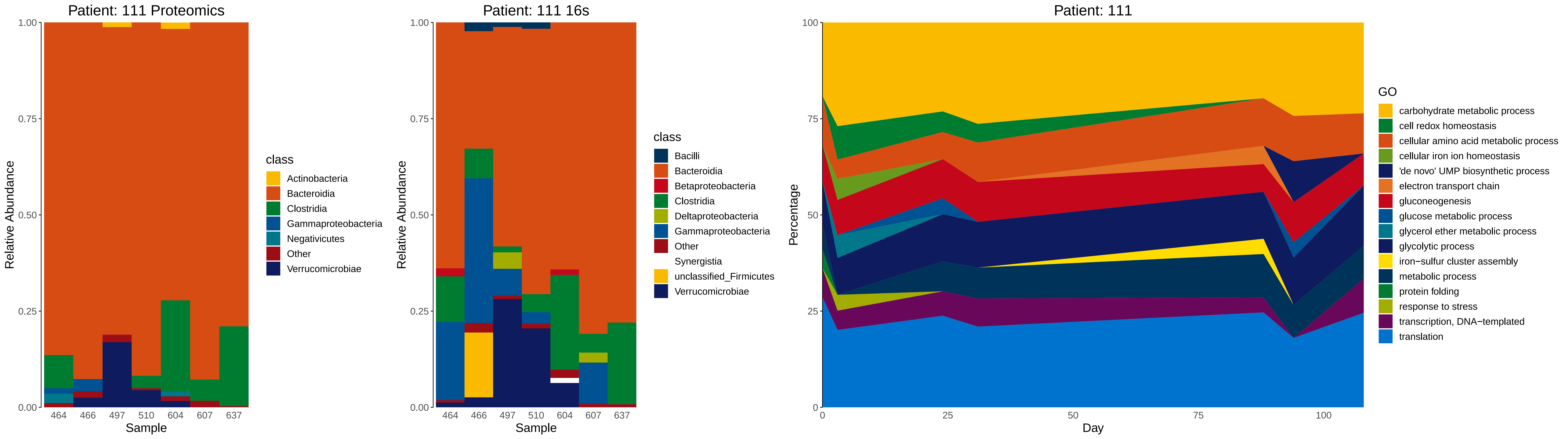

Patient: 111 Proteomics
Relative Abundance
1.00
0.75
0.50
0.25
0.00
464
466
497
510
604
607
637
Sample
class
Actinobacteria
Bacteroidia
Clostridia
Gammaproteobacteria
Negativicutes
Other
Verrucomicrobiae
Patient: 111 16s
Relative Abundance
1.00
0.75
0.50
0.25
0.00
464
466
497
510
604
607
637
Sample
class
Bacilli
Bacteroidia
Betaproteobacteria
Clostridia
Deltaproteobacteria
Gammaproteobacteria
Other
Synergistia
unclassified_Firmicutes
Verrucomicrobiae
Patient: 111
Percentage
100
75
50
25
0
0
25
50
75
100
Day
GO
carbohydrate metabolic process
cell redox homeostasis
cellular amino acid metabolic process
cellular iron ion homeostasis
'de novo' UMP biosynthetic process
electron transport chain
gluconeogenesis
glucose metabolic process
glycerol ether metabolic process
glycolytic process
iron−sulfur cluster assembly
metabolic process
protein folding
response to stress
transcription, DNA−templated
translation

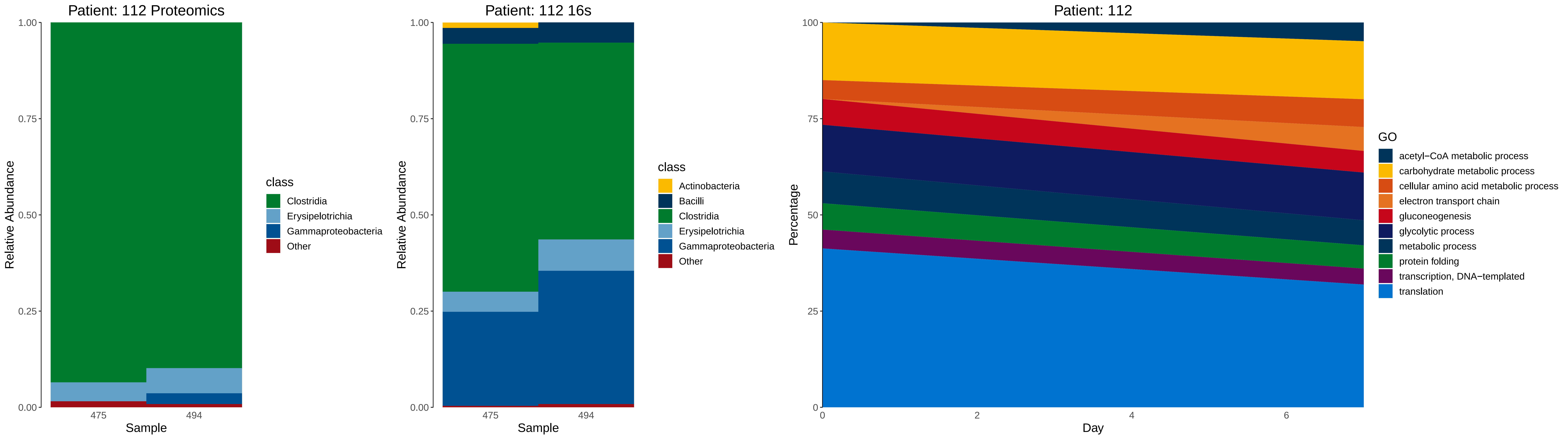

Patient: 112 Proteomics
Relative Abundance
1.00
0.75
0.50
0.25
0.00
475
494
Sample
class
Clostridia
Erysipelotrichia
Gammaproteobacteria
Other
Patient: 112 16s
Relative Abundance
1.00
0.75
0.50
0.25
0.00
475
494
Sample
class
Actinobacteria
Bacilli
Clostridia
Erysipelotrichia
Gammaproteobacteria
Other
Patient: 112
Percentage
100
75
50
25
0
0
2
4
6
Day
GO
acetyl−CoA metabolic process
carbohydrate metabolic process
cellular amino acid metabolic process
electron transport chain
gluconeogenesis
glycolytic process
metabolic process
protein folding
transcription, DNA−templated
translation

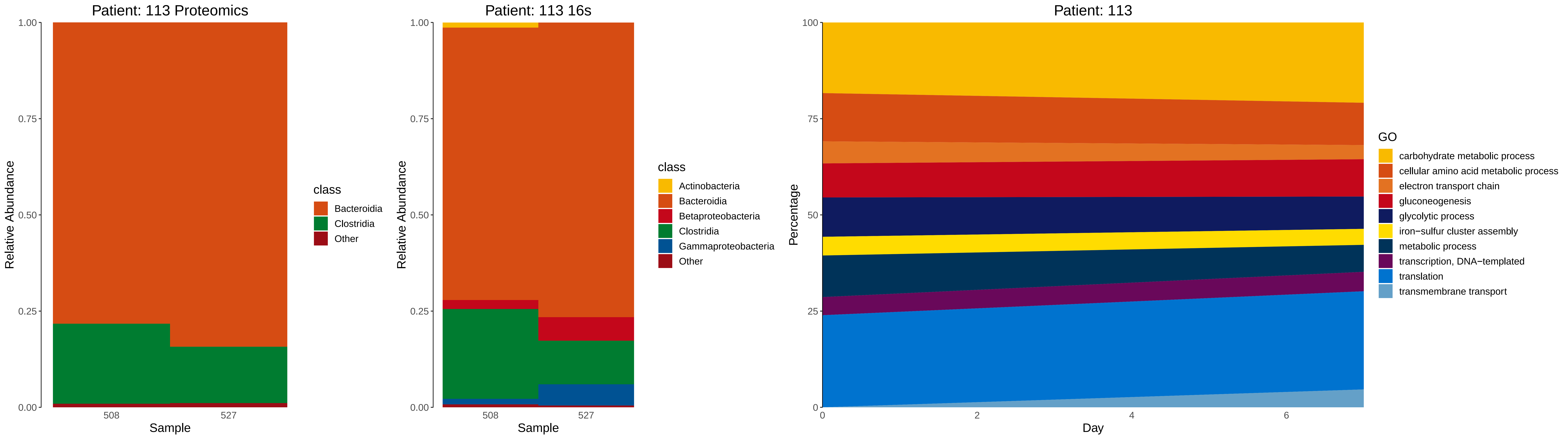

Patient: 113 Proteomics
Relative Abundance
1.00
0.75
0.50
0.25
0.00
508
527
Sample
class
Bacteroidia
Clostridia
Other
Patient: 113 16s
Relative Abundance
Sample
class
Actinobacteria
Bacteroidia
Betaproteobacteria
Clostridia
Gammaproteobacteria
Other
Patient: 113
Percentage
100
75
50
25
0
0
2
4
6
Day
GO
carbohydrate metabolic process
cellular amino acid metabolic process
electron transport chain
gluconeogenesis
glycolytic process
iron−sulfur cluster assembly
metabolic process
transcription, DNA−templated
translation
transmembrane transport

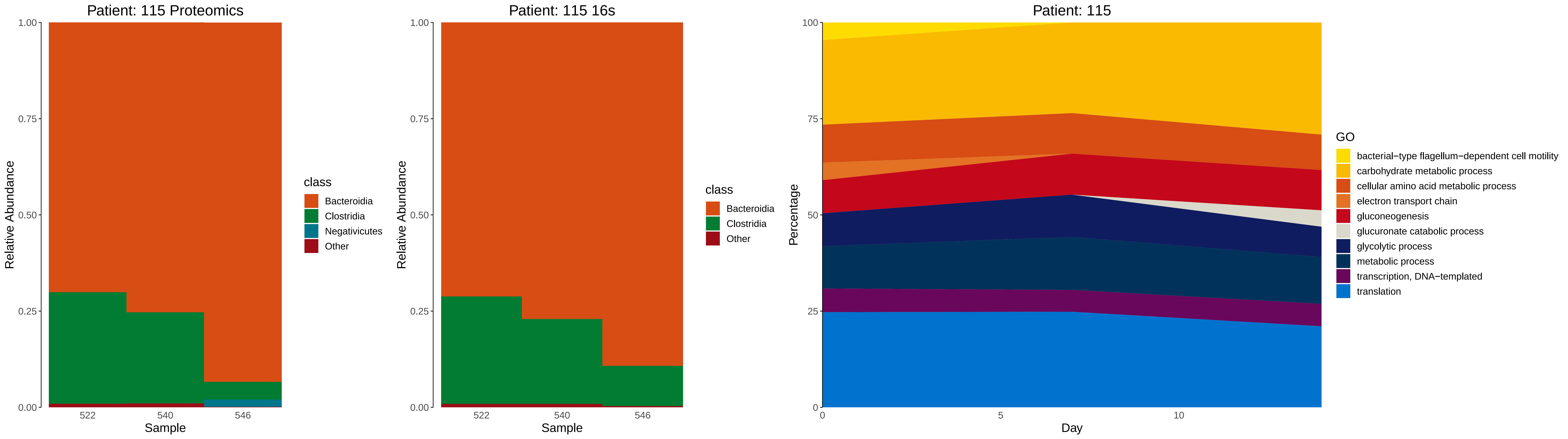

Patient: 115 Proteomics
Relative Abundance
1.00
0.75
0.50
0.25
0.00
522
540
546
Sample
class
Bacteroidia
Clostridia
Negativicutes
Other
Patient: 115 16s
Relative Abundance
1.00
0.75
0.50
0.25
0.00
522
540
546
Sample
class
Bacteroidia
Clostridia
Other
Patient: 115
Percentage
100
75
50
25
0
0
5
10
Day
GO
bacterial−type flagellum−dependent cell motility
carbohydrate metabolic process
cellular amino acid metabolic process
electron transport chain
gluconeogenesis
glucuronate catabolic process
glycolytic process
metabolic process
transcription, DNA−templated
translation

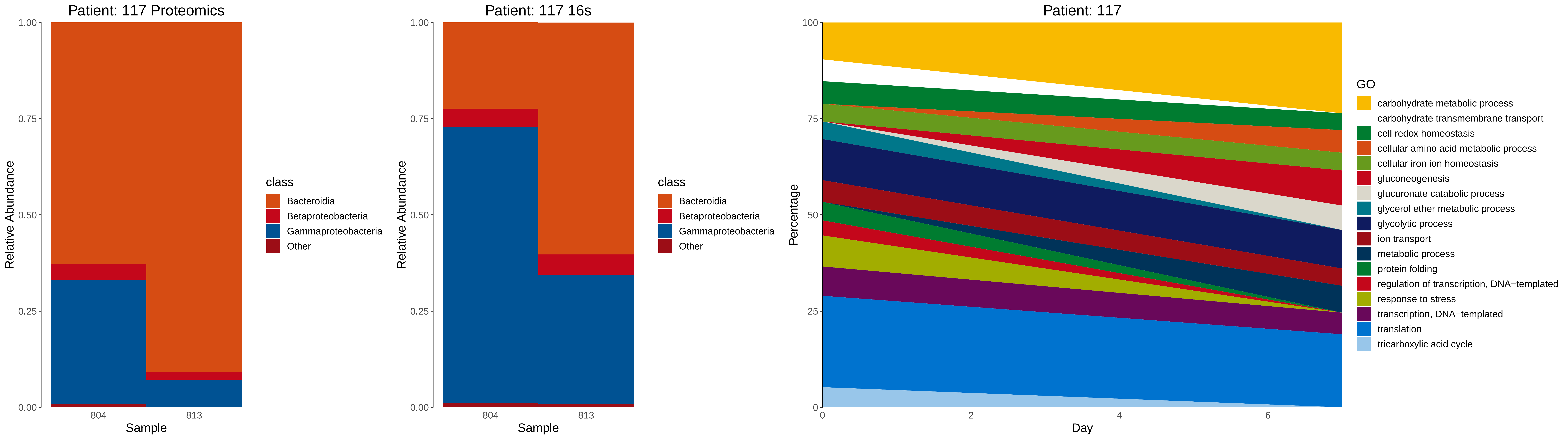
Patient: 117 Proteomics
Relative Abundance
1.00
0.75
0.50
0.25
0.00
804
813
Sample
class
Bacteroidia
Betaproteobacteria
Gammaproteobacteria
Other
Patient: 117 16s
Relative Abundance
804
813
Sample
Patient: 117
Percentage
100
75
50
25
0
0
2
4
6
Day
GO
carbohydrate metabolic process
carbohydrate transmembrane transport
cell redox homeostasis
cellular amino acid metabolic process
cellular iron ion homeostasis
gluconeogenesis
glucuronate catabolic process
glycerol ether metabolic process
glycolytic process
ion transport
metabolic process
protein folding
regulation of transcription, DNA−templated
response to stress
transcription, DNA−templated
translation
tricarboxylic acid cycle

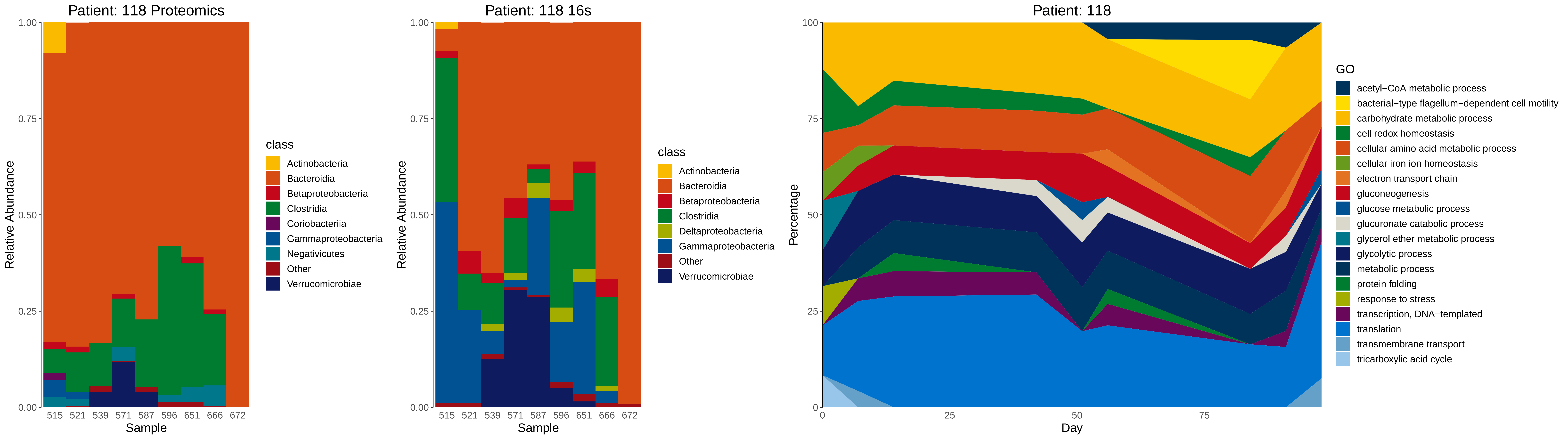

Patient: 118 Proteomics
Relative Abundance
Sample
515 521 539 571 587 596 651 666 672
class
Actinobacteria
Bacteroidia
Betaproteobacteria
Clostridia
Coriobacteriia
Gammaproteobacteria
Negativicutes
Other
Verrucomicrobiae
Patient: 118 16s
Relative Abundance
Sample
class
Actinobacteria
Bacteroidia
Betaproteobacteria
Clostridia
Deltaproteobacteria
Gammaproteobacteria
Other
Verrucomicrobiae
Patient: 118
Percentage
Day
GO
acetyl−CoA metabolic process
bacterial−type flagellum−dependent cell motility
carbohydrate metabolic process
cell redox homeostasis
cellular amino acid metabolic process
cellular iron ion homeostasis
electron transport chain
gluconeogenesis
glucose metabolic process
glucuronate catabolic process
glycerol ether metabolic process
glycolytic process
metabolic process
protein folding
response to stress
transcription, DNA−templated
translation
transmembrane transport
tricarboxylic acid cycle

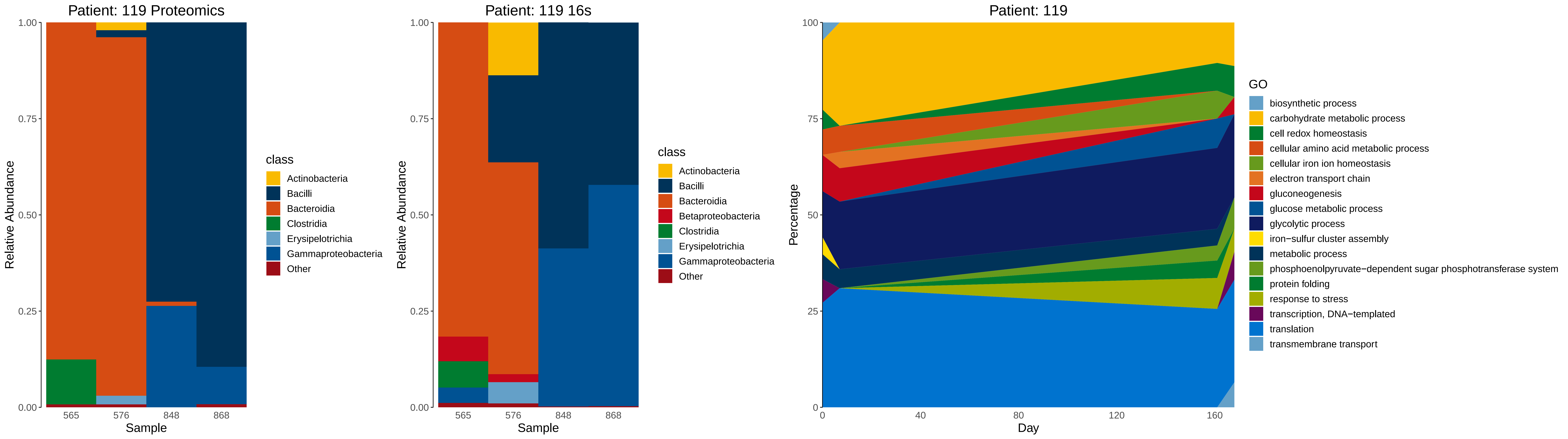

Patient: 119 Proteomics
Relative Abundance
1.00
0.75
0.50
0.25
0.00
565
576
848
868
Sample
class
Actinobacteria
Bacilli
Bacteroidia
Clostridia
Erysipelotrichia
Gammaproteobacteria
Other
Patient: 119 16s
Relative Abundance
1.00
0.75
0.50
0.25
0.00
565
576
848
868
Sample
class
Actinobacteria
Bacilli
Bacteroidia
Betaproteobacteria
Clostridia
Erysipelotrichia
Gammaproteobacteria
Other
Patient: 119
Percentage
100
75
50
25
0
0
40
80
120
160
Day
GO
biosynthetic process
carbohydrate metabolic process
cell redox homeostasis
cellular amino acid metabolic process
cellular iron ion homeostasis
electron transport chain
gluconeogenesis
glucose metabolic process
glycolytic process
iron−sulfur cluster assembly
metabolic process
phosphoenolpyruvate−dependent sugar phosphotransferase system
protein folding
response to stress
transcription, DNA−templated
translation
transmembrane transport

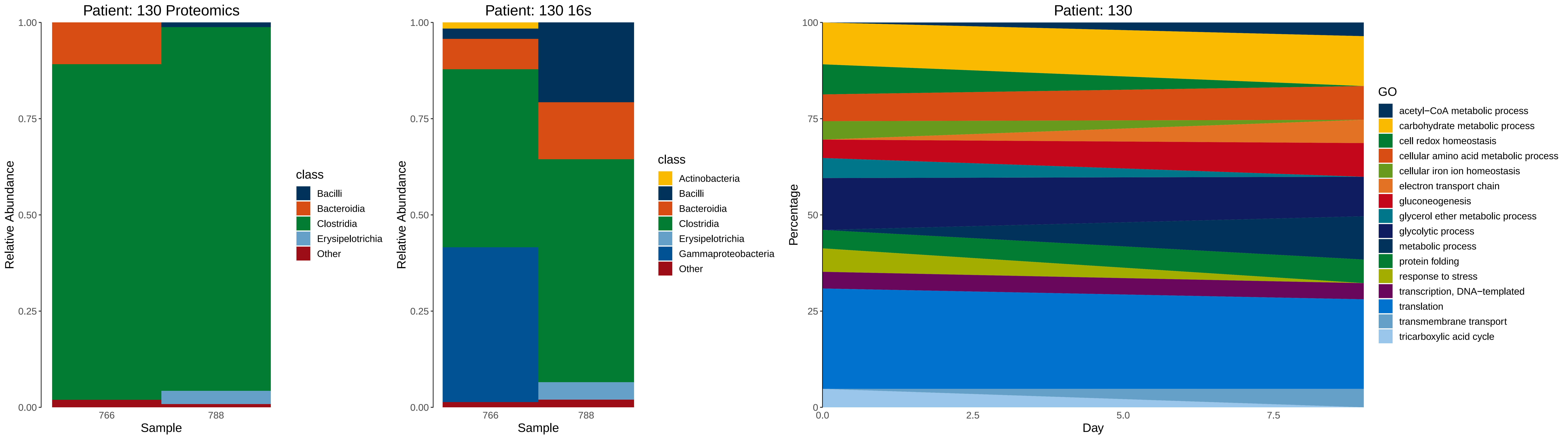

Patient: 130 Proteomics
Relative Abundance
Sample
766
788
1.00
0.75
0.50
0.25
0.00
class
Bacilli
Bacteroidia
Clostridia
Erysipelotrichia
Other
Patient: 130 16s
Relative Abundance
Sample
766
788
class
Actinobacteria
Bacilli
Bacteroidia
Clostridia
Erysipelotrichia
Gammaproteobacteria
Other
Patient: 130
Percentage
Day
100
75
50
25
0
0.0
2.5
5.0
7.5
GO
acetyl−CoA metabolic process
carbohydrate metabolic process
cell redox homeostasis
cellular amino acid metabolic process
cellular iron ion homeostasis
electron transport chain
gluconeogenesis
glycerol ether metabolic process
glycolytic process
metabolic process
protein folding
response to stress
transcription, DNA−templated
translation
transmembrane transport
tricarboxylic acid cycle

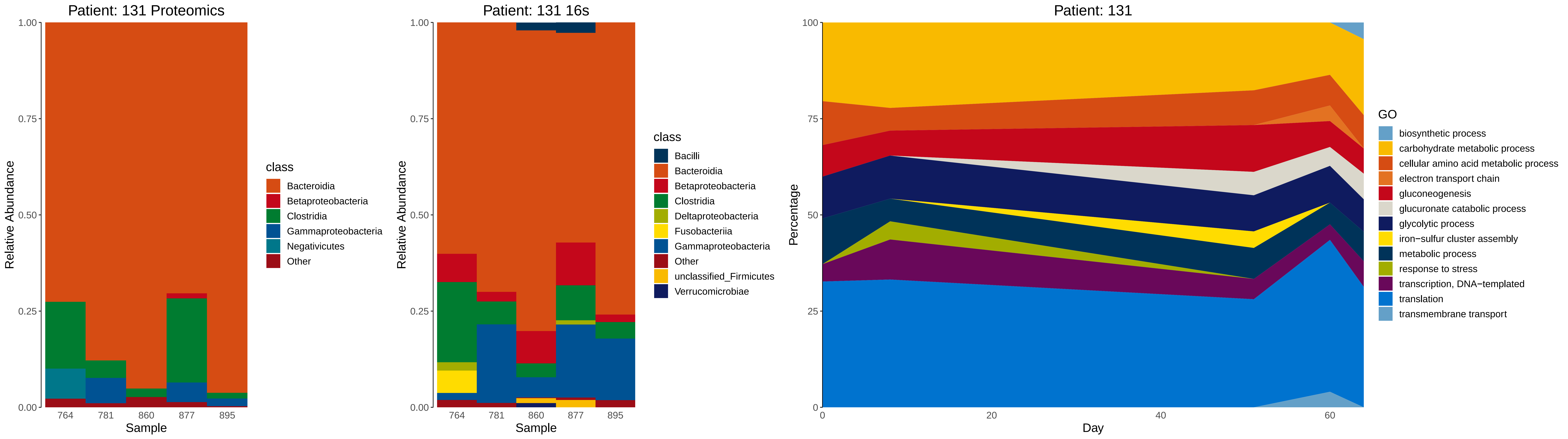
Patient: 131 Proteomics
Relative Abundance
Sample
764
781
860
877
895
0.00
0.25
0.50
0.75
1.00
class
Bacteroidia
Betaproteobacteria
Clostridia
Gammaproteobacteria
Negativicutes
Other
Patient: 131 16s
class
Bacilli
Bacteroidia
Betaproteobacteria
Clostridia
Deltaproteobacteria
Fusobacteriia
Gammaproteobacteria
Other
unclassified_Firmicutes
Verrucomicrobiae
Patient: 131
Percentage
Day
0
20
40
60
25
50
75
100
GO
biosynthetic process
carbohydrate metabolic process
cellular amino acid metabolic process
electron transport chain
gluconeogenesis
glucuronate catabolic process
glycolytic process
iron−sulfur cluster assembly
metabolic process
response to stress
transcription, DNA−templated
translation
transmembrane transport

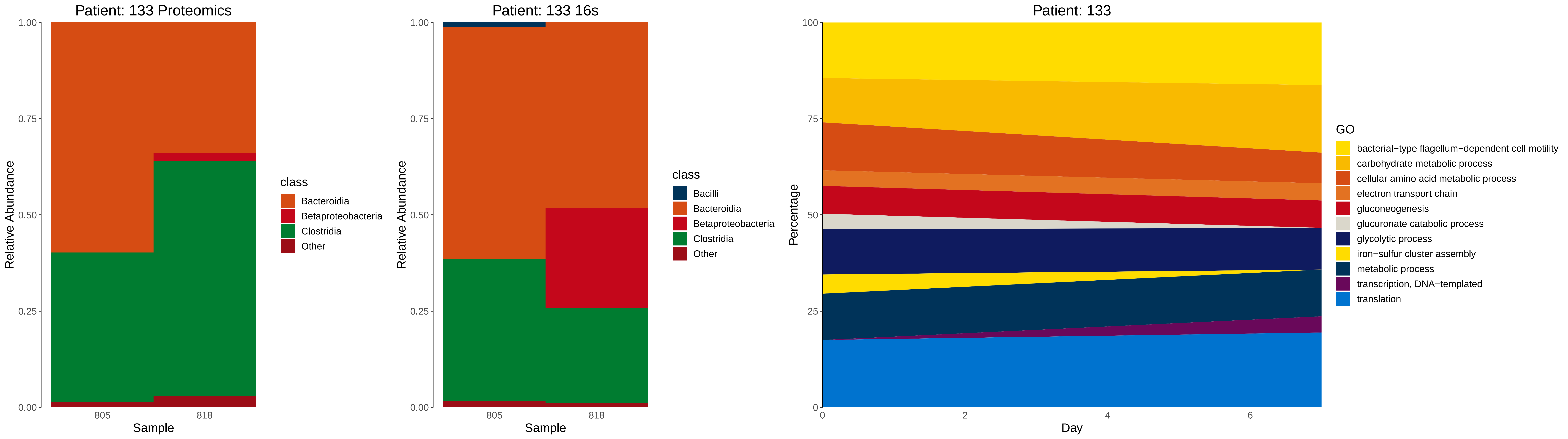

Patient: 133 Proteomics
Relative Abundance
1.00
0.75
0.50
0.25
0.00
805
818
Sample
class
Bacteroidia
Betaproteobacteria
Clostridia
Other
Patient: 133 16s
Relative Abundance
1.00
0.75
0.50
0.25
0.00
805
818
Sample
class
Bacilli
Bacteroidia
Betaproteobacteria
Clostridia
Other
Patient: 133
Percentage
100
75
50
25
0
0
2
4
6
Day
GO
bacterial−type flagellum−dependent cell motility
carbohydrate metabolic process
cellular amino acid metabolic process
electron transport chain
gluconeogenesis
glucuronate catabolic process
glycolytic process
iron−sulfur cluster assembly
metabolic process
transcription, DNA−templated
translation

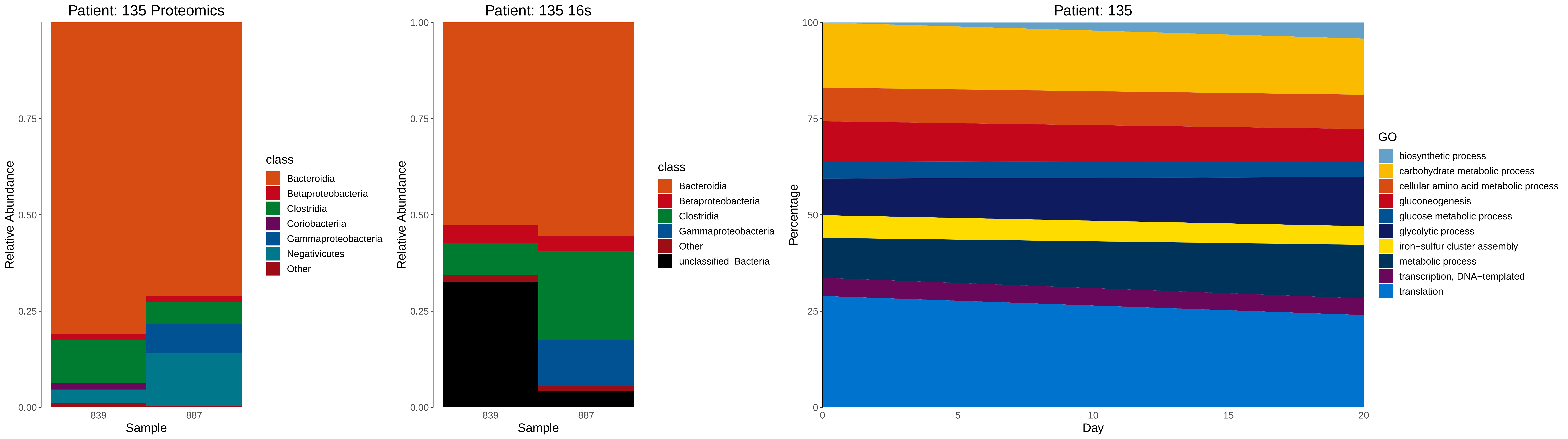
Patient: 135 Proteomics
Relative Abundance
0.00
0.25
0.50
0.75
839
887
Sample
class
Bacteroidia
Betaproteobacteria
Clostridia
Coriobacteriia
Gammaproteobacteria
Negativicutes
Other
Patient: 135 16s
Relative Abundance
0.00
0.25
0.50
0.75
1.00
839
887
Sample
class
Bacteroidia
Betaproteobacteria
Clostridia
Gammaproteobacteria
Other
unclassified_Bacteria
Patient: 135
Percentage
0
25
50
75
100
0
5
10
15
20
Day
GO
biosynthetic process
carbohydrate metabolic process
cellular amino acid metabolic process
gluconeogenesis
glucose metabolic process
glycolytic process
iron−sulfur cluster assembly
metabolic process
transcription, DNA−templated
translation

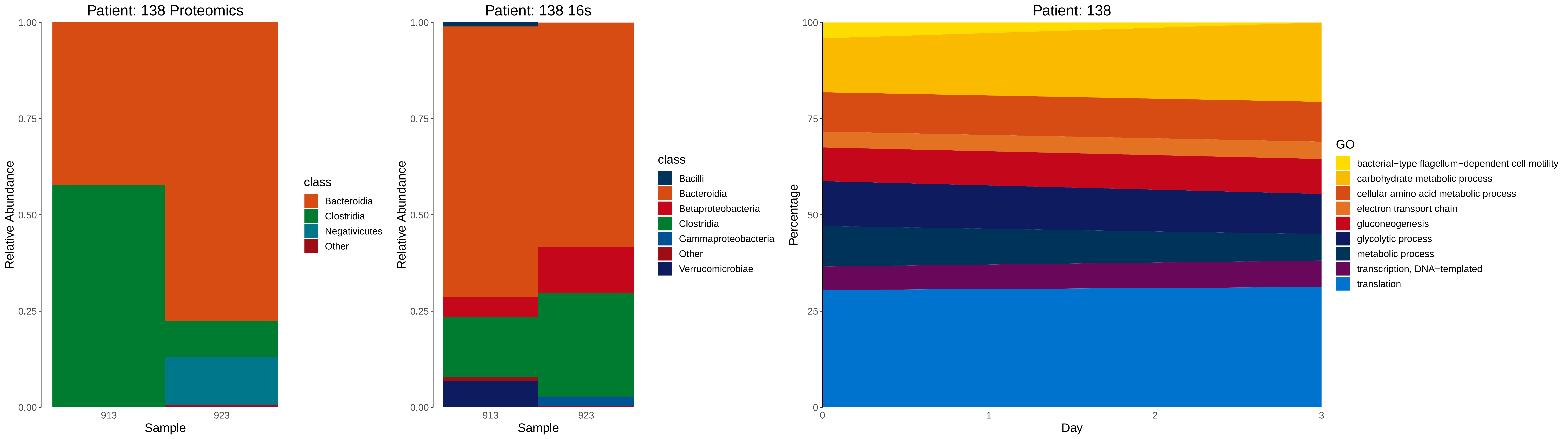
Patient: 138 Proteomics
Relative Abundance
1.00
0.75
0.50
0.25
0.00
913
923
Sample
class
Bacteroidia
Clostridia
Negativicutes
Other
Patient: 138 16s
Relative Abundance
1.00
0.75
0.50
0.25
0.00
913
923
Sample
class
Bacilli
Bacteroidia
Betaproteobacteria
Clostridia
Gammaproteobacteria
Other
Verrucomicrobiae
Patient: 138
Percentage
100
75
50
25
0
0
1
2
3
Day
GO
bacterial−type flagellum−dependent cell motility
carbohydrate metabolic process
cellular amino acid metabolic process
electron transport chain
gluconeogenesis
glycolytic process
metabolic process
transcription, DNA−templated
translation
